# A flexible framework for robust and efficient Mendelian randomization with debiasing

**DOI:** 10.1093/bib/bbag468

**Published:** 2026-09-07

**Authors:** Linsui Deng, Kejun He, Xianyang Zhang

**Affiliations:** Department of Probability and Statistics, School of Mathematics and Statistics, Central South University, 405 Xiaoxiang Middle Road, Yuelu District, Changsha, Hunan 410083, China; The Center for Applied Statistics, Institute of Statistics and Big Data, Renmin University of China, No. 59 Zhongguancun Street, Haidian District, Beijing 100872, China; Department of Statistics, Texas A&M University, 155 Ireland Street, College Station, TX 77843-3143, United States

**Keywords:** pleiotropy, measurement error, EM algorithm, generalized Bayesian information criterion

## Abstract

Mendelian randomization (MR) has been widely used to infer causal relationships between exposures and outcomes in epidemiological studies. However, classical MR assumptions can be violated when genetic variants are associated with outcomes through pathways other than the exposure, leading to uncorrelated and/or correlated pleiotropy. Additionally, measurement error arising from the inherent uncertainty in summary statistics obtained from large-scale genome-wide association studies can introduce bias into the causal effect estimate. To address these issues, we develop a debiased mixture inverse variance weighting ($\mathsf{dmIVW}$) method with three major advantages. First, it is capable of simultaneously handling various types of pleiotropy and eliminating the bias caused by uncertainty. Second, it can guard against distortion caused by invalid genetic variants while effectively harnessing their information. Third, our unified framework facilitates a fair comparison and combination of a series of submodels, encompassing several popular MR methods as special cases. Through real data applications, the effectiveness and robustness of $\mathsf{dmIVW}$ in estimating the causal effects of risk factors on common diseases are demonstrated.

## Introduction

Identifying causal relationships between exposures (e.g. risk factors) and outcomes (e.g. diseases) is crucial for understanding complex biological mechanisms in epidemiological studies [1, 2]. Correlations in observational data may arise from factors such as confounding, reverse causation, and other biases and therefore cannot be equated with causal relationships [3, 4].

Mendelian randomization (MR) has emerged as a popular method that uses genetic variants as instrumental variables (IVs) to infer the causal effect by leveraging summary statistics from genome-wide association studies (GWAS) [5–8]. To serve as valid IVs, the variants should satisfy three key assumptions: they must correlate with the exposure of interest (relevance), remain independent of confounding factors (independence), and influence the outcome exclusively through their effect on the exposure (exclusion restriction) [9].

**Figure 1:**
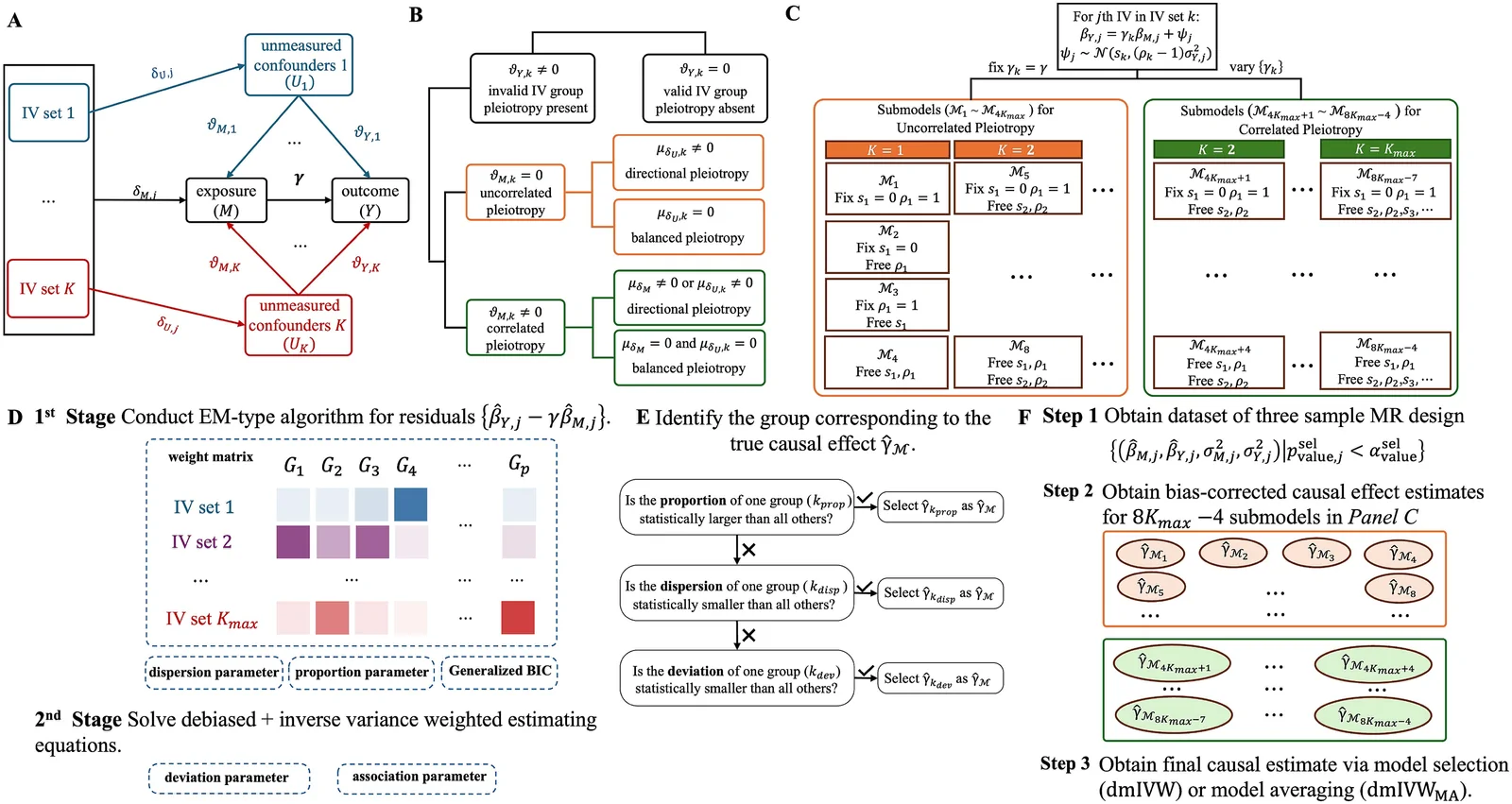
Overview of $\mathsf{dmIVW}$. $\mathsf{A}$: IVs are classified into *K* sets, where the IVs within *k*th set are possibly influenced by an unmeasured confounder $U_{k}$ with effect being $\delta _{U_{k}, j} \sim \mathcal{N}(\mu _{U_{k}}, \sigma _{U_{k}}^{2})$, the confounder $U_{k}$ exhibits different strengths to exposures $(\theta _{M,k})$ and outcomes $(\theta _{Y,k})$, the effect of IVs $G_{j}$ on exposure *M* follows $\delta _{M, j} \sim \mathcal{N}(\mu _{\delta _{M}}, \sigma _{\delta _{M}}^{2})$. $\mathsf{B}$: IVs within *k*th group can either be valid ($\theta _{Y,k} = 0$), suffer from uncorrelated pleiotropy ($\theta _{Y,k} \neq 0, \theta _{M,k} = 0$), or suffer from correlated pleiotropy ($\theta _{Y,k}, \theta _{M,k} \neq 0$), with both types of pleiotropies can be further classified as directional or balanced pleiotropies. $\mathsf{C}$: Illustration of $8K_{\max }-4$ submodels for uncorrelated pleiotropy and correlated pleiotropy, where $K_{\max }$ is the maximum number of examined groups and is set to three by default, $\gamma _{k}$ captures both the causal effect and the influence of IV-shared confounding, if it exists, due to correlated pleiotropy, and $s_{k}$ and $\rho _{k}$ model the directional and balanced pleiotropy, respectively. $\mathsf{D}$: Given submodel $\mathcal{M}_{8K_{max}-4}$, the two-stage estimation process and estimated parameters at each stage. $\mathsf{E}$: Given submodel $\mathcal{M}_{8K_{max}-4}$ and estimated parameters, the sequential causal group selection approach of determining the group corresponding to the causal effect. $\mathsf{F}$: Pipeline for obtaining the $\mathsf{dmIVW}$ and $\mathsf{dmIVW}_{\mathsf{MA}}$ estimators.

However, MR estimates can be biased by “pleiotropy” when genetic variants violate either the independence or exclusion restriction assumptions [6, 7, 10]. Pleiotropy is categorized into two main types: for “uncorrelated pleiotropy,” only the exclusion restriction is violated; while for “correlated pleiotropy,” both assumptions are violated. It can further be classified as directional or balanced depending on whether the pleiotropic effects are centered on zero (Fig. 1B). Invalid IVs with uncorrelated pleiotropy are easier to identify and may actually improve estimation efficiency [11], whereas those with correlated pleiotropy are difficult to detect and often coincide with multiple causal pathway scenarios [12–14]. To address these challenges, some robust methods have been developed [11, 12, 15–22], with mixture-model-based approaches achieving both robustness and efficiency by allowing various types of pleiotropy through the assumption that IVs originate from valid and invalid groups [11, 12, 18–21, 23].

Beyond pleiotropy concerns, a two-sample MR study (which estimates the IV-exposure and IV-outcome effects from two nonoverlapping GWAS datasets) faces challenges from measurement error in these estimated effects. This measurement error typically induces causal effect estimates biased toward zero, a phenomenon known as attenuation bias or weak IV bias [24–29]. A canonical remedy is to select IVs strongly correlated with the exposure [17, 30] or to assume no measurement error (NOME) [11, 17]. However, the strong IV selection approach typically introduces selection bias, while the NOME assumption rarely holds in practice. Recent methodological advances include the three-sample MR framework [26], which mitigates selection bias by using an additional independent dataset for IV selection (see Fig. 1F). Although this approach eliminates selection bias, the resulting procedure could be sensitive to the selection threshold. Alternative approaches have emerged to directly handle measurement error through various statistical techniques: incorporating measurement error into the likelihood function that either directly estimates IV-exposure effects [20], or profiles IV-exposure effects out [27], or applying debiasing corrections [28]. However, when these approaches are combined with mixture modeling, they do not adequately address balanced pleiotropy [20, 21]. Critically, our analyses demonstrate that even methods specifically designed to model measurement error or balanced pleiotropy often fail to sufficiently mitigate the induced attenuation and estimation bias.

**Figure 2:**
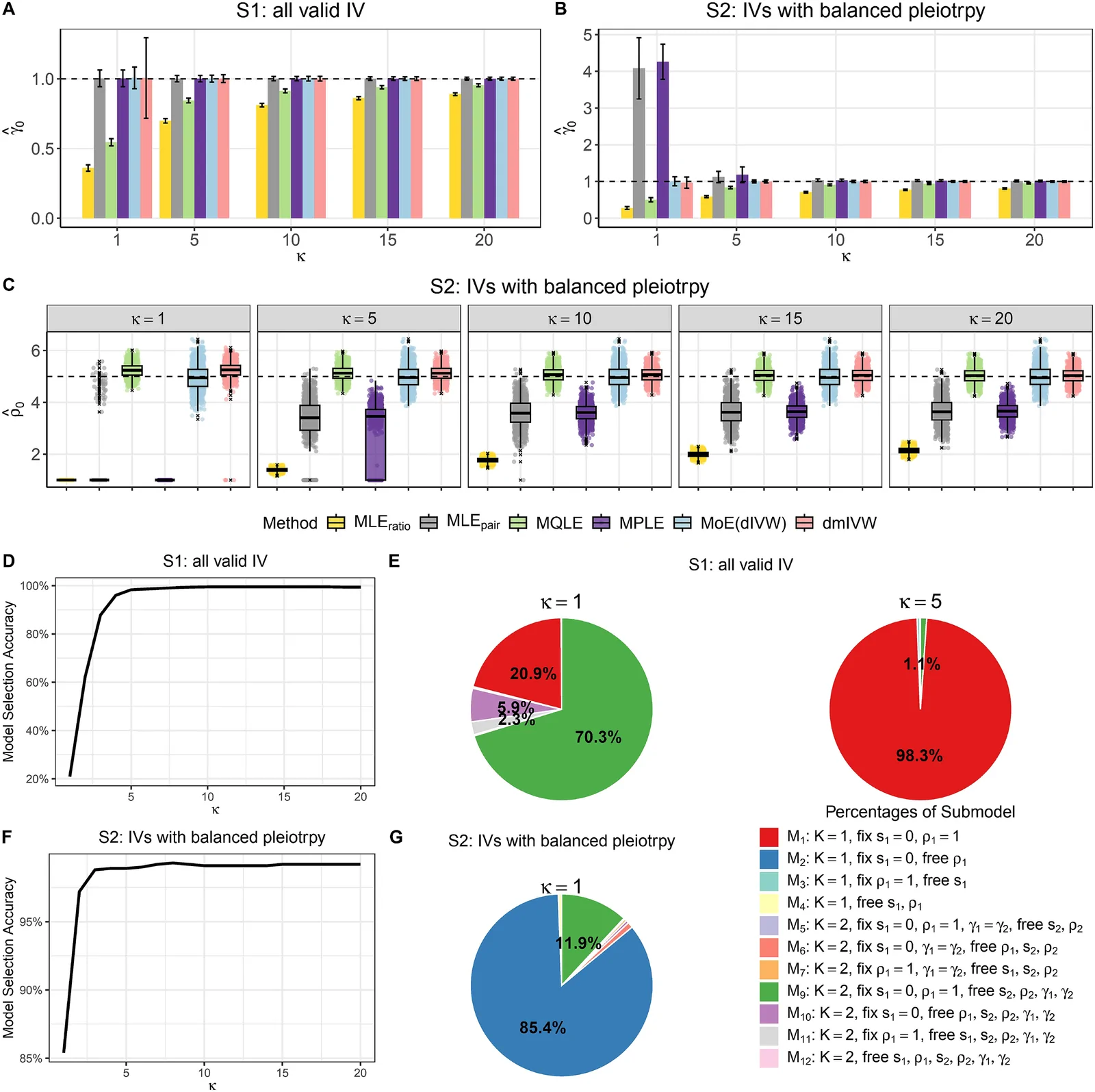
Comparisons of methods considering measurement error and balanced pleiotropy, and model selection results of $\mathsf{dmIVW}$ with $K_{\max }=2$. The synthetic data mimicked the BMI–BMI dataset, where the values of $\beta _{M,j}$ were proportional to those in the BMI–BMI dataset. These values were set to vary the average IV strength $\kappa = p^{-1} \sum _{j=1}^{p} {\beta _{M,j}^{2}}/{\sigma _{M,j}^{2}}$ from 1 to 20, with 1000 replications for each setting. Two scenarios were considered: (S1) all IVs were valid ($\mathcal{M}_{1}$: *K=1*, $s_{1}=0$, and $\rho _{1}=1$), and (S2) IVs exhibited balanced pleiotropy ($\mathcal{M}_{2}$: *K=1*, $s_{1}=0$, and $\rho _{1}=5$). ${\mathsf{A}}$ and ${\mathsf{B}}$: the mean (*± * 1 standard deviation) of causal effect estimates under S1 and S2. ${\mathsf{C}}$: the boxplot of estimated dispersion parameters under S2. ${\mathsf{D}}$ and ${\mathsf{F}}$: the model selection accuracies under S1 and S2. ${\mathsf{E}}$ and ${\mathsf{G}}$: the composition of selected submodels under S1 (*κ =1* and *κ =5*) and S2 (*κ =1*). The labels of the submodel refer to Fig. 1C.

**Figure 3:**
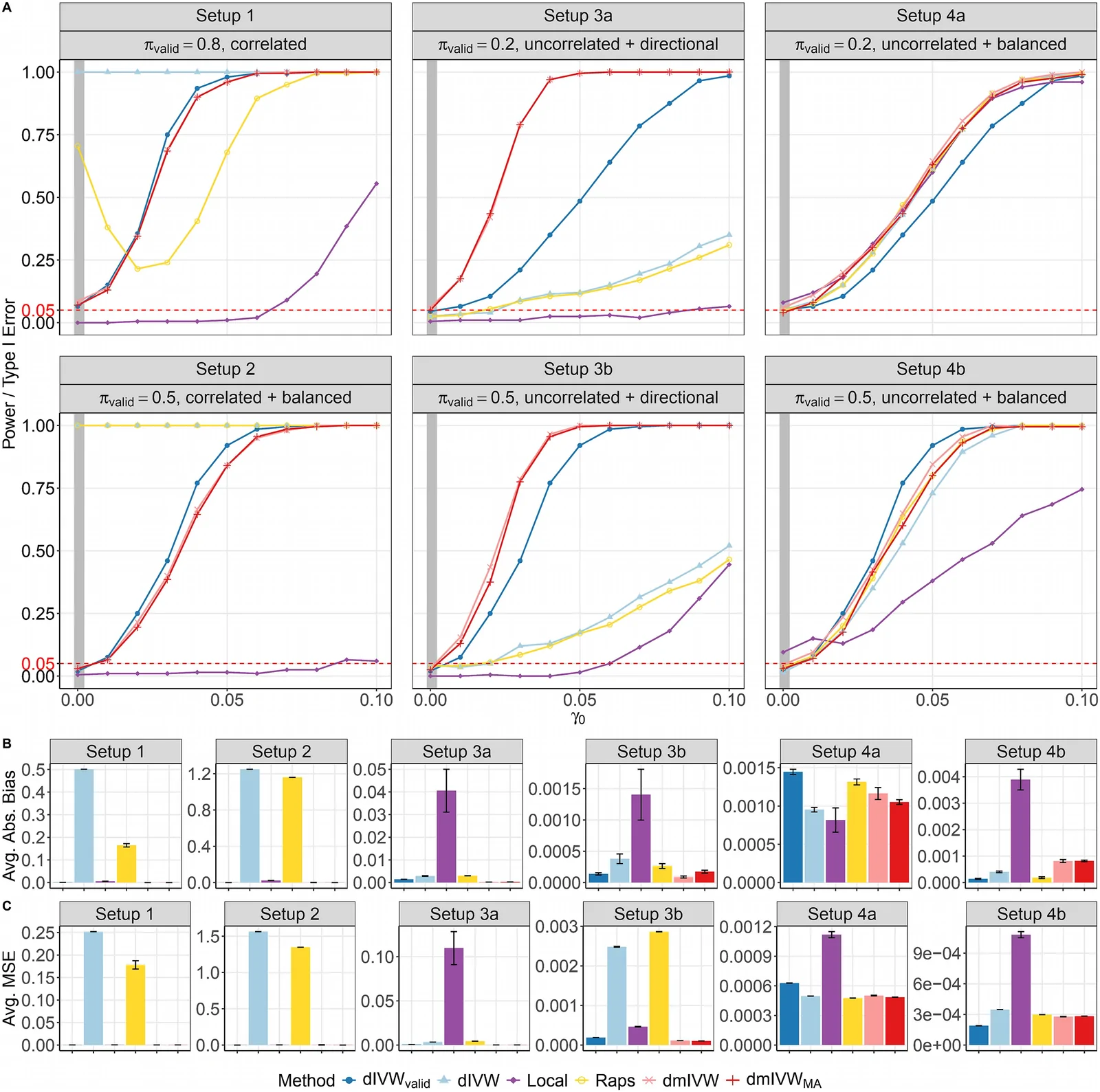
Comparisons of methods under two-group IV setups. The first group consisted of valid IVs, while the second group included IVs with various types of pleiotropy: correlated ($\gamma _{2}=\gamma _{1}-2.5$) and uncorrelated pleiotropy ($\gamma _{2}=\gamma _{1}$), as well as directional ($s_{2}=0.02$ and $\rho _{2}=1$) and balanced pleiotropy ($s_{2}=0$ and $\rho _{2}=5$). The simulation was repeated 200 times, and the significance level was set as 0.05. $\mathsf{A}$: the power ($\gamma _{0}> 0$) and Type I error ($\gamma _{0} = 0$) under the significance level 0.05 (dashed horizontal line).$\mathsf{B}$ and $\mathsf{C}$: the absolute bias and MSE, averaged over different values of $\gamma _{0}$ and all replications. Source data of Panels $\mathsf{B}$ and $\mathsf{C}$ are provided in Supplementary Table S6.

**Figure 4:**
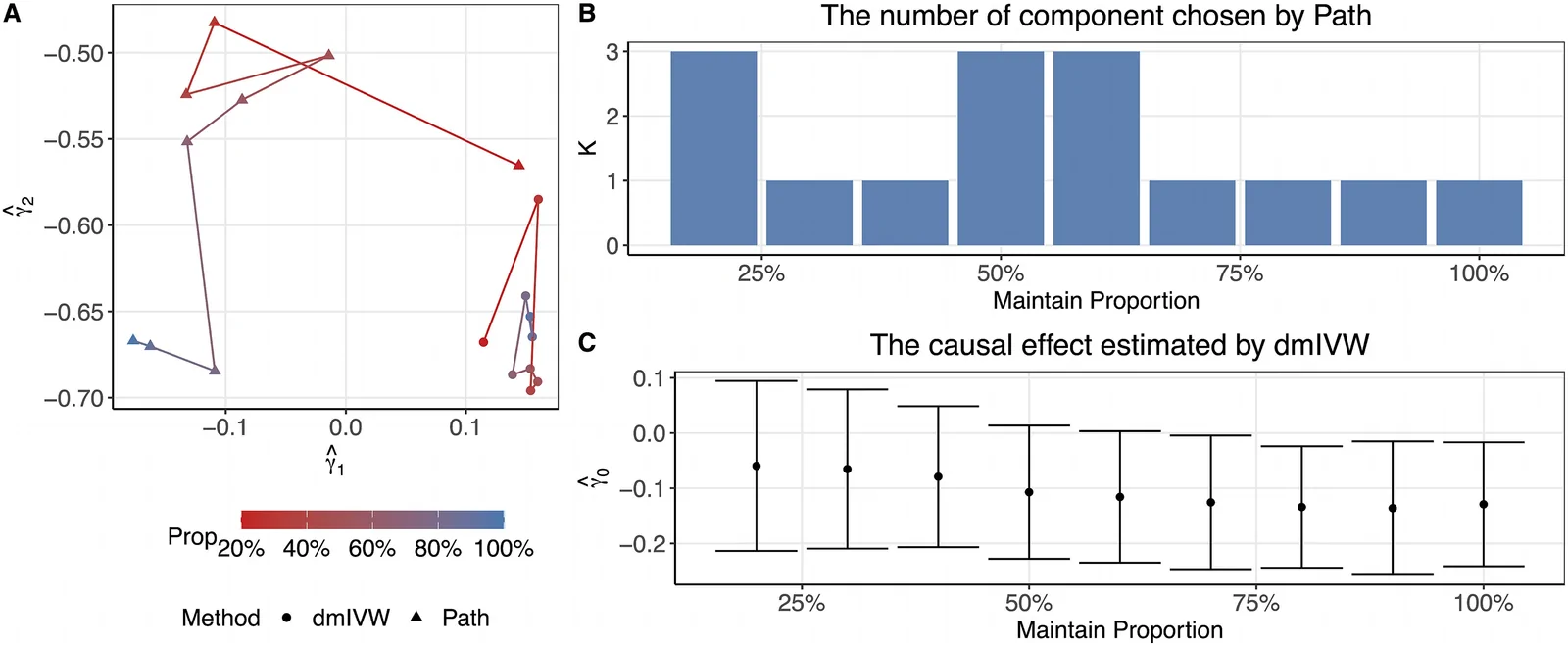
Sensitivity analysis of $\mathsf{dmIVW}$ and $\mathsf{Path}$ with varying P-value selection thresholds under the three-sample design. The P-value threshold was varied to retain between 20% and 100% of the SNPs. Details of the dataset are provided in Supplementary Table S14, and source data of results are provided in Supplementary Tables S10 and S11. $\mathsf{A}$: the two group effects, denoted by $\widehat{\gamma }_{1}$ and $\widehat{\gamma }_{2}$, estimated by $\mathsf{dmIVW}$ and $\mathsf{Path}$ with *K=2* at different thresholds. $\mathsf{B}$: the number of groups determined by $\mathsf{Path}$ at different thresholds. $\mathsf{C}$: the effects, denoted by $\gamma _{0}$, of HDL-C on CAD estimated by $\mathsf{dmIVW}$ across varying thresholds.

**Figure 5:**
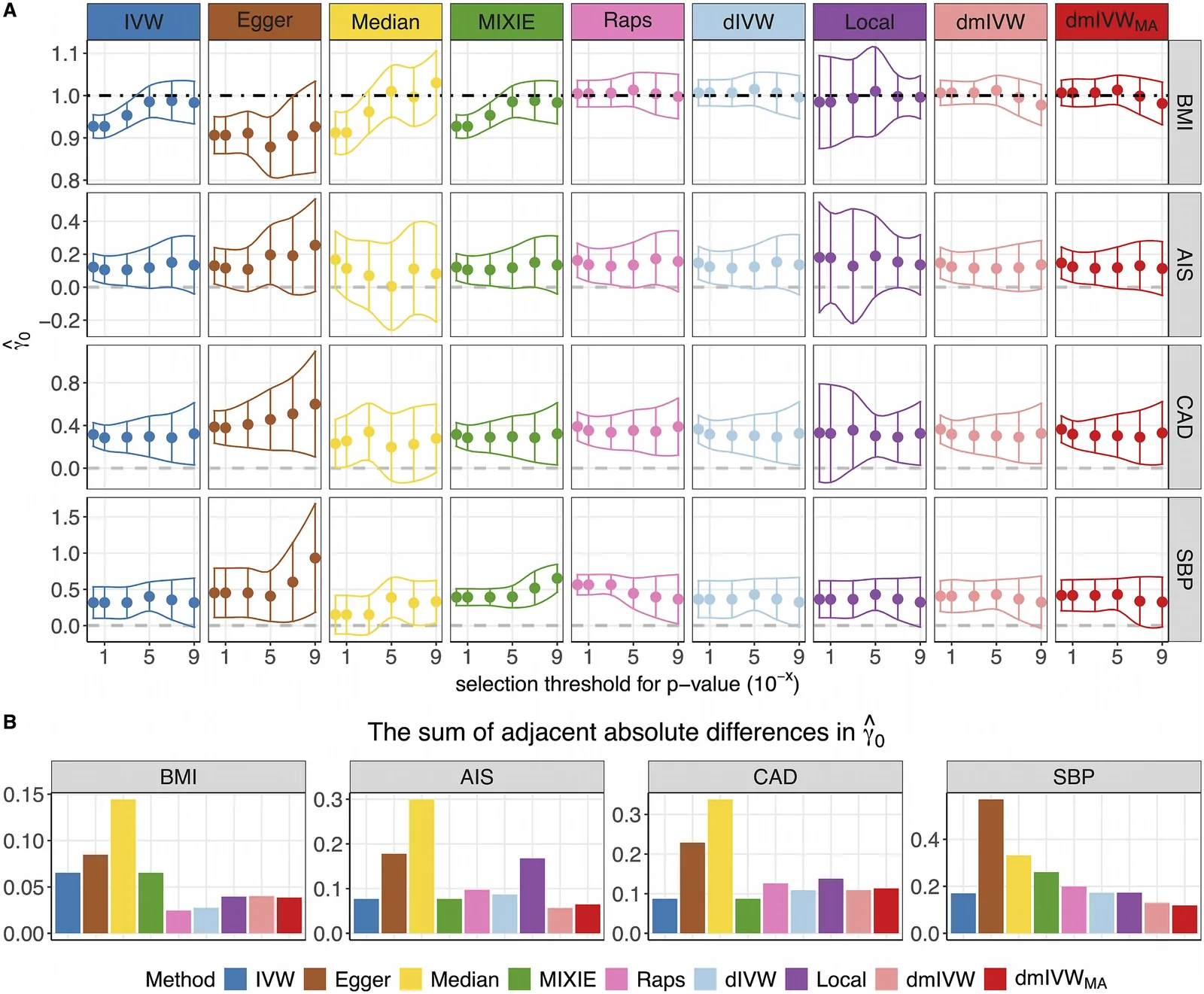
Comparison of estimated causal effects of BMI on AIS, CAD, and SBP across varying P-value thresholds. The causal effect is the logOR of AIS and CAD per SD-unit increase in BMI, and the SD-unit increase of BMI and SBP per SD-unit increase in BMI. Details of the dataset are provided in Supplementary Table S15, and source data are provided in Supplementary Tables S12 and S13. $\mathsf{A}$: the estimated causal effects $\widehat{\gamma }_{0}$ and the corresponding *95%* confidence interval at different selection thresholds. $\mathsf{B}$: the sum of adjacent absolute differences in the estimated causal effects $\widehat{\gamma }_{0}$, defined as $\sum _{i=2}^{6} |\widehat{\gamma }_{0,i} - \widehat{\gamma }_{0,i-1}|$, where $\widehat{\gamma }_{0,i}$ is the estimate using $\alpha ^{\mathrm{sel},1}=1$ and $\alpha ^{\mathrm{sel},i}=10^{-(2i-3)}$ for $2\leq i\leq 6$ as the P-value selection threshold.

**Figure 6:**
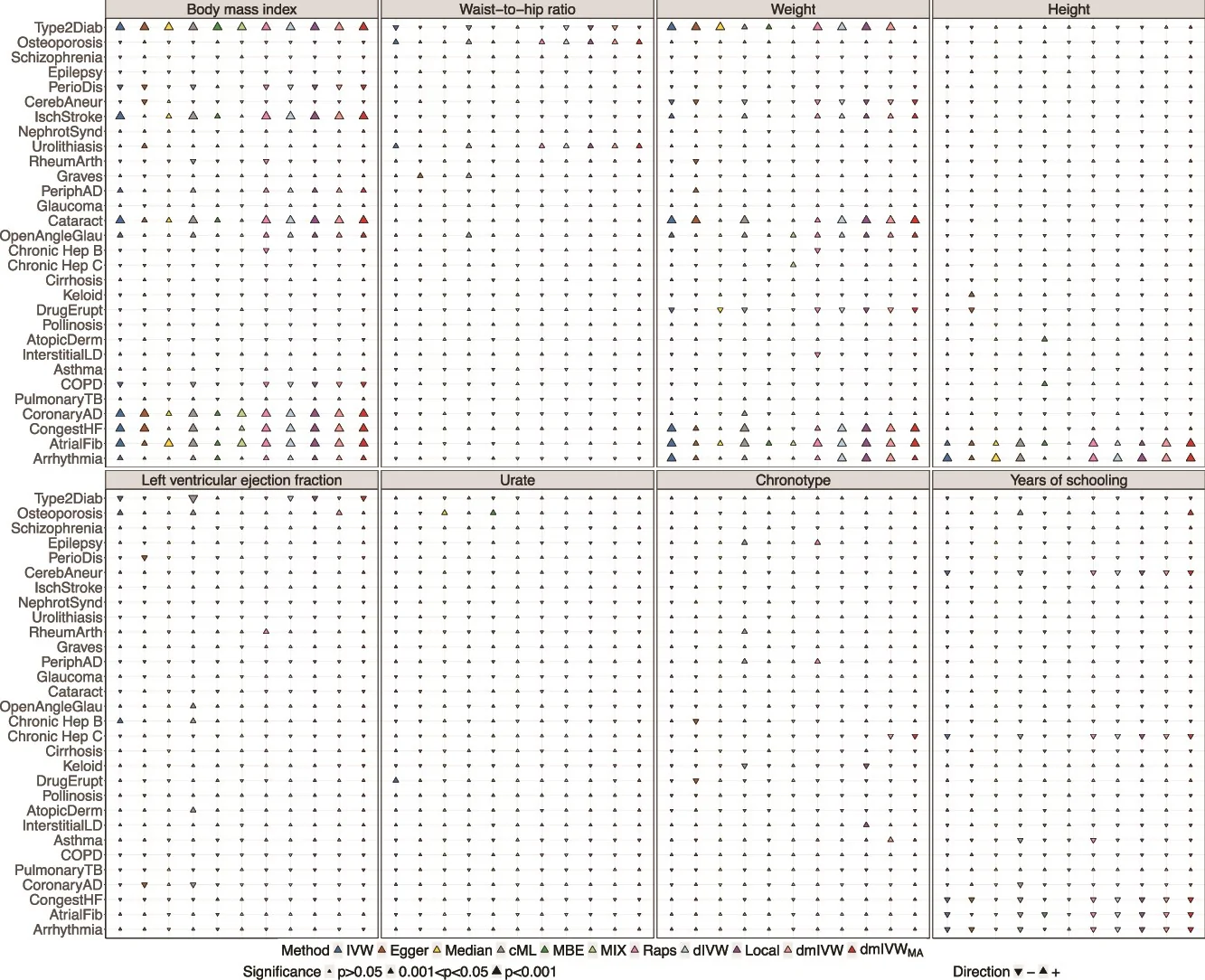
Results of various methods for detecting causal relationships between 8 exposures and 30 diseases using $10^{-5}$ as the P-value selection threshold. The causal effect is the logOR of disease per SD-unit increase in risk factor. Details of the dataset are provided in Supplementary Table S16, results using $10^{-3}$ and $10^{-4}$ as thresholds are provided in Supplementary Figs. S11 and S12 and source data of results are provided in Supplementary Tables S17–S19.

To address the challenges of pleiotropy and measurement error in MR studies, we propose the debiased mixture inverse variance weighting ($\mathsf{dmIVW}$) method, which classifies IVs into distinct groups while systematically correcting for measurement error bias. This approach integrates two major methodological innovations. First, by flexibly adjusting the number of groups and specifying pleiotropy forms (absent/present, balanced/directional, uncorrelated/correlated; see Fig. 1B), $\mathsf{dmIVW}$ creates a comprehensive framework encompassing various submodels. A significant advantage of $\mathsf{dmIVW}$ is its unified framework that enables model selection or model averaging through the generalized Bayesian information criterion (gBIC) [12, 31]. This provides a systematic and unified mechanism for evaluating and combining mainstream MR methods, many of which emerge as special cases within our framework. Second, each submodel employs a mixture model to estimate group membership probabilities for individual IVs [32], which then reweights the IVs in subsequent debiased estimating equations to correct for measurement error-induced bias. The two-stage approach disentangles and alleviates the dual challenges posed by pleiotropy and measurement error.

In this paper, we demonstrate that $\mathsf{dmIVW}$ outperforms existing methods in several aspects. First, we describe five baseline estimators, each representing a distinct strategy of modeling IV-exposure and IV-outcome effects. These strategies can naturally account for measurement error and balanced pleiotropy. Many robust methods have been developed based on these modeling strategies, which can be categorized into five types, namely $\mathsf{MLE}_{\mathsf{ratio}}$ [14, 18, 33], $\mathsf{MLE}_{\mathsf{pair}}$ [12, 20, 34], $\mathsf{MQLE}$ [23], $\mathsf{MPLE}$ [13, 27], and $\mathsf{MoE}$ [22, 28] (please refer to the Discussions section and Supplementary Section S4.3 for further details). Through comparative analysis, we show that $\mathsf{dmIVW}$ simultaneously eliminates the bias induced by both measurement error and balanced pleiotropy, whereas some of the five estimators can address at most one of the two sources of bias. The bias-eliminating property is achieved through our two-stage estimation approach. Second, $\mathsf{dmIVW}$ leverages information from invalid IVs with uncorrelated pleiotropy while maintaining robustness against correlated pleiotropy—a balance difficult to achieve with existing methods. Our proposed methods consistently rank in the top two tiers across various scenarios. The proposed model-averaging approach, which combines estimates from different submodels using gBIC-based weights, achieves first-tier performance when pleiotropy is uncorrelated. Third, our real data applications reveal $\mathsf{dmIVW}$’s stability in estimation and model selection across varying IV selection thresholds. When analyzing the relationship between high-density lipoprotein cholesterol (HDL-C) and coronary heart disease (CAD) using the original $\mathsf{Path}$ method [12], we observed that the number of groups in the selected models fluctuates considerably with different IV thresholds. In contrast, $\mathsf{dmIVW}$ consistently selects the one-group model with uncorrelated balanced pleiotropy. This stability in model selection enhances the credibility of our findings. Similarly, in studies using body mass index (BMI) as exposure, $\mathsf{dmIVW}$ exhibits substantially less variation compared with other methods [11, 15–18, 20, 22, 27, 28, 35] when the IV selection threshold changes. Finally, our method recovers established causal relationships, identifying BMI, weight, and height as risk factors and suggesting a protective effect of years of schooling. In contrast, the competing methods either suffer from reduced statistical power or produce inconsistent results across different IV selection thresholds.

## Results

### Overview of the proposed dmIVW method

We propose a general framework that can robustly and effectively estimate the causal effect, denoted by *γ *, of an exposure *M* on an outcome *Y* (see Supplementary Table S5 for a summary of the main notation). As depicted in the causal diagram (see Fig. 1A), we assume that the *p* IVs are classified into *K* distinct groups, $\{\mathcal{J}_{k}\}_{k=1}^{K}$, based on their interactions with unmeasured confounders. Due to privacy and data access limitations, we focus on settings where only summary statistics are available, where the true IV-exposure and IV-outcome associations for the *j*th IV are denoted by $\beta _{M,j}$ and $\beta _{Y,j}$, respectively. A typical scenario involves obtaining the estimated associations $\widehat{\beta }_{M,j}$ and $\widehat{\beta }_{Y,j}$, along with their standard errors $\sigma _{M,j}$ and $\sigma _{Y,j}$. These association estimates are, asymptotically, independently and normally distributed, i.e. $\widehat{\beta }_{M,j} \sim \mathcal{N}(\beta _{M,j}, \sigma ^{2}_{M,j})$ and $\widehat{\beta }_{Y,j} \sim \mathcal{N}(\beta _{Y,j}, \sigma ^{2}_{Y,j})$.

In the absence of pleiotropy, the true associations should ideally satisfy a linear relationship, $\beta _{Y,j} = \gamma \beta _{M,j}$. However, this assumption is often violated due to pleiotropic effects. To account for pleiotropy, we adopt a flexible working model that directly characterizes the relationship between IV-exposure and IV-outcome associations. For $j\in \mathcal{J}_{k}$, we write


(1) \begin{align*}& \beta_{Y,j} = \gamma_{k} \beta_{M,j} + \psi_{j}, \quad \psi_{j} \sim \mathcal{N}\left(s_{k}, (\rho_{k} - 1)\sigma^{2}_{Y,j}\right),\end{align*}

where $\gamma _{k}$ captures both the target causal effect *γ * and any bias introduced by IV-shared confounding (e.g. correlated pleiotropy). Here, $\psi _{j}$ represents the residual pleiotropic effect that is not explained by the linear relationship between $\beta _{M,j}$ and $\beta _{Y,j}$. Such effects can be categorized as balanced if $\psi _{j}$ is centered around zero, or as directional otherwise. The parameter $s_{k}$ models possible directional pleiotropic effects, while $\rho _{k}\geq 1$ serves as a dispersion parameter for balanced pleiotropy. The above working model is derived from the genetic structural equations (2) by decomposing the pleiotropic effect into components that are correlated with and orthogonal to $\beta _{M,j}$ (see details in the Method section). Model identifiability is discussed in Supplementary Section S5, where we show that the proposed working model is identifiable provided that the IV–exposure effects exhibit some heterogeneity within each group. As illustrated in Fig. 1B, the pleiotropic effects considered in the working model for each IV group correspond to specific scenarios in the causal diagram.

In model (1), we account for different types of pleiotropy by fixing the values of the parameters associated with pleiotropic effects. Specifically, we consider a total of $8K_{\max }-4$ submodels $\{\mathcal{M}_{m}\}$ with the number of IV groups *K* ranging between one and $K_{\max }$ being the maximum possible number of IV groups (see Fig. 1C). These submodels come from two types of sources: $4K_{\max }$ submodels for uncorrelated pleiotropy ($\mathcal{M}_{1}$ to $\mathcal{M}_{4K_{\max }}$), which assume $\gamma _{k}=\gamma $, and $4(K_{\max }-1)$ submodels for correlated pleiotropy ($\mathcal{M}_{4K_{\max }+1}$ to $\mathcal{M}_{8K_{\max }-4}$), where $\gamma _{k}$ vary across groups. For each type with a given *K*, we assume that the first group can be further classified as either valid ($s_{1} = 0$, $\rho _{1} = 1$), invalid with balanced pleiotropy ($s_{1} = 0$, $\rho _{1}> 1$), invalid with directional pleiotropy ($s_{1} \neq 0$, $\rho _{1} = 1$), or invalid with both ($s_{1} \neq 0$, $\rho _{1}> 1$).

Within each submodel $\mathcal{M}_{m}$, the parameter estimation consists of two stages (see Fig. 1D). In the first stage, a mixture model (*K≥ 2*) is applied to model the residuals $\widehat{\beta }_{Y,j}-\gamma \widehat{\beta }_{M,j}$, and an expectation-maximization (EM)-type algorithm is then employed to estimate model parameters, including the dispersion parameters for balanced pleiotropy $\{\widehat{\rho }_{k}\}$, the group proportions $\{\widehat{\pi }_{k}\}$, and the probabilities of *j*th IV belonging to *k*th group $\{\widehat{w}_{j,k}\}$. When *K=1*, the model degenerates to the single-group case, and a quasi-likelihood approach is adopted to estimate $\rho _{1}$ by modeling the residuals. In the second stage, we solve the debiased estimating equations to estimate $\{\widehat{\gamma }_{k}\}$ and deviation parameters for directional pleiotropy $\{\widehat{s}_{k}\}$. These equations correct for measurement error and weight IVs based on their relative probabilities and dispersed variances. The two stages play complementary roles in producing the final causal estimator (see the Methods section and Supplementary Section S1). First, when considering the one-group submodels ($\mathcal{M}_{1}\sim \mathcal{M}_{4}$), the two stages, respectively, correspond to existing approaches for adjusting the estimating equations for balanced pleiotropy [27] and for correcting biases caused by measurement error [28]. Second, the first stage provides the gBIC, which performs well in selecting the best submodel and in averaging the results from different submodels (see Figs 2 and 3). Third, although measurement error in residuals are considered in the first stage, the induced bias is not fully eliminated (see Fig. 3). The second stage is necessary to adjust for the attenuation bias properly.

When adopting a submodel with correlated pleiotropy, the effects $\{\gamma _{k}\}$ may differ across groups due to pleiotropic effects. Therefore, it is crucial to identify the group that most plausibly corresponds to the valid IVs, so that its estimated effect best approximates the true causal effect. Common assumptions in MR (e.g. majority rule, plurality rule, zero-modal pleiotropy assumption) require that valid IVs constitute the primary component among all IVs. Moreover, the valid IV group is expected to exhibit neither dispersion ($\rho _{k} = 1$) nor systematic deviation ($|s_{k}| = 0$). Guided by these principles, we identify the target group by sequentially comparing their proportions, dispersion levels, and deviation levels. Specifically, we first assess whether the component with the highest proportion (denoted by the $k^\star $th group) exhibits a significantly larger proportion than all other components. If this condition is satisfied, we identify $\widehat{\gamma }_{k^\star }$ as the estimated causal effect. Otherwise, we proceed to compare the remaining components based on their dispersion (favoring the component with the smallest $\rho _{k}$) and, if necessary, their deviation levels (favoring the component with the smaller $|s_{k}|$), in the same way (see Fig. 1E and Methods). In summary, a sequential procedure is conducted to select the most reliable estimate of the causal effect, with each step supported by a formal statistical test.

Our method follows a structured pipeline (see Fig. 1F). Two exposure datasets and one outcome dataset are collected: one exposure dataset (referred to as the selection dataset) is used to identify IVs that exhibit strong associations with the exposure, while the remaining exposure dataset and the outcome dataset are used to infer the causal relationship. We then fit submodels for both uncorrelated and correlated pleiotropy using our two-stage estimation. These submodels are integrated within a unified framework, where the gBIC is used for both model selection and model averaging [20, 36]. We use $\mathsf{dmIVW}_{\mathsf{MA}}$ to denote the estimator from model averaging and $\mathsf{dmIVW}$ for the estimator from model selection, and also the general framework.

### $\mathsf{dmIVW}$ correctly selects the model, maintains robustness, and improves efficiency

We conducted a series of simulations to evaluate the performance of our proposed method across four different scenarios. First, we designed the one-group IV setup, where either all IVs are valid or all exhibit balanced pleiotropy with identical dispersion level, to demonstrate that $\mathsf{dmIVW}$ effectively selected the appropriate submodel and successfully accounted for both weak IVs and balanced pleiotropy. Next, we extended to the two-group IV setup, where IVs were partitioned into two distinct groups: one composed of valid IVs and the other containing invalid IVs exhibiting either uncorrelated or correlated pleiotropy. In this setting, we showed that $\mathsf{dmIVW}$ maintained robustness in the presence of correlated pleiotropy while also gaining statistical power from IVs with uncorrelated pleiotropy. Third, we considered a five-group IV setup to investigate the impact of different choices of $K_{\max }$ on the performance of $\mathsf{dmIVW}$, thereby motivating the practical choice of $K_{\max }=3$. Finally, in the individual data-based simulation, we generated individual-level data and derived summary statistics through regression analyses to mimic realistic study conditions, under which $\mathsf{dmIVW}$ outperformed most existing methods.

*One-group IV simulation.* Numerous methods have been proposed to handle invalid IVs under various identifiability conditions, but most are extensions of one of the baseline estimators. Thus, when all IVs are valid or exhibit balanced pleiotropy, these methods will yield results similar to the baseline estimators. The baseline estimators, detailed in the section of Methods and Supplementary Section S4, include methods such as maximum likelihood estimator using ratio estimates ($\mathsf{MLE}_{\mathsf{ratio}}$) and using paired estimates ($\mathsf{MLE}_{\mathsf{pair}}$), profiled likelihood estimator ($\mathsf{MPLE}$), quasi-likelihood estimator ($\mathsf{MQLE}$), and the moment based estimator ($\mathsf{MoE}$), which is equivalent to the $\mathsf{dIVW}$ estimator [28]. Notably, measurement error is naturally incorporated within all estimators, but as will be shown, some still fail to eliminate the induced bias. To streamline comparisons, we limited our evaluation to these baseline estimators and our proposed $\mathsf{dmIVW}$ estimator.

When all IVs were valid, the $\mathsf{MLE}_{\mathsf{ratio}}$ and $\mathsf{MQLE}$ estimators suffered from weak IV bias, while the $\mathsf{MLE}_{\mathsf{pair}}$, $\mathsf{MPLE}$, $\mathsf{MoE}$, and $\mathsf{dmIVW}$ estimators showed relatively smaller bias (see Fig. 2A). Compared with $\mathsf{MoE}$, $\mathsf{dmIVW}$ exhibited large variation when the IV strength was weak. In this case, submodels with more groups were more likely to be selected (see Fig. 2D), which introduced additional uncertainty. When all IVs suffered from balanced pleiotropy and IV strength was weak, $\mathsf{dIVW}$ and $\mathsf{dmIVW}$ accurately estimated the causal effect, while $\mathsf{MLE}_{\mathsf{pair}}$ and $\mathsf{MPLE}$ were biased upward (see Fig. 2B). The biases of $\mathsf{MPLE}$ and $\mathsf{MLE}_{\mathsf{pair}}$ arose from underestimation of the degree of balanced pleiotropy (see Fig. 2C). $\mathsf{dmIVW}$ performed well because the gBIC selected submodel $\mathcal{M}_{2}$, the one-group submodel considering balanced pleiotropy, in most cases (see Fig. 2G). In both scenarios, the accuracy of model selection improved as IV strength increased (see Figs 2D and 2F). Even when the IV strength was weak, the selected submodel only introduced additional uncertainty but did not substantially impact bias.

*Two-group IV simulation.* Next, we extended our simulations to two-group IV setups, where one group consisted entirely of valid IVs, while the other group contained IVs that suffered from various types of pleiotropy. Building on the one-group IV simulation, we retained $\mathsf{dIVW}$ ($\mathsf{MoE}$ before) and $\mathsf{dmIVW}$, both of which could simultaneously address weak IV bias and balanced pleiotropy. We additionally incorporated three variants of $\mathsf{dIVW}$: $\mathsf{dmIVW}_{\mathsf{MA}}$, our model averaging estimator; $\mathsf{Local}$ [22], which identifies and removes invalid IVs by leveraging local distribution before applying $\mathsf{dIVW}$; and $\mathsf{dIVW}_{\mathsf{valid}}$, the $\mathsf{dIVW}$ estimator applied to the valid IV group, which serves as a highly competitive method in this simulation but is infeasible in practice. The $\mathsf{dIVW}_{\mathsf{valid}}$ acts as the oracle method when invalid IVs suffer from correlated pleiotropy, but can be less effective for uncorrelated pleiotropy because the excluded IVs may still be informative. Furthermore, we included $\mathsf{Raps}$ [27], a modified version of $\mathsf{MPLE}$, which can correctly estimate the dispersion parameter by adjusting the corresponding estimating equation and effectively address both weak IV bias and balanced pleiotropy.

We calculated the Type I error, power, averaged absolute bias, and averaged mean square error (MSE) to evaluate the performance (see Fig. 3). Overall, $\mathsf{dmIVW}$, $\mathsf{dmIVW}_{\mathsf{MA}}$, and $\mathsf{dIVW}_{\mathsf{valid}}$ achieved the smallest bias and MSE while controlling Type I error under the significance level 0.05. In contrast, $\mathsf{dIVW}$ and $\mathsf{Raps}$ exhibited significant bias under setups with correlated pleiotropy. The $\mathsf{Local}$ method showed very low power, likely due to overly conservative variance estimation. In Setups 1–3b, $\mathsf{dmIVW}$ or $\mathsf{dmIVW}_{\mathsf{MA}}$ consistently achieved the lowest bias, and at least one of the two methods was always among the top two with the smallest MSE across all setups (Supplementary Table S6). Notably, in Setups 3a and 3b, our methods significantly outperformed $\mathsf{dIVW}_{\mathsf{valid}}$, which relied solely on the valid group. This indicates that our methods are not only robust to pleiotropy but also capable of effectively extracting information from IVs with uncorrelated pleiotropy. In Setups 4a and 4b, which explore group-level heterogeneity in balanced pleiotropy, the power of our method was at least comparable with that of $\mathsf{dIVW}$. Our approach down-weights groups affected by stronger balanced pleiotropy in the final estimate, as reflected in the weights $1/(\rho _{k} \sigma _{Y,j}^{2})$ used in the estimating equation. Incorporating these weights, which adjust for both within-group uncertainty and pleiotropic variability, results in more efficient estimation than the conventional $\mathsf{dIVW}$ weights $1/\sigma _{Y,j}^{2}$ when heterogeneity exists across groups.

Finally, we examined the computational cost under Setup S1 and found that the proposed method remains computationally feasible for problems with hundreds of instruments (see Supplementary Section S6.5 for details).

*Five-group IV simulation.* We further examined the impact of different choices of $K_{\max }$ on coverage probability and the accuracy of model selection, causal group selection, and causal effect estimation. We considered a setting with five IV groups (i.e. the true number of *K* is 5), consisting of one causal group, three with correlated pleiotropy, and one exhibiting either correlated or uncorrelated pleiotropy. We then applied our dmIVW procedure with various $K_{\max }$. Using $K_{\max }=5$ tended to select submodels with fewer groups (Supplementary Figs S2–S4). The selected submodels often merged groups with similar pleiotropic effects into a single group while still separating the causal group from pleiotropic groups (Supplementary Fig. S5). Our causal group selection procedure correctly identified the causal group in most replications (Supplementary Table S2). On the other hand, using $K_{\max }=3$ could capture the main heterogeneity in pleiotropic effects, which was supported by the accurate estimates with near-nominal coverage observed in Supplementary Fig. S1. Since the computational cost increases with $K_{\max }$ (Supplementary Section S6.5), we recommend using $K_{\max }=3$ in practice to balance estimation accuracy and computational efficiency. A detailed discussion is provided in Supplementary Section S6.1.

*Individual data simulation.* The final simulation began by generating individual-level data and then calculating the IV-exposure and IV-outcome effects through regression. We comprehensively compared our methods with 12 different methods: $\mathsf{IVW}$ [35], $\mathsf{dIVW}$ [28], $\mathsf{Egger}$ [15], $\mathsf{Raps}$ [27], $\mathsf{Median}$ [16], $\mathsf{MBE}$ [17], $\mathsf{APSS}$ [37], $\mathsf{cML}$ [20], $\mathsf{Local}$ [22], $\mathsf{MIX}$ [23], $\mathsf{MIXIE}$ [11], and $\mathsf{ConMIX}$ [18]. In general, the performance of $\mathsf{dmIVW}$ and $\mathsf{dmIVW}_{\mathsf{MA}}$ ranked in the first and second tiers across all scenarios (Table 1). $\mathsf{dmIVW}_{\mathsf{MA}}$ ranked in the top tier most frequently, with 22 out of 27 instances. The coverage of $\mathsf{dmIVW}_{\mathsf{MA}}$ was satisfactory, while $\mathsf{dmIVW}$ had lower coverage, possibly due to ignoring the model selection effect. While $\mathsf{MIX}$ achieved the smallest bias and RMSE most of the time when $\gamma _{0} = 0$, it suffered from very low power (Supplementary Table S8). When IVs exhibited uncorrelated pleiotropy, our method achieved first-tier performance. In cases of correlated pleiotropy, $\mathsf{dIVW}$, $\mathsf{Egger}$, $\mathsf{Raps}$, and $\mathsf{Local}$ were as sensitive as the base $\mathsf{IVW}$ estimator. Our methods reduced the bias introduced by invalid IVs to some extent. Among the methods (all criteria colored in blue and purple) robust to correlated pleiotropy, $\mathsf{dmIVW}$ and $\mathsf{dmIVW}_{\mathsf{MA}}$ were the only two that correctly estimated the causal effect of BMI on BMI in our second real data application with weak IVs.

**Table 1:** Comparisons of methods under individual data simulation with $\gamma _{0}=0$. Results were based on 1000 replications, summarizing bias (multiplied by 100 for ease of comparison), root mean square error (RMSE), and coverage (i.e. 1-Type I error here). We considered *30* IVs here, and $p_{\mathrm{invalid}}$ is the number of invalid IVs. The confidence level was set as *95%*. We colored the results in the first, second, and third tiers with blue, purple, and red, respectively, and counted the total number of each method falling into these three tiers. For each scenario, methods with absolute bias below twice or ten times the second smallest absolute bias were classified as first- and second-tier, respectively, with the rest in the third tier. RMSE was categorized using the same rule. For coverage, we account for variability arising from simulation due to finite samples: methods achieving at least 93.8% (which is determined by the 5% lower quantile of the Binomial(1000, 0.95) with 1000 being the number of replications and 0.95 being the coverage probability) were classified as first-tier. Methods exceeding 70% were classified as second-tier, while the rest are in the third tier.

Pleiotropy	$p_{\mathrm{invalid}}$	Criterion	Method													
			$\mathsf{IVW}$	$\mathsf{dIVW} $	$\mathsf{Egger}$	$\mathsf{Raps}$	$\mathsf{Median}$	$\mathsf{MBE}$	$\mathsf{APSS}$	$\mathsf{cML}$	$\mathsf{Local}$	$\mathsf{MIX}$	$\mathsf{MIXIE}$	$\mathsf{ConMIX}$	$\mathsf{dmIVW}$	$\mathsf{dmIVW}_{\mathsf{MA}}$
			${\textcolor{blue}{7}}/{\textcolor{purple}{13}}/{\textcolor{red}{7}}$	${\textcolor{blue}{7}}/{\textcolor{purple}{13}}/{\textcolor{red}{7}}$	${\textcolor{blue}{4}}/{\textcolor{purple}{19}}/{\textcolor{red}{4}} $	${\textcolor{blue}{3}}/{\textcolor{purple}{10}}/{\textcolor{red}{14}}$	${\textcolor{blue}{13}}/{\textcolor{purple}{9}}/{\textcolor{red}{5}}$	${\textcolor{blue}{22}}/{\textcolor{purple}{4}}/{\textcolor{red}{1}}$	${\textcolor{blue}{7}}/{\textcolor{purple}{13}}/{\textcolor{red}{7}}$	${\textcolor{blue}{16}}/{\textcolor{purple}{9}}/{\textcolor{red}{2}}$	${\textcolor{blue}{7}}/{\textcolor{purple}{13}}/{\textcolor{red}{7}}$	${\textcolor{blue}{19}}/{\textcolor{purple}{7}}/{\textcolor{red}{1}}$	${\textcolor{blue}{18}}/{\textcolor{purple}{8}}/{\textcolor{red}{1}}$	${\textcolor{blue}{16}}/{\textcolor{purple}{10}}/{\textcolor{red}{1}}$	${\textcolor{blue}{14}}/{\textcolor{purple}{13}}/{\textcolor{red}{0}}$	${\textcolor{blue}{22}}/{\textcolor{purple}{5}}/{\textcolor{red}{0}}$
		Bias(*× *100)	${\textcolor{blue}{-0.002}}$	${\textcolor{blue}{-0.002}}$	${\textcolor{red}{0.036}}$	${\textcolor{purple}{-0.014}}$	${\textcolor{red}{-0.032}}$	${\textcolor{red}{0.030}}$	${\textcolor{blue}{-\textbf{0.000}}}$	${\textcolor{purple}{-0.007}}$	${\textcolor{blue}{-0.002}}$	${\textcolor{red}{-0.052}}$	${\textcolor{blue}{0.003}}$	${\textcolor{red}{-0.035}}$	${\textcolor{purple}{-0.011}}$	${\textcolor{blue}{-0.002}}$
−	0	RMSE	${\textcolor{blue}{\textbf{0.011}}}$	${\textcolor{blue}{0.011}}$	${\textcolor{purple}{0.026}}$	${\textcolor{blue}{0.012}}$	${\textcolor{blue}{0.016}} $	${\textcolor{purple}{0.026}}$	${\textcolor{blue}{0.011}}$	${\textcolor{blue}{0.012}}$	${\textcolor{blue}{0.011}}$	${\textcolor{purple}{0.059}}$	${\textcolor{blue}{0.012}}$	${\textcolor{blue}{0.016}}$	${\textcolor{blue}{0.019}}$	$ {\textcolor{blue}{0.017}}$
		Coverage(%)	${\textcolor{blue}{95.80}}$	${\textcolor{blue}{95.70}}$	${\textcolor{blue}{95.30}}$	${\textcolor{blue}{96.00}}$	${\textcolor{blue}{98.40}}$	${\textcolor{blue}{99.20}}$	${\textcolor{blue}{96.00}}$	${\textcolor{blue}{94.80}}$	${\textcolor{blue}{95.70}}$	${\textcolor{purple}{93.20}}$	$ {\textcolor{blue}{94.40}}$	${\textcolor{purple}{87.20}}$	${\textcolor{purple}{90.30}} $	${\textcolor{blue}{96.90}}$
Directional uncorrelated		Bias(*× *100)	${\textcolor{red}{7.080}}$	${\textcolor{red}{7.110}} $	${\textcolor{blue}{-0.099}} $	${\textcolor{red}{7.996}}$	${\textcolor{red}{2.321}}$	${\textcolor{purple}{0.260}} $	${\textcolor{red}{7.147}}$	${\textcolor{red}{0.634}}$	${\textcolor{red}{7.110}}$	${\textcolor{purple}{0.458}}$	${\textcolor{purple}{0.373}}$	${\textcolor{purple}{0.315}}$	${\textcolor{blue}{\textbf{0.027}}}$	$ {\textcolor{blue}{0.059}}$
	6	RMSE	${\textcolor{purple}{0.074}}$	${\textcolor{purple}{0.074}}$	${\textcolor{purple}{0.080}}$	$ {\textcolor{red}{0.321}} $	$ {\textcolor{blue}{0.030}} $	$ {\textcolor{blue}{0.020}}$	${\textcolor{purple}{0.075}} $	${\textcolor{blue}{0.015}} $	${\textcolor{purple}{0.074}}$	${\textcolor{blue}{0.018}} $	$ {\textcolor{blue}{\textbf{0.015}}}$	${\textcolor{blue}{0.016}} $	${\textcolor{blue}{0.027}}$	${\textcolor{blue}{0.023}}$
		Coverage(%)	${\textcolor{red}{50.30}} $	$ {\textcolor{red}{50.70}}$	${\textcolor{purple}{92.40}}$	${\textcolor{purple}{79.70}}$	$ {\textcolor{purple}{85.50}}$	${\textcolor{blue}{98.70}} $	${\textcolor{red}{51.90}} $	${\textcolor{purple}{89.80}}$	$ {\textcolor{red}{50.70}}$	${\textcolor{blue}{95.70}} $	${\textcolor{purple}{92.10}}$	${\textcolor{purple}{87.90}}$	${\textcolor{purple}{89.40}}$	${\textcolor{blue}{96.70}}$
		Bias(*× *100)	${\textcolor{red}{19.910}}$	${\textcolor{red}{19.993}}$	${\textcolor{blue}{\textbf{0.230}}}$	${\textcolor{red}{22.912}}$	${\textcolor{red}{7.909}}$	${\textcolor{purple}{1.142}}$	${\textcolor{red}{20.037}}$	${\textcolor{purple}{1.204}}$	${\textcolor{red}{19.993}}$	${\textcolor{purple}{0.848}}$	${\textcolor{purple}{0.902}}$	${\textcolor{blue}{0.643}}$	${\textcolor{blue}{0.333}}$	${\textcolor{blue}{0.339}}$
	12	RMSE	$ {\textcolor{purple}{0.202}}$	${\textcolor{purple}{0.203}}$	${\textcolor{purple}{0.143}}$	${\textcolor{red}{0.418}}$	$ {\textcolor{purple}{0.087}} $	${\textcolor{blue}{0.027}}$	${\textcolor{purple}{0.203}}$	${\textcolor{blue}{0.021}} $	${\textcolor{purple}{0.203}}$	${\textcolor{blue}{0.021}}$	${\textcolor{blue}{0.029}} $	${\textcolor{blue}{\textbf{0.019}}}$	${\textcolor{blue}{0.034}}$	${\textcolor{blue}{0.028}}$
		Coverage(%)	${\textcolor{red}{3.70}}$	${\textcolor{red}{4.20}}$	${\textcolor{purple}{93.00}}$	${\textcolor{red}{12.70}}$	${\textcolor{red}{36.90}}$	${\textcolor{blue}{95.50}}$	${\textcolor{red}{3.90}}$	${\textcolor{purple}{84.00}}$	${\textcolor{red}{4.20}}$	${\textcolor{blue}{93.90}}$	${\textcolor{purple}{87.70}}$	${\textcolor{purple}{85.90}}$	${\textcolor{purple}{88.70}}$	${\textcolor{blue}{97.20}}$
Directional correlated		Bias(*× *100)	${\textcolor{purple}{4.678}}$	${\textcolor{purple}{4.696}}$	$ {\textcolor{purple}{4.347}}$	${\textcolor{purple}{2.253}} $	${\textcolor{purple}{2.151}}$	${\textcolor{blue}{0.734}}$	${\textcolor{purple}{4.700}}$	$ {\textcolor{purple}{1.602}}$	${\textcolor{purple}{4.696}} $	${\textcolor{blue}{\textbf{0.246}}}$	${\textcolor{blue}{0.963}}$	${\textcolor{blue}{0.716}}$	${\textcolor{blue}{1.232}}$	${\textcolor{blue}{1.356}}$
	6	RMSE	${\textcolor{purple}{0.049}}$	${\textcolor{purple}{0.049}}$	${\textcolor{purple}{0.063}}$	${\textcolor{blue}{0.029}}$	${\textcolor{blue}{0.028}}$	${\textcolor{blue}{0.023}}$	${\textcolor{purple}{0.049}}$	${\textcolor{blue}{0.023}}$	${\textcolor{purple}{0.049}}$	${\textcolor{blue}{\textbf{0.017}}}$	${\textcolor{blue}{0.023}}$	${\textcolor{blue}{0.020}}$	${\textcolor{blue}{0.033}}$	${\textcolor{blue}{0.031}}$
		Coverage(%)	${\textcolor{red}{31.33}}$	${\textcolor{red}{32.03}}$	${\textcolor{purple}{82.08}}$	${\textcolor{red}{66.17}}$	${\textcolor{purple}{85.29}}$	${\textcolor{blue}{97.80}}$	${\textcolor{red}{34.33}}$	${\textcolor{purple}{72.27}}$	${\textcolor{red}{32.03}}$	${\textcolor{blue}{95.40}}$	${\textcolor{purple}{82.68}}$	${\textcolor{purple}{82.68}}$	${\textcolor{purple}{81.98}}$	${\textcolor{purple}{92.19}}$
		Bias(*× *100)	${\textcolor{purple}{12.380}}$	${\textcolor{purple}{12.422}}$	${\textcolor{purple}{13.055}}$	${\textcolor{purple}{10.727}}$	${\textcolor{purple}{6.320}}$	${\textcolor{blue}{2.181}}$	${\textcolor{purple}{12.431}}$	${\textcolor{blue}{3.076}}$	${\textcolor{purple}{12.422}}$	${\textcolor{blue}{\textbf{0.778}}}$	${\textcolor{blue}{2.156}}$	${\textcolor{blue}{2.076}}$	${\textcolor{blue}{3.396}}$	${\textcolor{blue}{3.460}}$
	12	RMSE	${\textcolor{purple}{0.125}}$	${\textcolor{purple}{0.125}}$	${\textcolor{purple}{0.144}}$	${\textcolor{purple}{0.110}}$	${\textcolor{blue}{0.069}}$	${\textcolor{blue}{0.045}}$	${\textcolor{purple}{0.125}}$	${\textcolor{blue}{0.039}}$	${\textcolor{purple}{0.125}}$	${\textcolor{blue}{\textbf{0.023}}}$	${\textcolor{blue}{0.040}}$	${\textcolor{blue}{0.051}}$	${\textcolor{blue}{0.074}}$	${\textcolor{blue}{0.065}}$
		Coverage(%)	${\textcolor{red}{0.00}}$	${\textcolor{red}{0.00}}$	${\textcolor{red}{50.80}}$	${\textcolor{red}{1.70}}$	${\textcolor{red}{30.50}}$	${\textcolor{blue}{94.60}}$	${\textcolor{red}{0.00}}$	${\textcolor{red}{51.40}}$	${\textcolor{red}{0.00}}$	${\textcolor{purple}{90.90}}$	${\textcolor{red}{69.30}}$	${\textcolor{purple}{78.50}}$	${\textcolor{purple}{70.10}}$	${\textcolor{purple}{85.40}}$
Balanced uncorrelated		Bias(*× *100)	${\textcolor{blue}{-0.028}}$	${\textcolor{blue}{-0.029}}$	${\textcolor{purple}{0.070}}$	${\textcolor{red}{0.254}}$	${\textcolor{purple}{0.086}}$	${\textcolor{blue}{0.041}}$	${\textcolor{blue}{-0.023}}$	${\textcolor{blue}{0.034}}$	${\textcolor{blue}{-0.029}}$	${\textcolor{purple}{0.069}}$	${\textcolor{blue}{0.040}}$	${\textcolor{blue}{\textbf{0.022}}}$	${\textcolor{blue}{-0.031}}$	${\textcolor{blue}{-0.045}}$
	6	RMSE	${\textcolor{purple}{0.038}}$	${\textcolor{purple}{0.039}}$	${\textcolor{purple}{0.086}}$	${\textcolor{purple}{0.130}}$	${\textcolor{blue}{0.020}}$	${\textcolor{blue}{0.020}}$	${\textcolor{purple}{0.038}}$	${\textcolor{blue}{\textbf{0.014}}}$	${\textcolor{purple}{0.039}}$	${\textcolor{blue}{0.018}}$	${\textcolor{blue}{0.014}}$	${\textcolor{blue}{0.015}}$	${\textcolor{blue}{0.022}}$	${\textcolor{blue}{0.021}}$
		Coverage(%)	${\textcolor{blue}{94.60}}$	${\textcolor{blue}{94.90}}$	${\textcolor{purple}{93.00}}$	${\textcolor{purple}{91.40}}$	${\textcolor{blue}{96.20}}$	${\textcolor{blue}{98.70}}$	${\textcolor{blue}{95.70}}$	${\textcolor{blue}{94.20}}$	${\textcolor{blue}{94.90}}$	${\textcolor{blue}{96.50}}$	${\textcolor{blue}{94.50}}$	${\textcolor{purple}{88.90}}$	${\textcolor{purple}{93.70}}$	${\textcolor{blue}{98.00}}$
		Bias(*× *100)	${\textcolor{purple}{-0.221}}$	${\textcolor{purple}{-0.222}}$	${\textcolor{purple}{0.411}}$	${\textcolor{red}{0.810}}$	${\textcolor{blue}{-0.052}}$	${\textcolor{blue}{\textbf{-0.009}}}$	${\textcolor{purple}{-0.160}}$	${\textcolor{blue}{0.053}}$	${\textcolor{purple}{-0.222}}$	${\textcolor{purple}{0.119}}$	${\textcolor{blue}{0.079}}$	${\textcolor{blue}{0.069}}$	${\textcolor{blue}{0.048}}$	${\textcolor{blue}{0.049}}$
	12	RMSE	${\textcolor{purple}{0.074}}$	${\textcolor{purple}{0.075}}$	${\textcolor{purple}{0.163}}$	${\textcolor{red}{0.274}}$	${\textcolor{blue}{0.027}}$	${\textcolor{blue}{0.023}}$	${\textcolor{purple}{0.072}}$	${\textcolor{blue}{0.018}}$	${\textcolor{purple}{0.075}}$	${\textcolor{blue}{0.020}}$	${\textcolor{blue}{\textbf{0.018}}}$	${\textcolor{blue}{0.018}}$	${\textcolor{blue}{0.029}}$	${\textcolor{blue}{0.028}}$
		Coverage(%)	${\textcolor{blue}{94.90}}$	${\textcolor{blue}{94.90}}$	${\textcolor{blue}{94.20}}$	${\textcolor{purple}{85.50}}$	${\textcolor{purple}{92.50}}$	${\textcolor{blue}{97.60}}$	${\textcolor{blue}{96.30}}$	${\textcolor{purple}{90.50}}$	${\textcolor{blue}{94.90}}$	${\textcolor{blue}{95.50}}$	${\textcolor{purple}{92.50}}$	${\textcolor{purple}{88.20}}$	${\textcolor{purple}{93.10}}$	${\textcolor{blue}{97.90}}$
Balanced correlated		Bias(*× *100)	${\textcolor{purple}{1.161}}$	${\textcolor{purple}{1.165}}$	${\textcolor{red}{3.183}}$	${\textcolor{red}{6.565}}$	${\textcolor{blue}{0.292}}$	${\textcolor{blue}{0.213}}$	${\textcolor{purple}{1.155}}$	${\textcolor{blue}{0.267}}$	${\textcolor{purple}{1.165}}$	${\textcolor{blue}{\textbf{-0.012}}}$	${\textcolor{blue}{0.311}}$	${\textcolor{blue}{0.166}}$	${\textcolor{purple}{0.695}}$	${\textcolor{purple}{0.868}}$
	6	RMSE	${\textcolor{blue}{0.026}}$	${\textcolor{blue}{0.026}}$	${\textcolor{purple}{0.060}}$	${\textcolor{red}{1.941}}$	${\textcolor{blue}{0.019}}$	${\textcolor{blue}{0.027}}$	${\textcolor{blue}{0.026}}$	${\textcolor{blue}{\textbf{0.015}}}$	${\textcolor{blue}{0.026}}$	${\textcolor{blue}{0.019}}$	${\textcolor{blue}{0.021}}$	${\textcolor{blue}{0.016}}$	${\textcolor{blue}{0.031}}$	${\textcolor{blue}{0.028}}$
		Coverage(%)	${\textcolor{purple}{90.40}}$	${\textcolor{purple}{90.60}}$	${\textcolor{purple}{87.40}}$	${\textcolor{purple}{89.70}}$	${\textcolor{blue}{96.20}}$	${\textcolor{blue}{98.70}}$	${\textcolor{purple}{91.10}}$	${\textcolor{purple}{90.40}}$	${\textcolor{purple}{90.60}}$	${\textcolor{blue}{96.00}}$	${\textcolor{purple}{89.50}}$	${\textcolor{purple}{87.90}}$	${\textcolor{purple}{90.40}}$	${\textcolor{blue}{96.60}}$
		Bias(*× *100)	${\textcolor{red}{4.269}}$	${\textcolor{red}{4.285}}$	${\textcolor{red}{10.576}}$	${\textcolor{red}{13.920}}$	${\textcolor{purple}{0.832}}$	${\textcolor{blue}{0.472}}$	${\textcolor{red}{4.284}}$	${\textcolor{blue}{0.407}}$	${\textcolor{red}{4.285}}$	${\textcolor{blue}{\textbf{-0.069}}}$	${\textcolor{blue}{0.416}}$	${\textcolor{blue}{0.325}}$	${\textcolor{purple}{2.148}}$	${\textcolor{purple}{2.589}}$
	12	RMSE	${\textcolor{purple}{0.057}}$	${\textcolor{purple}{0.058}}$	${\textcolor{purple}{0.138}}$	${\textcolor{red}{3.012}}$	${\textcolor{blue}{0.028}}$	${\textcolor{purple}{0.051}}$	${\textcolor{purple}{0.057}}$	${\textcolor{blue}{\textbf{0.021}}}$	${\textcolor{purple}{0.058}}$	${\textcolor{blue}{0.021}}$	${\textcolor{blue}{0.022}}$	${\textcolor{blue}{0.021}}$	${\textcolor{purple}{0.070}}$	${\textcolor{purple}{0.061}}$
		Coverage(%)	${\textcolor{purple}{78.40}}$	${\textcolor{purple}{78.80}}$	${\textcolor{purple}{70.20}}$	${\textcolor{purple}{79.70}}$	${\textcolor{purple}{91.00}}$	${\textcolor{blue}{96.80}}$	${\textcolor{purple}{80.20}}$	${\textcolor{purple}{84.20}}$	${\textcolor{purple}{78.80}}$	${\textcolor{blue}{93.80}}$	${\textcolor{purple}{90.10}}$	${\textcolor{purple}{83.10}}$	${\textcolor{purple}{83.90}}$	${\textcolor{blue}{94.80}}$

We further considered a scenario with only one invalid IV (Supplementary Table S9). To examine the influence of the violation strength, we varied the magnitude of the pleiotropic effect through $\tau _{Y}$. In this setting, $\mathsf{cML}$ and $\mathsf{MBE}$ performed the best overall, followed by $\mathsf{Raps}$, $\mathsf{Median}$, and $\mathsf{dmIVW}_{\mathsf{MA}}$. As for $\mathsf{dmIVW}$, its performance ranked just behind these methods, mainly due to undercoverage. These results suggest that our proposed methods still provide competitive and stable performance in settings with very sparse invalid IVs.

### Incorporating weak IVs provides evidence for the protective effect of HDL-C on CAD

We investigated the effect of HDL-C on CAD using a dataset consisting of $\tilde{p}=151$ single-nucleotide polymorphisms (SNPs), and collected through a three-sample summary-data MR design [12, 26]. The analysis of this study was originally conducted by $\mathsf{Path}$ [12]. In their study, they selected *p=31* SNPs that exhibited significant associations with the exposure in the selection GWAS dataset, specifically those with $p^{\mathrm{sel}}_{\mathrm{value},j} < \alpha ^{\mathrm{sel}} = 5 \times 10^{-8}$. Using the selected SNPs, $\mathsf{Path}$ concluded that HDL-C has both protective and harmful effects on CAD through two distinct pathways. We applied $\mathsf{dmIVW}$ to the same 31 SNPs using $K_{\max }=3$. Among the 20 candidate submodels (see Fig. 1C), we selected the one-group submodel considering balanced pleiotropy ($\mathcal{M}_{2}$: *K=1*, fix $s_{1}=0$ and free $\rho _{1}$) using the gBIC criterion. The estimated log odds ratio (logOR) and its 95% confidence interval were *-0.06~(-0.214,0.096)*. Clearly, the result differed from that of $\mathsf{Path}$.

We then evaluated the sensitivity of $\mathsf{dmIVW}$ and $\mathsf{Path}$ to the tuning parameter $\alpha ^{\mathrm{sel}}$ by varying it to retain between 20% and 100% of the SNPs. First, we set the number of groups to two in our method. For $\mathsf{dmIVW}$, we took the most flexible submodel when *K=2*, namely $\mathcal{M}_{4 K_{\max }+4}$ as shown in Fig. 1C, with all parameters estimated from the data. $\mathsf{dmIVW}$ consistently estimated one positive and one negative effect for the two groups (see Fig. 4A). $\mathsf{Path}$ yielded estimates with the opposite signs for the two groups only when retaining 20% of the SNPs (31 SNPs). When using other thresholds, $\mathsf{Path}$ estimated negative effects for both groups. Second, we investigated the stability of model selection. $\mathsf{dmIVW}$ robustly identified the one-group submodel with balanced pleiotropy, whereas $\mathsf{Path}$ selected *K=3* at 20%, 50%, and 60% thresholds but shifted to *K=1* otherwise (see Fig. 4B). In summary, the results from $\mathsf{Path}$ varied markedly depending on the selection thresholds, which should be viewed with caution. By contrast, $\mathsf{dmIVW}$ performed reliably and attributed the opposite effects to balanced pleiotropy. Additional simulations based on this real data analysis suggest that the possibility of multiple pathways cannot be completely ruled out and that larger sample sizes may help further clarify the underlying structure (Supplementary Section S6.3). The estimated logORs and their confidence intervals varied smoothly with the proportion of retained SNPs (see Fig. 4C and Supplementary Table S11). The robustness of $\mathsf{dmIVW}$ was further confirmed in the two-sample MR design, where reusing the exposure set as the selection set still produced similar results (see Supplementary Fig. S10). When the full SNP set was included, $\mathsf{dmIVW}$ detected a protective effect of HDL-C on CAD, with the logOR of −0.129 (−0.241, −0.017). This was because weak IVs provided valuable information, and in some cases, they needed to be retained to enhance statistical power. This demonstrates the ability of $\mathsf{dmIVW}$ to handle weak IVs.

### BMI is a risk factor for AIS and CAD and increases SBP

Over the past 30 years, the global prevalence of weight-related conditions has risen, mainly driven by overweight and obesity [38]. Investigating the health impact of BMI, a key indicator of body weight status, is essential for developing effective prevention and intervention policies. In the second application, we analyzed the effect of BMI on atherosclerotic ischemic stroke (AIS), coronary artery disease (CAD), and systolic blood pressure (SBP) under the three-sample MR design [26–28]. An additional simulation based on the BMI–CAD dataset verified that the residual linkage disequilibrium (LD) after pruning is modest (see Supplementary Section S6.2). We also examined the effect of BMI on itself, serving as a validation dataset with the true causal effect known to be one.

We compared the MR methods considered in our individual data simulation. We systematically varied the P-value thresholds $\alpha ^{\mathrm{sel}}\in \{10^{-9}, 10^{-7}, 10^{-5}, 10^{-3}, 10^{-1}, 1\}$ to enhance the reliability across different threshold values. For clarity, full results, including those from methods that exhibited variable results ($\mathsf{MIX}$, $\mathsf{MBE}$), failed to output results in certain cases ($\mathsf{cML}$), or yielded results similar to others ($\mathsf{ConMIX}$), are provided in Supplementary Tables S12 and S13. The methods could be classified into two types based on the results associated with the BMI–BMI dataset: those that ignored ($\mathsf{IVW}$, $\mathsf{Egger}$, $\mathsf{Median}$, $\mathsf{MIXIE}$, $\mathsf{MBE}$) or accounted for measurement error inefficiently ($\mathsf{ConMIX}$, $\mathsf{MIX}$, see our one-group IV simulation), and those that efficiently accounted for them ($\mathsf{Raps}$, $\mathsf{dIVW} $, $\mathsf{Local}$, $\mathsf{dmIVW}$, $\mathsf{dmIVW}_{\mathsf{MA}}$). When weak IVs were included ($\alpha ^{\mathrm{sel}}\in \{1,10^{-1},10^{-3}\}$), the former group was prone to attenuation bias, with estimates biased toward zero. In contrast, the latter group effectively adjusted for measurement error, and their confidence intervals included the true causal effect—one. Our two-group IV simulation was designed to provide a more comprehensive comparison of the methods in the latter group, in which $\mathsf{dmIVW}$ and $\mathsf{dmIVW}_{\mathsf{MA}}$ demonstrated superior robustness under pleiotropy.

We next examined the effect of BMI on AIS, CAD, and SBP. When all SNPs were included, most methods concluded that BMI was a risk factor for AIS and CAD, and that it increased SBP (see Fig. 5). An epigenome-wide association study suggests that BMI may contribute to asthma and cardiovascular diseases through DNA methylation [39]. Additionally, evidence from randomized controlled trials indicates that weight reduction can lower blood pressure, and prospective studies and clinical trials further suggest that drug treatment for hypertension reduces the risk of cardiovascular diseases [40]. However, the statistical significance of the detected relationship decreased as fewer IVs were included, which aligned with our analysis of the effect of HDL-C on CAD and previous studies [26]. Regarding the stability of the results to the selection threshold, our estimated effects and confidence intervals changed smoothly as $\alpha ^{\mathrm{sel}}$ varied. An interesting comparison could be made between $\mathsf{dIVW} $ and its robust versions—$\mathsf{Local}$, $\mathsf{dmIVW}$, and $\mathsf{dmIVW}_{\mathsf{MA}}$ in the BMI-SBP analysis. Both $\mathsf{dIVW}$ and $\mathsf{Local}$ exhibited a slight discontinuity in their estimates at $\alpha ^{\mathrm{sel}}=10^{-5}$. This threshold corresponded to the smallest standard error of the estimated causal effect, and thus, it was considered optimal according to the empirical oracle criterion [28]. In contrast, $\mathsf{dmIVW}$ and $\mathsf{dmIVW}_{\mathsf{MA}}$ provided similar estimates using thresholds *1* and $10^{-3}$. This was likely due to the heterogeneity of the SNPs, which was captured by the two-group model ($\mathcal{M}_{6}$: *K=2*, fix $s_{1}=0$, $\gamma _{1}=\gamma _{2}$, free $\rho _{1}$) selected based on the gBIC criterion. Furthermore, we quantified the sensitivity to the selection threshold as the sum of adjacent absolute differences (SAAD, see Fig. 5B), which measures the cumulative absolute differences between consecutive causal effect estimates obtained using adjacent thresholds. Both $\mathsf{dmIVW}$ and $\mathsf{dmIVW}_{\mathsf{MA}}$ showed considerably smaller SAAD values, with the smallest SAAD observed in the BMI-AIS and BMI-SBP analyses. The robustness to variations in the P-value selection threshold enhanced the reliability of our methods.

### BMI and weight are two major health risk factors, while years of schooling is a protective factor

In the third application, we used MR methods to identify risk factors for diseases based on a dataset [41, 42] of eight exposures and thirty diseases, employing the three-sample MR design. We excluded $\mathsf{MIXIE}$ due to its erroneous output of zero P-values in some cases, and $\mathsf{ConMIX}$ because it failed to produce results within three days in several instances. Using the selection threshold $\alpha ^{\mathrm{sel}}=10^{-5}$, several causal relationships were consistently detected by $\mathsf{IVW}$, $\mathsf{dIVW}$, $\mathsf{cML}$, $\mathsf{Local}$, $\mathsf{dmIVW}$, $\mathsf{Raps}$, and $\mathsf{dmIVW}_{\mathsf{MA}}$ at the 0.05 significance level (see Fig. 6): (i) BMI and weight increased the risk of ischemic stroke (IschStroke), cataract, congestive heart failure (CongestHF), atrial fibrillation (AtrialFib), and arrhythmia; (ii) BMI increased the risk of type 2 diabetes (Type2Diab) and coronary artery disease (CoronaryAD), while decreasing the risk of periodontal disease (PerioDis); (iii) weight decreased the risk of cerebral aneurysm (CerebAneur) and drug eruption (DrugErupt); (iv) height increased the risk of atrial fibrillation and arrhythmia; (v) years of schooling lowered the risk of chronic hepatitis C (Chronic Hep C), cardiovascular diseases, cataracts, and arrhythmia. For the remaining methods, $\mathsf{Egger}$, $\mathsf{MBE}$, $\mathsf{Median}$, and $\mathsf{MIX}$ appeared to be conservative, as they failed to detect weight, height, and years of schooling as significant risk or protective factors. These conservative tendencies cast doubt on the validity of the identified causal relationships. However, our method and $\mathsf{cML}$, as robust approaches, provided strong evidence supporting the credibility of these causal links. Our method primarily selected one-group submodels based on the gBIC criterion for most of the identified causal relationships. Notably, the one-group model with dispersion ($\mathcal{M}_{2}$) was selected in both current and previous BMI–CoronaryAD analyses using different datasets, with estimated effects ranging from 0.3 to 0.42 and still significant across all thresholds. Two exceptions selected submodels with uncorrelated pleiotropy: the association between type 2 diabetes and BMI ($\mathcal{M}_{6}$: *K=2*, fix $s_{1}=0$, $\gamma _{1}=\gamma _{2}$, free $\rho _{1}, s_{2}, \rho _{2}$) and years of schooling ($\mathcal{M}_{5}$: *K=2*, fix $s_{1}=0$, $\rho _{1}=1$, $\gamma _{1}=\gamma _{2}$, free $s_{2}, \rho _{2}$). A simulation study based on this real dataset shows that $\mathsf{dmIVW}$ correctly identifies the true submodel with high frequency (see Supplementary Section S6.4). Our previous applications suggested that introducing more weak IVs could help improve the power of the tests. By increasing the threshold to $10^{-3}$ and $10^{-4}$, our method generally provided consistent results (Supplementary Figs S11 and S12) with the protective effect of years of schooling becoming more pronounced. In contrast, $\mathsf{cML}$ additionally identified numerous relationships. Examples included left ventricular ejection fraction being protective against Type2Diab and CoronaryAD but detrimental to Osteoporosis, open-angle glaucoma (OpenAngleGlau), chronic hepatitis B (Chronic Hep B), and atopic dermatitis (AtopicDerm), which lacked strong supporting evidence.

## Discussions

In this paper, we develop a general framework that can robustly and efficiently estimate causal effects in the presence of weak IVs and pleiotropy. It builds upon a broad range of mixture models that classify IVs into distinct groups, which may consist of valid IVs or IVs exhibiting different types of pleiotropy. A key advantage is that, with suitable submodels, IVs with uncorrelated pleiotropy can be adjusted to improve detection efficiency, while IVs with correlated pleiotropy are excluded to preserve robustness. For each submodel, estimation follows a two-stage procedure. The first stage estimates the degree of balanced pleiotropy and group composition, while the second stage accounts for directional pleiotropy and corrects measurement error by debiasing the estimating equation. Due to the unified framework, we can use the gBIC to comprehensively select the best submodel (denoted as $\mathsf{dmIVW}$) or average different submodels (denoted as $\mathsf{dmIVW}_{\mathsf{MA}}$). Both estimators exhibit comparable performance in our extensive simulations and real-world data applications, though each has distinct advantages. Specifically, $\mathsf{dmIVW}_{\mathsf{MA}}$ incorporates the submodel selection effect into the standard error estimation, resulting in adequate coverage in our individual data simulation (Table 1). In contrast, $\mathsf{dmIVW}$ is more interpretable, as it explicitly identifies the selected submodel, thereby providing insight into the type of pleiotropy.

In real-world applications, information about the type of pleiotropy is limited before analyzing data. Methods [13, 19] designed to handle correlated pleiotropy fail to fully exploit the information provided by IVs with uncorrelated pleiotropy. In fact, our two-group IV simulation study demonstrates that the information from uncorrelated pleiotropy can significantly boost detection power. The concept of exploiting IVs with uncorrelated pleiotropy is proposed by $\mathsf{MIXIE}$ [11], which integrates $\mathsf{IVW}$ for valid IVs and $\mathsf{Egger}$ for uncorrelated pleiotropy. However, its strategy of handling correlated pleiotropy, data perturbation [11, 20], lacks a clear and intuitive explanation. In addition, it does not account for measurement error, possibly because considering measurement error within a mixture model framework is particularly challenging, as shown in our one-group IV simulation.

To avoid selection bias and ensure reliability, we employ the three-sample MR design [26] with several selection thresholds in our real data analysis. Some methods are sensitive to the selection threshold. For instance, when studying the effect of HDL-C on CAD, $\mathsf{PATH}$ [12] produced noticeably different results in both model selection and causal effect estimation as the selection threshold varied, leading to unreliable outcomes (Figs 4A and 4B). In addition, incorporating weak IVs can sometimes reveal significant relationships between exposure and outcome with proper adjustment of measurement error. For example, our method provides evidence for the protective effect of HDL-C on CAD, the increased risk associated with higher BMI for various diseases and health conditions, and the protective role of years of schooling.

To systematically understand how other methods handle measurement error, we collect two-sample MR methods summarized in a comprehensive review [6], with a focus on those developed within an explicit frequentist framework (Supplementary Table S1). The first category requires NOME, including $\mathsf{IVW}$ [35], $\mathsf{Egger}$ [15], $\mathsf{Median}$ [16], $\mathsf{MBE}$ [17], $\mathsf{MIXIE}$ [11], $\mathsf{MRLASSO}$/$\mathsf{MRRobust}$ [43], $\mathsf{CIV}$ [44], and $\mathsf{regularization}$ [45]. The remaining methods can be classified into five types, each handling measurement error in a manner similar to one of the baseline estimators introduced in our one-group IV simulation. The $\mathsf{MLE}_{\mathsf{ratio}}$ type, including $\mathsf{ConMIX}$ [18], $\mathsf{MRClust}$ [14], and $\mathsf{GSME}$ [33], and the $\mathsf{MQLE}$ type, represented by $\mathsf{MIX}$ [23], are developed by analyzing the distributions of $\widehat{\beta }_{Y,j}/\widehat{\beta }_{M,j}$ and $\widehat{\beta }_{Y,j}-\gamma \widehat{\beta }_{M,j}$, respectively. Although both types attempt to model the measurement error in the target distribution, they do not fully adjust for it. The $\mathsf{MQLE}$ class provides a conservative estimator of the dispersion parameters. The $\mathsf{MLE}_{\mathsf{pair}}$ type, encompassing $\mathsf{cML}$ [20], $\mathsf{Path}$ [12], and $\mathsf{MRCIP}$ [34], focuses on modeling the IV-exposure and IV-outcome effect pairs $\big (\widehat{\beta }_{M,j},\widehat{\beta }_{Y,j}\big )$. These methods fail to account for the case where all IVs suffer from pleiotropy, e.g. balanced pleiotropy $\psi _{j}\sim \mathcal{N}\big (0,(\rho -1)\sigma _{Y,j}^{2}\big )$. The $\mathsf{MPLE}$ type includes $\mathsf{Raps}$ [27] and $\mathsf{GRAPPLE}$ [13], which perform similarly to $\mathsf{MLE}_{\mathsf{pair}}$ but are capable of adjusting balanced pleiotropy because their objective functions have fewer parameters. The final $\mathsf{MoE}$ class includes $\mathsf{dIVW}$ [28] and $\mathsf{Local}$ [22], which use unbiased estimating equations to adjust for measurement error and balanced pleiotropy simultaneously. Our proposed methods, $\mathsf{dmIVW}$ and $\mathsf{dmIVW}_{\mathsf{MA}}$, can be seen as a hybrid of the $\mathsf{MoE}$ and $\mathsf{MQLE}$ estimators and hence inherit the advantages of both. Specifically, they retain the conservative estimation property of the dispersion parameter and integrate it with the mixture model, as in the $\mathsf{MQLE}$ type, while also accounting for measurement error, as in the $\mathsf{MoE}$ type. Finally, since measurement error and the situation where all IVs suffer from balanced pleiotropy are among the most common and simple cases [28], we recommend that any method that formally considers both should be tested using our one-group IV simulation.

We point out a few limitations and future research directions. First, our framework assumes SNP independence, which can be achieved by performing stringent LD pruning using PLINK software [46]. When focusing on a single genomic region, the *cis*-SNPs are typically correlated [21] and the LD may induce pleiotropy. A two-stage framework [47] could address this by first adjusting for LD-induced pleiotropy using conditional analysis [48] and then performing MR analysis. Second, our approach currently considers only one exposure and one outcome at a time. However, real-world scenarios often require simultaneous consideration of multiple exposures to capture complex relationships. Extending $\mathsf{dmIVW}$ to multivariate MR frameworks [49] would improve its applicability. A spectral regularized estimator is proposed to generalize $\mathsf{dIVW}$ (a special case of $\mathsf{dmIVW}$) to multivariable MR [29], which provides a promising direction for extending our method. Finally, the model selection performance of $\mathsf{dmIVW}$ in distinguishing different pleiotropic structures has so far been assessed mainly through simulation studies, including semi-synthetic simulations that preserve the empirical distribution of SNP effects in real datasets (Supplementary Sections S6.3 and S6.4). However, the pleiotropic structures identified in real-data applications still require further biological validation.

In conclusion, we propose a flexible MR framework that simultaneously achieves robustness and efficiency. We demonstrate its efficiency through simulation studies and real data analyses. Our method could serve as a useful tool for medical research and public health to discover causal relationships between the factors of interest and diseases.

## Method

### Model setup

In this section, we specify the model framework to accommodate heterogeneity in IV validity due to unmeasured confounding.

The unmeasured confounders may distort the estimation of the causal effect by influencing both exposure and outcomes. Although the genetic variants are used as IVs in MR, they may also be correlated with different confounders with different strengths. We classify the IVs into *K* groups, $\{\mathcal{J}_{k}\}$, according to the strengths of their associations with the confounders. As displayed by the causal diagram (see Fig. 1A), we can depict the relationships between the IVs, confounders, exposure, and outcome through the following structural equations


(2) \begin{align*}& \begin{aligned} & U^{k}_{i}= \sum_{j\in\mathcal{J}_{k}}\delta_{U,j} G_{ij}+\epsilon_{U,i} \,,\\ & M_{i}=\sum_{j=1}^{p} \delta_{M, j} G_{ij}+\sum_{k=1}^{K}\theta_{M,k} U^{k}_{i}+\epsilon_{M,i}\,,\\ & Y_{i}=\gamma M_{i}+\sum_{k=1}^{K}\theta_{Y,k} U_{i}^{k}+\epsilon_{Y,i}\,, \end{aligned}\end{align*}

for *k=1,⋯ ,K* and *i=1,⋯ ,n*, where $U^{k}_{i}$, $M_{i}$, $Y_{i}$, $\{G_{ij}\}_{j=1}^{p}$ represent *k*th shared heritable factor, exposure, outcome, and genetic variants, respectively, $\delta _{M,j}$ and $\delta _{U,j}$ represent the direct effect of the *j*th IV on exposure and the unmeasured confounder $U_{k}$, and $\theta _{M,k}$ and $\theta _{Y,k}$ denote the effects of the *k*th factor on exposure and outcomes. We are interested in estimating and conducting statistical inference on *γ *, the causal effect of the exposure on the outcome. As the confounders are unobserved, we regress the exposure and outcome on the IVs. The associations between the *j*th IV and exposure and outcome, $\widehat{\beta }_{M,j}$ and $\widehat{\beta }_{Y,j}$, estimated from two nonoverlapping GWAS datasets independently follow normal distributions $\mathcal{N}(\beta _{M,j},\sigma _{M,j}^{2})$ and $\mathcal{N}(\beta _{Y,j},\sigma _{Y,j}^{2})$. It should be noted that when the outcome is binary, the third equation in (2) is described by a logit relationship. In this case, MR employs logistic regression to estimate the IV–outcome associations, and the causal effect is consistently estimated as the log odds ratio, up to a scaling constant determined by the residual variance [13, 27].

According to model (2), the underlying true associations of the genetic variants with the exposure and the outcome follow


(3) \begin{align*}& \beta_{M,j} = {\delta}_{M,j} + \theta_{M,k} {\delta}_{U,j}, \quad \beta_{Y,j} = \gamma \left({\delta}_{M,j} + \theta_{M,k} {\delta}_{U,j}\right) + \theta_{Y,k} {\delta}_{U,j},\end{align*}

for the *j*th IV belonging to the *k*th group. Here, $\theta _{Y,k}\delta _{U,j}$ is the pleiotropy term. When it is zero, then $\beta _{M,j}$ and $\beta _{Y,j}$ strictly satisfy the linear relationship $\beta _{Y,j}=\gamma \beta _{M,j}$. To further analyze the distortion introduced by pleiotropy, we assume that $\delta _{M,j}\stackrel{i.i.d.}{\sim }\mathcal{N}\big (\mu _{\delta _{M}},\sigma ^{2}_{\delta _{M}}\big )$ and $\delta _{U,j}\stackrel{i.i.d.}{\sim }\mathcal{N}\big (\mu _{\delta _{U_{k}}},\sigma _{\delta _{U_{k}}}^{2}\big )$ for $j\in \mathcal{J}_{k}$, and they are independently generated. The parameters *γ *, $\theta _{M,k}$, and $\theta _{Y,k}$ are fixed. Then, $\beta _{M,j}$ and pleiotropy $\theta _{Y,k}\delta _{U,j}$ follow the bivariate normal distribution. We can decompose the pleiotropic effect into two components: one completely determined by $\beta _{M,j}$, and another that is orthogonal to $\beta _{M,j}$. Specifically, $\theta _{Y,k} \delta _{U,j}=\theta _{Y,k}c_{k}\beta _{M,j} +\theta _{Y,k} (\delta _{U,j}-c_{k}\beta _{M,j})$, where $c_{k}={\theta _{M,k}\sigma _{\delta _{U_{k}}}^{2}}/(\sigma _{\delta _{M}}^{2}+\theta _{M,k}^{2}\sigma _{\delta _{U_{k}}}^{2})$ is derived in Supplementary Section S3.1. Rearranging (3), we notice that $\beta _{M,j}$ and $\beta _{Y,j}$ are related through the following equation:


\begin{align*} &\beta_{Y,j}=(\gamma+\theta_{Y,k}c_k)\beta_{M,j}+\theta_{Y,k} (\delta_{U,j}-c_k\beta_{M,j}).\end{align*}

Our primary goal is to estimate the true causal effect, *γ *, but the presence of numerous nuisance parameters in the model poses a challenge to accurately estimating the true causal effect. To address this, we adopt a working model to eliminate these nuisance terms. Specifically, we define $\gamma _{k} = \gamma + \theta _{Y,k}c_{k}$ and let $\psi _{j} = \theta _{Y,k}(\delta _{U,j} - c_{k}\beta _{M,j})$ for $j\in \mathcal{J}_{k}$. This allows us to classify IVs based on their pleiotropic effects, as illustrated in the causal diagram (see Fig. 1B) and our working model (see Fig. 1C). The coefficient $c_{k}$ is obtained by projecting $\delta _{U,j}$ onto $\beta _{M,j}$ so that the residual component $\delta _{U,j}-c_{k}\beta _{M,j}$ is orthogonal to $\beta _{M,j}$. This decomposition separates pleiotropic effects into a component correlated with $\beta _{M,j}$ and a residual component, which leads to the deviation and dispersion parameters $s_{k}$ and $\rho _{k}$ used in the working model. The derivations for $c_{k}$, $\rho _{k}$, and $s_{k}$ are detailed in Supplementary Section S3. Within this framework, an IV group can be:

- **Valid:** no pleiotropic effect ($\theta _{Y,k} = 0$, which implies $\gamma _{k} = \gamma $ and $\psi _{j} = 0$).
- **Uncorrelated pleiotropy:** pleiotropy is present, but independent of the IV-exposure association ($\theta _{Y,k} \neq 0, \theta _{M,k} = 0$, which implies $\gamma _{k} = \gamma $ but $\psi _{j} \neq 0$).
- **Correlated pleiotropy:** pleiotropy is present and correlated with the IV-exposure association ($\theta _{Y,k} \neq 0, \theta _{M,k} \neq 0$, which implies $\gamma _{k} \not = \gamma $ and $\psi _{j} \neq 0$).

Finally, let the first $k_{0}$ groups possess uncorrelated pleiotropy and the last $K-k_{0}$ groups exhibit correlated pleiotropy, i.e.


(4) \begin{align*}& \gamma_{k}=\gamma_{0} \quad\mathrm{for}\quad 1\leq k\leq k_{0} \quad\mathrm{and}\quad \gamma_{k}\not =\gamma_{0} \quad\mathrm{for}\quad k_{0}+1\leq k\leq K,\end{align*}

where $\gamma _{0}$ is the true causal effect of exposure on the outcome and $\gamma _{k}\not =\gamma _{0}$ because the effects estimated from the groups with correlated pleiotropy differ from the true causal effect. Furthermore, pleiotropy can be categorized as balanced pleiotropy if $\psi _{j}$ is centered around zero, or as directional pleiotropy otherwise. In the working model, we can fix $s_{k}=0$ to cover cases where the *k*th group does not have directional pleiotropy and $\rho _{k}=1$ if the *k*th group is assumed not to have balanced pleiotropy.

### The oracle debiased mixture IVW estimator

Given the group indices $\{\mathcal{J}_{k}\}$ and dispersion parameters $\{\rho _{k}\}$, we estimate $\{\gamma _{k}\}_{k=1}^{K}$ and $\{s_{k}\}_{k=1}^{K}$ using the debiased estimating equation [50, 51]. We derive the score equations of $\big \{\widehat{\beta }_{Y,j}\big \}$ under the assumption of no measurement error and then adjust for the measurement error by replacing $\widehat{\beta }_{M,j}^{2}$ with $\widehat{\beta }_{M,j}^{2}-\sigma _{M,j}^{2}$ (Supplementary Section S1). The resulting estimating equations


(5) \begin{align*} & \sum_{j=1}^{p}\frac{\boldsymbol{1}(j\in\mathcal{J}_{k})}{\rho_{k}\sigma_{Y,j}^{2}}\widehat{\beta}_{Y,j} = s_{k}\sum_{j=1}^{p} \frac{\boldsymbol{1}(j\in\mathcal{J}_{k})}{\rho_{k}\sigma_{Y,j}^{2}} +\! \gamma_{k}\sum_{j=1}^{p}\frac{\boldsymbol{1}(j\in\mathcal{J}_{k})}{\rho_{k}\sigma_{Y,j}^{2}}\widehat{\beta}_{M,j} \ \mathrm{for}\ 1\!\leq k\!\leq K, \nonumber\\ & \sum_{j=1}^{p} \frac{\boldsymbol{1}(j\in\mathcal{J}_{k})}{\rho_{k}\sigma_{Y,j}^{2}} \widehat{\beta}_{Y,j}\widehat{\beta}_{M,j} = s_{k}\sum_{j=1}^{p} \frac{\boldsymbol{1}(j\in\mathcal{J}_{k})}{\rho_{k}\sigma_{Y,j}^{2}} \widehat{\beta}_{M,j} \nonumber \\ &+ \gamma_{k}\sum_{j=1}^{p} \frac{\boldsymbol{1}(j\in\mathcal{J}_{k})}{\rho_{k}\sigma_{Y,j}^{2}} (\widehat{\beta}_{M,j}^{2}-\sigma_{M,j}^{2}) \ \mathrm{for}\ k_{0}+1\leq k\leq K, \nonumber\\ & \sum_{k=1}^{k_{0}}\sum_{j=1}^{p} \frac{\boldsymbol{1}(j\in\mathcal{J}_{k})}{\rho_{k}\sigma_{Y,j}^{2}} \widehat{\beta}_{Y,j}\widehat{\beta}_{M,j} = \sum_{k=1}^{k_{0}}s_{k}\sum_{j=1}^{p} \frac{\boldsymbol{1}(j\in\mathcal{J}_{k})}{\rho_{k}\sigma_{Y,j}^{2}} \widehat{\beta}_{M,j} \nonumber \\ &+ \gamma_{0}\sum_{k=1}^{k_{0}}\sum_{j=1}^{p} \frac{\boldsymbol{1}(j\in\mathcal{J}_{k})}{\rho_{k}\sigma_{Y,j}^{2}} (\widehat{\beta}_{M,j}^{2}-\sigma_{M,j}^{2}),\end{align*}

are unbiased (as verified by taking expectations on both sides). The obtained estimator is equivalent to some existing MR estimators by appropriately fixing parameters and applying necessary modifications. When $\widehat{\beta }_{M,j}^{2} - \sigma _{M,j}^{2}$ is replaced with $\widehat{\beta }_{M,j}^{2}$, the estimator simplifies to the $\mathsf{IVW}$ [35] estimator if *K = 1*, $\rho _{1} = 1$, and $s_{1} = 0$; to $\mathsf{Egger}$ [15] if *K = 1* and $\rho _{1} = 1$; and to $\mathsf{MIXIE}$ [11] if $K = k_{0} = 2$, $\rho _{1} = 1$, and $s_{1} = 0$. In addition, our estimator becomes the $\mathsf{dIVW}$ [28] estimator if *K=1* and $s_{1}=0$. Thus, our method is a unified framework that recovers different existing methods and enables comparisons between them.

### The feasible debiased mixture IVW estimator

As $\{\mathcal{J}_{k}\}$ and $\{\rho _{k}\}$ are unknown in practice, we take a two-stage estimation approach (see Fig. 1D). The first stage obtains mixture weights $\widehat{w}_{j,k} \in [0, 1]$ to replace $\boldsymbol{1}(j \in \mathcal{J}_{k})$ and estimated dispersion parameters $\{\widehat{\rho }_{k}\}$ using an EM-type algorithm [52, 53]. Denote the group to which the *j*th IV belongs as $U_{j}$, where $U_{j}=k$ if $j\in \mathcal{J}_{k}$, and assume that $U_{j}$ follows a multinomial distribution, i.e. $U_{j}\sim \mathrm{Multinomial}(\pi _{1},\cdots ,\pi _{K})$ with $\sum _{k=1}^{K}\pi _{k} = 1$. Conditional on $U_{j}=k$, the residuals $R_{j}=\widehat{\beta }_{Y,j}-\gamma _{U_{j}}\widehat{\beta }_{M,j}$ follow the normal distribution $\mathcal{N}\big (s_{k},\rho _{k}\sigma _{Y,j}^{2}+\gamma _{k}^{2}\sigma _{M,j}^{2}\big )$. The above discussions lead us to estimate the unknown parameters $\boldsymbol \theta =\big (\{\pi _{k}\},\{\rho _{k}\},\{s_{k}\},\{\gamma _{k}\}\big )$ and weights using an EM-type algorithm [32], which iterates an E-step and an M-step until it converges. In particular, let $\boldsymbol \theta ^{(t)}=\big (\{\pi _{k}^{(t)}\},\{\rho _{k}^{(t)}\},\{s_{k}^{(t)}\},\{\gamma _{k}^{(t)}\}\big )$ and $\{w_{jk}^{(t)}\}$ be the *t*th iteration of the parameters and weights updated from the EM algorithm. The corresponding E-step and M-step are summarized as follows.

1. E-step: calculate $B \left (\boldsymbol \theta ;\boldsymbol \theta ^{(t)}\right )=\sum _{j=1}^{p}\sum _{k=1}^{K}\Big [ w_{jk}^{(t)} \log \Big \{{\pi _{k} f_{jk}(\widehat{\beta }_{Y,j},}{\widehat{\beta }_{M,j};\boldsymbol \theta )}\Big \}-w_{jk}^{(t)}\log (w_{jk}^{(t)})\Big ]$, where $ w_{jk}^{(t)} \propto{\pi ^{(t)}_{k} f_{jk}(\widehat{\beta }_{Y,j},\widehat{\beta }_{M,j};\boldsymbol \theta ^{(t)})}$ with $\sum _{k=1}^{K}w_{jk}^{(t)}=1$ and $f_{jk}(\widehat{\beta }_{Y,j},\widehat{\beta }_{M,j};\boldsymbol \theta )$ denotes the normal density of $R_{j}=\widehat{\beta }_{Y,j}-\gamma _{k}\widehat{\beta }_{M,j}$ with mean $s_{k}$ and variance $\rho _{k}\sigma _{Y,j}^{2}+\gamma _{k}^{2}\sigma _{M,j}^{2}$.
2. M-step: obtain $\boldsymbol \theta ^{(t+1)}=\arg \max _{\boldsymbol \theta }B(\boldsymbol \theta ;\boldsymbol \theta ^{(t)})$.

Intuitively speaking, the E-step maximizes optimal lower bound $B\left (\boldsymbol{\theta }^{(t)};\boldsymbol{\theta }^{(t)}\right )$ by adjusting weights $w_{jk}^{(t)}$ (see Supplementary Section S2 for details). We initialize the algorithm from 20 random starting points to improve numerical stability and impose the constraint $\rho _{k} \ge 1$ to avoid degenerate variance estimates. Denote $n_{\mathrm{para.}}$ as the number of parameters and $\widehat{\boldsymbol{\theta }} = \big (\{\widehat{\pi }_{k}\},\{\widehat{\rho }_{k}\},\{\widehat{s}_{k}\},\{\widehat{\gamma }_{k}\}\big )$ as the output estimates. The gBIC [12, 31] for latent variable models is defined as


\begin{align*} &\mathrm{gBIC}(\mathcal{M}) = -2 B(\widehat{\boldsymbol{\theta}};\widehat{\boldsymbol{\theta}}) + n_{\mathrm{para.}}\log(p) \,,\end{align*}

which can be used to select and average submodels.

The second stage calculates the final estimator $\{\widehat{\gamma }_{k}\}$ and $\{\widehat{s}_{k}\}$ by solving (5) using weights $\{\widehat{w}_{j,k}\}$ and dispersion parameters $\{\widehat{\rho }_{k}\}$ estimated from the first stage.

We use a parametric bootstrap approach [54–56] to estimate the standard errors of $\widehat{\boldsymbol{\theta }}$. Specifically, we perform *B=200* bootstrap iterations, which is sufficient to provide stable uncertainty estimates (Supplementary Table S7). In each iteration * b = 1, ⋯ , B *, we begin by generating the latent group indicator $ u^{(b)}_{j} $ based on the estimated weights $ \{\widehat{w}_{j,k}\} $. Next, we compute adjusted values for the coefficients $ \beta ^{(b)}_{M,j} $ and $ \beta ^{(b)}_{Y,j}$ through $ \beta ^{(b)}_{M,j} = \mathrm{sign}(\widehat{\beta }_{M,j}) \{\max (0, \widehat{\beta }_{M,j}^{2} - \sigma _{M,j}^{2})\}^{1/2} $ and $ \beta ^{(b)}_{Y,j} = \widehat{\gamma }_{u^{(b)}_{j}} \beta ^{(b)}_{M,j} + \widehat{s}_{u^{(b)}_{j}} $. We adopt truncation to improve numerical stability because negative values arise from finite-sample variability. Subsequently, the bootstrap values $ \widehat{\beta }_{M,j}^{(b)} $ and $ \widehat{\beta }_{Y,j}^{(b)} $ are generated from independent normal distributions. Using the generated sample, we calculate the estimated parameters $\widehat{\boldsymbol{\theta }}^{(b)}$. After completing all *B* iterations, we estimate the standard errors of $\, \widehat{\boldsymbol{\theta }}$ based on the bootstrap samples and construct the confidence interval using the normal approximation.

### Identify the causal effect

Our framework allows $K\times 2^{2K}$ submodels for any *K>0*, determined by whether $s_{k}$ is fixed at zero, $\rho _{k}$ is fixed at one, and the value of $k_{0}$. In practice, we consider a more meaningful set of submodels. First, we consider $k_{0} = K$ for uncorrelated pleiotropy and $k_{0} = 1$ for correlated pleiotropy. Second, only the first group is allowed to possibly consist of valid IVs (referred to as the causal group), with $s_{1}$ and $\rho _{1}$ either fixed or estimated, while for *k ≥ 2*, $s_{k}$ and $\rho _{k}$ are always obtained through estimation. Given that *K* ranges from 1 to $K_{\max }$ there are $4 + 8(K_{\max } - 1)$ submodels (see Fig. 1C).

*Identifiability in population level.* For submodels considering uncorrelated pleiotropy ($k_{0} = K$), the true causal effect is shared across all IV groups and thus identifiable. For submodels considering correlated pleiotropy ($k_{0} = 1$), the causal group needs to be determined among multiple groups. Failure to correctly identify the causal group may lead to a biased causal effect estimate. Intuitively, it should be the one with the largest proportion or the least degree of (either balanced or directional) pleiotropy. Suppose that groups are ordered by proportion, followed by dispersion level and deviation magnitude, to select the causal group. Then, the causal group is identifiable if one of the following conditions hold:

(a) For all $2 \le k \le K$,
  (6) \begin{align*}& \begin{aligned} & \bigl|\{j \in \mathcal{J}_{1}: \gamma_{1} \beta_{M,j} + s_{1} \neq \gamma_{k} \beta_{M,j} + s_{k} \ \mathrm{or}\ \rho_{1} \neq \rho_{k}\}\bigr| \\ & \quad > |\mathcal{J}_{k}| + \sum_{l \in \{2,\dots,K\}\setminus\{k\}} \bigl|\{j \in \mathcal{J}_{l}: \gamma_{l} \beta_{M,j} + s_{l} = \gamma_{k} \beta_{M,j} \\ &\qquad+ s_{k} \ \mathrm{and}\ \rho_{l} = \rho_{k}\}\bigr|, \end{aligned}\end{align*}
  which generalizes the plurality rule of [17] and reduces to it when $s_{k} = 0$ for all *k*. IVs are indistinguishable between two groups if the working model implies the same mean and variance under both groups, which is accounted for by the adjustment terms. A more conservative sufficient condition is the majority rule, $|\mathcal{J}_{1}|>p/2$. This ensures identifiability even when several pleiotropic groups exhibit similar pattern, a phenomenon observed in our five-group simulation.
(b) For all *k ≥ 2*, the inequality (6) relaxed to a non-strict one holds with $\rho _{1} < \rho _{k}$.
(c) For all *k ≥ 2*, the inequality (6) relaxed to a non-strict one holds with $\rho _{1} \le \rho _{k}$ and $|s_{1}| < |s_{k}|$.

*Causal group selection procedure.* The above conditions describe the population-level criteria under which the causal group is well defined. In finite samples, however, the corresponding group characteristics (e.g. proportions, dispersion, and deviation) must be estimated, and their empirical ordering may be affected by sampling variability. Take the identification of the group with the largest proportion as an example. Due to randomness, the ranking of estimated proportions may not preserve the true population ordering. To address this, we perform a rank verification test [57–59] to statistically evaluate whether the group with the highest estimated proportion aligns with the true group having the maximum proportion. We implement an unadjusted two-tailed pairwise test to compare the first two-order statistics [58] using a bivariate normal approximation based on bootstrap estimates at the 0.05 significance level. If the difference is statistically significant, the causal effect corresponding to the group with the largest $\widehat{\pi }_{k}$ is chosen as the final estimator. If not, we sequentially evaluate the degree of balanced pleiotropy using dispersion parameters $\{\widehat{\rho }_{k}\}$ and of directional pleiotropy using deviation parameters $\{\widehat{s}_{k}\}$. If none of these criteria yield a significant result, we default to selecting the group based on the largest $\{\widehat{\pi }_{k}\}$.

*Credibility of group selection results.* The group selection procedure relies on three criteria, which may suggest different causal groups. We classify the credibility of the final selection into four levels based on the consistency of the selected groups across these criteria: as High if all criteria select the same group and all tests are significant; as Moderate if they select the same group but not all tests are significant; as Weak if some criteria select groups different from the final selection but the corresponding differences are not significant; and as Questionable if some criteria significantly favor a group different from the final selection. The validity of this credibility assessment is demonstrated in the simulation of our HDL-C–CAD analysis (Supplementary Section S6.3).

### Model selection and model average

For each submodel $\mathcal{M}$, we denote the gBIC, the estimated causal effect, and its standard error as $\mathrm{gBIC}_{\mathcal{M}}$, $\widehat{\gamma }_{\mathcal{M}}$, and $\widehat{\sigma }_{\mathcal{M}}$, respectively. The *model selection* approach chooses the submodel with the lowest gBIC [12, 31]. The *model averaging* approach [11, 20, 21, 36] proceeds as follows. Let $\mathcal{M}_{1}, \mathcal{M}_{2},\cdots ,\mathcal{M}_{M}$ denote the *M* submodels with the lowest gBIC values. The model averaging estimator and its standard error are calculated as


\begin{align*} \widehat{\gamma}^{\mathrm{MA}}_0 &= \sum_{m=1}^M w_{\mathcal{M}_m}\widehat{\gamma}_{\mathcal{M}_m}\quad \mathrm{and}\quad \mathrm{SE}\left(\widehat{\gamma}^{\mathrm{MA}}_0\right) \\ &=\sum_{m=1}^M w_{\mathcal{M}_m}\sqrt{\widehat{\sigma}_{\mathcal{M}_m}^2+\left(\widehat{\gamma}^{\mathrm{MA}}_0-\widehat{\gamma}_{\mathcal{M}_m}\right)^2} \,\end{align*}

where $w_{\mathcal{M}_{m}}\propto \exp \big (-\mathrm{gBIC}_{\mathcal{M}_{m}}/2\big )$ with $\sum _{m=1}^{M} w_{\mathcal{M}_{m}}=1$ being the averaging weights.

### Simulation setups

*One-group IV simulation.* We generated synthetic data mimicking the BMI–BMI dataset in our second application, denoted as $\{(\widetilde \beta _{M,j}, \widetilde \beta _{Y,j}, \widetilde \sigma _{M,j}^{2}, \widetilde \sigma _{Y,j}^{2})\}_{j=1}^{p}$ with *p = 812* SNPs. Given the IV strength $\kappa \in \{1, \dots , 20\}$, the simulated IV-exposure effects satisfied $\beta _{M,j} \propto \widetilde \beta _{M,j}$ and $\kappa = p^{-1} \sum _{j=1}^{p} {\beta _{M,j}^{2}}/{\sigma _{M,j}^{2}}$. We set $\sigma _{M,j}^{2} = \widetilde \sigma _{M,j}^{2}$, $\sigma _{Y,j}^{2} = \widetilde \sigma _{Y,j}^{2}$, and $\beta _{Y,j} = \beta _{M,j}$ because the true effect of BMI on BMI is one. We considered two scenarios S1 and S2. Scenario S1 set *K=1*, $s_{1}=0$, and $\rho _{1}=1$ to ensure that all IVs were valid. Scenario S2 took *K=1*, $s_{1}=0$, and $\rho _{1}=5$ such that the IVs exhibited balanced pleiotropy.

*Two-group IV simulation.* We considered *K = 2* IV groups with $n = 10^{5}$ and *p = 2000*. The IV-exposure effects $\beta _{M,j}$ were generated from $\mathcal{N}\big (0, \sigma _{\beta _{M}}^{2}/p\big )$ with $\sigma _{\beta _{M}}^{2} = 0.1$. The standard deviation of the IV-exposure and IV-outcome effects, $\sigma _{M,j}$ and $\sigma _{Y,j}$, were independently sampled from $U[0.9, 1.1]/\sqrt{n}$. The causal effect $\gamma _{0}$ ranged from 0 to 0.1. The first group was the valid group, with $\gamma _{1} = \gamma _{0}$, $\rho _{1} = 1$, and $s_{1}= 0$, and the probability of an IV belonging to this group was $\pi _{\mathrm{valid}}$. In Setups 1 and 2, the IVs in the second group exhibited correlated pleiotropy, where $\gamma _{2} = \gamma _{0} - 2.5$, providing no useful information about the causal effect. Setup 1 set $\pi _{\mathrm{valid}} = 0.8$, $\rho _{2} = 1$, and $s_{2} = 0$ to identify $\gamma _{0}$ based on the proportions of groups. Setup 2 set $\pi _{\mathrm{valid}} = 0.5$, $\rho _{2} = 5$, and $s_{2} = 0$, aiming to identify $\gamma _{0}$ through the degree of balanced pleiotropy. In Setups 3a and 3b, the second-group IVs were affected by uncorrelated directional pleiotropy with $s_{2} = 0.02$ and $\rho _{2} = 1$, while in Setups 4a and 4b, they exhibited uncorrelated balanced pleiotropy with $s_{2} = 0$ and $\rho _{2} = 5$. In Setups 3a–4b, the second group could facilitate causal effect estimation because $\gamma _{2} = \gamma _{0}$. Setups 3a and 4a were expected to yield better performance with $\pi _{\mathrm{valid}} = 0.2$ compared with Setups 3b and 4b, which used $\pi _{\mathrm{valid}} = 0.5$.

*Five-group IV simulation.* We considered a setup with *K=5* similar to the two-group IV simulation, using the same data-generating mechanism for $(\beta _{M,j}, \sigma _{M,j}, \sigma _{Y,j})$. The first group was the valid group with $\gamma _{1}=\gamma _{0}=0.05$, $\rho _{1}=1$, and $s_{1}=0$, and $\pi _{\mathrm{valid}}=0.6$. The remaining four groups were invalid groups with $\pi _{k}=0.1$ for $2\leq k\leq 5$. In Case 1, these invalid groups exhibited correlated pleiotropy with $\gamma _{2}=\gamma _{0}+1$, $\gamma _{3}=\gamma _{0}+1.5$, $\gamma _{4}=\gamma _{0}+2$, and $\gamma _{5}=\gamma _{0}+2.5$. In Case 2, they also exhibited correlated pleiotropy but with effects located on both sides of the true causal effect, with $\gamma _{2}=\gamma _{0}-2.5$, $\gamma _{3}=\gamma _{0}-1.5$, $\gamma _{4}=\gamma _{0}+1.5$, and $\gamma _{5}=\gamma _{0}+2.5$. In both cases, all groups satisfied $s_{k}=0$, with dispersion parameters $\rho _{2}=\rho _{4}=5$ and $\rho _{3}=\rho _{5}=1$. In Case 3, we considered a mixture of correlated and uncorrelated pleiotropy. The second group satisfied $\gamma _{2}=\gamma _{0}$ but exhibited uncorrelated directional pleiotropy with $s_{2}=0.02$. The remaining groups followed correlated pleiotropy with $\gamma _{3}=\gamma _{0}-2.5$, $\gamma _{4}=\gamma _{0}+1.5$, and $\gamma _{5}=\gamma _{0}+2.5$, while $s_{k}=0$. The dispersion parameters were the same as in Cases 1 and 2.

*Individual data simulation.* We conducted simulations based on the underlying genetic model [17], which is equivalent to (2) while the influence of *p* genetic variants on shared heritable factor $U_{i}$, exposure $M_{i}$, and outcome $Y_{i}$ is easier to quantify. The underlying mechanism satisfied $ U_{i} = \tau _{U} Z_{U,i}+\epsilon _{U,i}$, $M_{i} = \tau _{M}Z_{M,i}+\theta _{M} U_{i}+\epsilon _{M,i}$, and $Y_{i} = \tau _{Y}Z_{Y,i}+\theta _{Y} U_{i}+\gamma _{0} M_{i}+\epsilon _{Y,i}$, where $\epsilon _{M,i}, \epsilon _{U,i},\epsilon _{Y,i}\stackrel{i.i.d.}{\sim }\mathcal{N}(0,1)$, $Z_{M,i}=\sum _{j=1}^{p}\delta _{M,j}G_{ij}/\sigma _{Z_{M}}$, $Z_{Y,i}=\sum _{j=1}^{p}\delta _{Y,j}G_{ij}/\sigma _{Z_{Y}}$, and $Z_{U,i}=\sum _{j=1}^{p}\delta _{U,j}G_{ij}/\sigma _{Z_{U}}$ represented the additive allele scores of *p* genetic variants on exposure, outcome, and shared heritable factor, respectively, and $\sigma _{Z_{M} }$, $\sigma _{Z_{Y}}$, and $\sigma _{Z_{U}}$ were chosen to let the standard deviations of $\{Z_{M,i}\}$, $\{Z_{Y,i}\},$ and $\{Z_{U,i}\}$ to be one. Throughout, we set *p=30*, $\theta _{M}=\theta _{Y}=\sqrt{0.3}$, $\tau _{M}=\sqrt{0.1}$, $\delta _{M,j}\sim U(0.01, 0.2) $ and generated the genetic variants from binomial distribution $G_{ij}\stackrel{i.i.d.}{\sim }\mathrm{Binomial}(2,q_{j})$ with $q_{j}\sim U(0.1,0.9)$. The associated coefficients $\widehat{\beta }_{M,j}$ and $\widehat{\beta }_{Y,j}$ were obtained by regressing $M_{i}$ and $Y_{i}$, respectively, on $\{G_{ij}\}$ using two nonoverlapping datasets, each containing 10 000 individuals.

We set $\gamma _{0} = 0.1$ to evaluate power and $\gamma_0 = 0$ to evaluate Type-I error. To assess robustness in controlling Type-I error, we considered $p_{\mathrm{invalid}}$ invalid SNPs. For each valid *j*th SNP, we set $\tau _{U} = 0$, $\tau _{Y} = 0$, $\delta _{U,j} = 0$, and $\delta _{Y,j} = 0$. For uncorrelated pleiotropy, we set $\tau _{U} = 0$, $\delta _{U,j} = 0$, and $\tau _{Y} = p_{\mathrm{invalid}} \sqrt{0.1} / p$, and generated $\delta _{Y,j}$ from *U(0.01, 0.2)* for directional pleiotropy and from *U(-0.2, 0.2)* for balanced pleiotropy. For correlated pleiotropy, we set $\tau _{Y} = 0$, $\tau _{U} = p_{\mathrm{invalid}} \sqrt{0.1} / p$, and generated $\delta _{U,j}$ from *U(0.01, 0.2)* for directional pleiotropy and from *U(-0.2, 0.2)* for balanced pleiotropy.

### Baseline estimators

We introduce five baseline estimators used in the one-group IV simulation. These estimators are derived based on the following two models. Under the assumption of no pleiotropy, the associations $\widehat{\beta }_{M,j}$ and $\widehat{\beta }_{Y,j}$ independently follow normal distributions $\mathcal{N}(\beta _{M,j}, \sigma _{M,j}^{2})$ and $\mathcal{N}(\beta _{Y,j}, \sigma _{Y,j}^{2})$, respectively. Assuming IVs possess uncorrelated balanced pleiotropy, $\widehat{\beta }_{M,j}$ and $\widehat{\beta }_{Y,j}$ independently follow normal distributions $\mathcal{N}(\beta _{M,j}, \sigma _{M,j}^{2})$ and $\mathcal{N}(\beta _{Y,j}, \rho _{0} \sigma _{Y,j}^{2})$ for $\rho _{0}> 1$. For both cases, $\beta _{Y,j} = \gamma _{0} \beta _{M,j}$. The five baseline estimators based on the above model are:

-
$\mathsf{MLE}_{\mathsf{ratio}}$ : the maximum likelihood estimator for modeling the individual causal effects, defined as $\widehat{\gamma }_{j} = \widehat{\beta }_{Y,j} / \widehat{\beta }_{M,j}$. This estimator is based on the ratio of the outcome effect to the exposure effect, which reflects the causal relationship estimated through an individual SNP.
-
$\mathsf{MLE}_{\mathsf{pair}}$ : the maximum likelihood estimator for modeling the IV-exposure and IV-outcome effect pairs $\big \{ \big (\widehat{\beta }_{Y,j}, \widehat{\beta }_{M,j}\big )\big \}$.
-
$\mathsf{MPLE}$ : the estimator derived by maximizing a profiled likelihood, which eliminates the nuisance parameters $\beta _{M,j}$ in the likelihood based on $\{(\widehat{\beta }_{Y,j}, \widehat{\beta }_{M,j}) \}$.
-
$\mathsf{MQLE}$ : the estimator maximizing a quasi-likelihood that models the residuals $\{\widehat{\beta }_{Y,j} - \gamma \widehat{\beta }_{M,j}\}$.
-
$\mathsf{MoE}$ ($\mathsf{dIVW}$): the moment-based estimator, which is equivalent to the $\mathsf{dIVW}$ estimator [28].

The detailed formulas and derivations can be found in Supplementary Section S4.

**Key Points**

-
$\mathsf{dmIVW}$ is a robust Mendelian randomization method that extends **i**nverse-**v**ariance **w**eighted estimation by correcting weak-IV bias through **d**ebiasing and capturing diverse pleiotropic effects using **m**ixture models.
- It uses a two-stage estimation procedure to disentangle pleiotropy modeling from measurement-error correction.
- Candidate submodels are constructed by fixing pleiotropy-related parameters, allowing $\mathsf{dmIVW}$ to handle uncorrelated, correlated, directional, and balanced pleiotropy while recovering existing MR estimators as special cases.
- The model-selected estimator $\mathsf{dmIVW}$ and the model-averaged estimator $\mathsf{dmIVW}_{\mathsf{MA}}$ use gBIC to select and average over candidate submodels.
- Simulations demonstrate improved robustness and efficiency, and real-data analyses show stable causal estimation across instrument-selection thresholds.

## Supplementary Material

Supplementary_material_bbag468

## Data Availability

All GWAS summary datasets used in the real data analysis are publicly available at the URLs below. IEU GWAS database, https://gwas.mrcieu.ac.uk/; Genetic Investigation of ANthropometric Traits consortium, https://portals.broadinstitute.org/collaboration/giant/index.php/GIANT_consortium; Global Urate Genetics Consortium, http://metabolomics.helmholtz-muenchen.de/gugc/; Social Science Genetic Association Consortium, https://www.thessgac.org/; BioBank Japan, http://jenger.riken.jp; Neale Lab, https://www.nealelab.is/, CARDIoGRAMplusC4D Consortium, https://www.cardiogramplusc4d.org/; UK Biobank, https://www.ukbiobank.ac.uk/. The details and other relevant data are provided in Supplementary Tables S14–S19 at https://doi.org/10.6084/m9.figshare.30281053.v1. The $\mathsf{dmIVW}$ method is implemented in an open-source, publicly available R package that is available at https://github.com/denglinsui/MRdmIVW. The code to reproduce the analysis can be found at https://github.com/denglinsui/MRdmIVW-manuscript-sourcecode.
