# Supplementary material for: A flexible framework for robust and efficient Mendelian randomization with debiasing
Source: Brief Bioinform. 2026 Sep 7;27(5):bbag468. doi: 10.1093/bib/bbag468 (PMC13548358; doi:10.1093/bib/bbag468)
Supplement: Supplementary file 1 [file supplementary_material_bbag468.zip › MRdmIVWSupp_bbag468.pdf]

Supplementary Material for "A flexible framework for robust and efficient Mendelian randomization with debiasing"

This supplementary material is organized as follows. In Supplementary Section S1, we derive the debiased estimating equations in equation (5). Supplementary Section S2 presents the procedure for deriving the evidence lower bound $B(\boldsymbol{\theta}; \boldsymbol{\theta}^{(t)})$ used in the E-step. Supplementary Section S3 provides a detailed decomposition of pleiotropy into correlated and uncorrelated components and derives the associated deviation and dispersion parameters. In Supplementary Section S4, we introduce five baseline estimators used in our one-group simulation and their connections with existing MR methods. Supplementary Section S5 presents the identifiability condition of our working model. Supplementary Section S6 presents additional simulation studies and further discusses the simulations in the main paper. Supplementary Section S7 describes the supplementary tables and figures.

S1 Debiased estimating equations

In this section, we derive our debiased estimating equations with known group membership $\{\mathcal{J}_k\}$ and dispersion parameters.

We first assume that $\{\beta_{M,j}\}$ is given. According to the model in Section 2.1, we have $\widehat{\beta}_{Y,j} \sim \mathcal{N}(\gamma_k \beta_{M,j} + s_k; \rho_k \sigma_{Y,j}^2)$. The log-likelihood of $\{\gamma_k, s_k\}$ is given by

$$l(\{\gamma_k, s_k\}) = -\sum_{k=1}^{K} \sum_{j \in \mathcal{J}_k} \left\{ \frac{1}{2} \log\left(2\pi \rho_k \sigma_{Y,j}^2\right) + \frac{\left(\widehat{\beta}_{Y,j} - \gamma_k \beta_{M,j} - s_k\right)^2}{2\rho_k \sigma_{Y,j}^2} \right\}.$$

Recalling that $\gamma_k = \gamma_0$ for $1 \le k \le k_0$ in (4), and taking derivatives with respect to

$\{\gamma_0\} \cup \{\gamma_k\}_{k=k_0+1}^K \cup \{s_k\}_{k=1}^K$, we obtain the score functions

$$\sum_{j=1}^p \frac{\mathbf{1}(j \in \mathcal{J}_k)}{\rho_k \sigma_{Y,j}^2} \widehat{\beta}_{Y,j} = s_k \sum_{j=1}^p \frac{\mathbf{1}(j \in \mathcal{J}_k)}{\rho_k \sigma_{Y,j}^2} + \gamma_k \sum_{j=1}^p \frac{\mathbf{1}(j \in \mathcal{J}_k)}{\rho_k \sigma_{Y,j}^2} \beta_{M,j} \quad \text{for } 1 \le k \le K,$$

$$\sum_{j=1}^p \frac{\mathbf{1}(j \in \mathcal{J}_k)}{\rho_k \sigma_{Y,j}^2} \widehat{\beta}_{Y,j} \beta_{M,j} = s_k \sum_{j=1}^p \frac{\mathbf{1}(j \in \mathcal{J}_k)}{\rho_k \sigma_{Y,j}^2} \beta_{M,j} + \gamma_k \sum_{j=1}^p \frac{\mathbf{1}(j \in \mathcal{J}_k)}{\rho_k \sigma_{Y,j}^2} \beta_{M,j}^2 \quad \text{for } k_0 + 1 \le k \le K,$$

$$\sum_{k=1}^{k_0} \sum_{j=1}^p \frac{\mathbf{1}(j \in \mathcal{J}_k)}{\rho_k \sigma_{Y,j}^2} \widehat{\beta}_{Y,j} \beta_{M,j} = \sum_{k=1}^{k_0} s_k \sum_{j=1}^p \frac{\mathbf{1}(j \in \mathcal{J}_k)}{\rho_k \sigma_{Y,j}^2} \beta_{M,j} + \gamma_0 \sum_{k=1}^{k_0} \sum_{j=1}^p \frac{\mathbf{1}(j \in \mathcal{J}_k)}{\rho_k \sigma_{Y,j}^2} \beta_{M,j}^2 . \tag{S1}$$

It is easy to verify that the above estimating equations are unbiased, that is, the expectations of the left- and right-hand sides are equal. However, we observe $\{\widehat{\beta}_{M,j}\}$ instead of $\{\beta_{M,j}\}$. Replacing $\beta_{M,j}$ by $\widehat{\beta}_{M,j}$ in (S1) leads to many popular MR estimators in the literature. For instance, when $K = 1$, $s_1 = 0$ and $\rho_1 = 1$, we have

$$\sum_{j=1}^p \sigma_{Y,j}^{-2} \widehat{\beta}_{Y,j} \widehat{\beta}_{M,j} = \gamma_1 \sum_{j=1}^p \sigma_{Y,j}^{-2} \widehat{\beta}_{M,j}^2 \text{ and thus } \widehat{\gamma}_1 = \sum_{j=1}^p \sigma_{Y,j}^{-2} \widehat{\beta}_{Y,j} \widehat{\beta}_{M,j} / \sum_{j=1}^p \sigma_{Y,j}^{-2} \widehat{\beta}_{M,j}^2, \tag{S2}$$

which is exactly the IVW estimator in [3]. [21] analyzed and eliminated the effect of measurement error on the IVW estimator. Unfortunately, the approach of deriving the dmIVW estimator is hard to generalize to the multiple group case.

We notice that (S1) is biased if we replace $\beta_{M,j}$ by $\widehat{\beta}_{M,j}$ because $\mathbb{E}(\widehat{\beta}_{M,j}^2) = \beta_{M,j}^2 + \sigma_{M,j}^2 \neq \beta_{M,j}^2$. Therefore, we replace the quadratic terms $\beta_{M,j}^2$ by $\widehat{\beta}_{M,j}^2 - \sigma_{M,j}^2$ to eliminate the bias and replace $\beta_{M,j}$ by $\widehat{\beta}_{M,j}$. The debiased estimation equations presented in (5) are then unbiased.

This straightforward strategy offers several advantages. First, when $K = 1$ and $s_1 = 0$, the corresponding estimator is equivalent to the dIVW estimator. This provides an alternative method for deriving the dIVW estimator. Second, our estimator remains unbiased even when the IVs exhibit uncorrelated directional pleiotropy, since the estimating functions for $\{s_k\}$ are unbiased. Additionally, the debiased estimating equations presented in equation (5) are well-suited for the multiple group case due to their structure.

S2 Derivation of the evidence lower bound

Recall that the associations $\widehat{\beta}_{M,j}$ and $\widehat{\beta}_{Y,j}$ estimated from the exposure and outcome datasets independently follow normal distributions $\mathcal{N}(\beta_{M,j}, \sigma_{M,j}^2)$ and $\mathcal{N}(\beta_{Y,j}, \sigma_{Y,j}^2)$. For $j \in \mathcal{J}_k$, define $R_j = \widehat{\beta}_{Y,j} - \gamma_k \widehat{\beta}_{M,j}$. According to (1), we have

$$\{R_j \mid U_j = k\} = \left\{\widehat{\beta}_{Y,j} - \gamma_k \widehat{\beta}_{M,j} \mid U_j = k\right\} \sim \mathcal{N}\left(s_k, \rho_k \sigma_{Y,j}^2 + \gamma_k^2 \sigma_{M,j}^2\right)$$

for $j = 1, \cdots, p$ and $k = 1, \cdots, K$. Given $\{R_j\}$, the corresponding log-likelihood function is defined as

$$
\begin{aligned}
l(\boldsymbol{\theta}) &= \sum_{j=1}^{p} \log \left\{ \sum_{k=1}^{K} \pi_k \phi\left(R_j; s_k, \rho_k \sigma_{Y,j}^2 + \gamma_k^2 \sigma_{M,j}^2\right) \right\} \\
&:= \sum_{j=1}^{p} \log \left\{ \sum_{k=1}^{K} \pi_k f_{jk}\left(\widehat{\beta}_{Y,j}, \widehat{\beta}_{M,j}; \boldsymbol{\theta}\right) \right\},
\end{aligned}
\tag{S3}
$$

where $\phi(\cdot; \mu, \sigma^2)$ is the density function of $\mathcal{N}(\mu, \sigma^2)$ and we define

$$
f_{jk}\left(\widehat{\beta}_{Y,j}, \widehat{\beta}_{M,j}; \boldsymbol{\theta}\right) = \phi\left(\widehat{\beta}_{Y,j} - \gamma_k \widehat{\beta}_{M,j}; s_k, \rho_k \sigma_{Y,j}^2 + \gamma_k^2 \sigma_{M,j}^2\right)
$$

to simplify the notation.

Since $\{R_j\}$ is unknown, we use the lower bound maximization algorithm [11] to estimate the parameters in (S3). The algorithm proceeds iteratively. Denote $\boldsymbol{\theta}^{(t)}$ as the estimated parameters at step t for $t = 1, \cdots, T$. According to Jensen's inequality, (S3) can be lower bounded by

$$
\begin{aligned}
l(\boldsymbol{\theta}) &= \sum_{j=1}^{p} \log \left\{ \sum_{k=1}^{K} w_{jk}^{(t)} \frac{\pi_k f_{jk}\left(\widehat{\beta}_{Y,j}, \widehat{\beta}_{M,j}; \boldsymbol{\theta}\right)}{w_{jk}^{(t)}} \right\} \\
&\geq \sum_{j=1}^{p} \sum_{k=1}^{K} w_{jk}^{(t)} \log \left\{ \frac{\pi_k f_{jk}\left(\widehat{\beta}_{Y,j}, \widehat{\beta}_{M,j}; \boldsymbol{\theta}\right)}{w_{jk}^{(t)}} \right\} := B\left(\boldsymbol{\theta}; \boldsymbol{\theta}^{(t)}\right),
\end{aligned}
$$

where $\{\mathbf{w}_j\} = \left(w_{jk}^{(t)}\right)_{k=1}^{K}$ are p arbitrary probability distributions depending on $\boldsymbol{\theta}^{(t)}$ with $\sum_{k=1}^{K} w_{jk}^{(t)} = 1$.

The optimal lower bound is achieved by maximizing $B\left(\boldsymbol{\theta}^{(t)}; \boldsymbol{\theta}^{(t)}\right)$ with respect to $w_{jk}^{(t)}$ under the constraint $\sum_{k=1}^{K} w_{jk}^{(t)} = 1$. Following the derivation in Section 7.8 of [11], we reformulate the constrained maximization problem into an unconstrained objective function by introducing the Lagrange multipliers $\{\lambda_j\}$. The reformulated objective function is given by

$$
\begin{aligned}
G\left(\mathbf{w}^{(t)}\right) = &\sum_{j=1}^{p} \lambda_j \left\{ \sum_{k=1}^{K} w_{jk}^{(t)} - 1 \right\} \\
&+ \sum_{j=1}^{p} \sum_{k=1}^{K} w_{jk}^{(t)} \left[\log\left(\pi_k^{(t)}\right) + \log\left\{ f_{jk}\left(\widehat{\beta}_{Y,j}, \widehat{\beta}_{M,j}; \boldsymbol{\theta}^{(t)}\right) \right\} - \log\left(w_{jk}^{(t)}\right) \right].
\end{aligned}
$$

By setting the derivatives of G with respect to $w_{jk}^{(t)}$ to zero and applying the constraint, we

obtain the solution for the weights

$$w_{jk}^{(t)} = \frac{\pi_k^{(t)} f_{jk}(\widehat{\beta}_{Y,j}, \widehat{\beta}_{M,j}; \boldsymbol{\theta}^{(t)})}{\left\{\sum_{\ell=1}^{K} \pi_\ell^{(t)} f_{j\ell}(\widehat{\beta}_{Y,j}, \widehat{\beta}_{M,j}; \boldsymbol{\theta}^{(t)})\right\}}.$$

The objective function is then updated by substituting these optimized weights into $B(\boldsymbol{\theta}; \boldsymbol{\theta}^{(t)})$. Subsequently, the parameters for the next iteration, $\boldsymbol{\theta}^{(t+1)}$, are obtained by maximizing $B(\boldsymbol{\theta}; \boldsymbol{\theta}^{(t)})$.

S3 Pleiotropy decomposition, deviation, and dispersion parameters

In this section, we derive and discuss the formulas in Section 2.1. As stated in (3), we assume $\delta_{U,j} \sim \mathcal{N}(\mu_{\delta_{U_k}}, \sigma^2_{\delta_{U_k}})$ and $\delta_{M,j} \sim \mathcal{N}(\mu_{\delta_M}, \sigma^2_{\delta_M})$ for $j \in \mathcal{J}_k$, with $\beta_{M,j} = \delta_{M,j} + \theta_{M,k}\delta_{U,j}$. Thus, $\beta_{M,j}$ follows the normal distribution $\mathcal{N}\big(\mu_{\delta_M} + \theta_{M,k}\mu_{\delta_{U_k}}, \sigma^2_{\delta_M} + \theta^2_{M,k}\sigma^2_{\delta_{U_k}}\big)$.

S3.1 Pleiotropy decomposition

We first derive the decomposition of $\delta_{U,j}$, which can be directly extended to $\theta_{Y,k}\delta_{U,j}$. The decomposition takes the form of $\delta_{U,j} = c_k\beta_{M,j} + (\delta_{U,j} - c_k\beta_{M,j})$, where c_k is a coefficient that ensures $\delta_{U,j} - c_k\beta_{M,j}$ is independent of $\beta_{M,j}$. To determine c_k, we calculate the covariance between $\delta_{U,j} - c_k\beta_{M,j}$ and $\beta_{M,j}$ as follows

$$\begin{aligned}\operatorname{cov}\big(\delta_{U,j} - c_k\beta_{M,j}, \beta_{M,j}\big) &= \operatorname{cov}\big(\delta_{U,j} - c_k\delta_{M,j} - c_k\theta_{M,k}\delta_{U,j}, \delta_{M,j} + \theta_{M,k}\delta_{U,j}\big)\\ &= \theta_{M,k}(1 - c_k\theta_{M,k})\sigma^2_{\delta_{U_k}} - c_k\sigma^2_{\delta_M}.\end{aligned}$$

Independence is achieved by solving c_k such that the covariance is zero because both $\delta_{U,j} - c_k\beta_{M,j}$ and $c_k\beta_{M,j}$ are normally distributed, which yields $c_k = \theta_{M,k}\sigma^2_{\delta_{U_k}} / \Big(\theta^2_{M,k}\sigma^2_{\delta_{U_k}} + \sigma^2_{\delta_M}\Big)$.

S3.2 Derivation of deviation parameters

Next, we determine the dispersion and deviation parameters. Recall that $\psi_j = \theta_{Y,k}(\delta_{U,j} - c_k\beta_{M,j})$. Under independence, the conditional expectation of $\delta_{U,j} - c_k\beta_{M,j}$ equals its marginal expectation

$$\begin{aligned}\mathbb{E}\big(\delta_{U,j} - c_k\beta_{M,j} \mid \beta_{M,j}\big) =&\mathbb{E}\big(\delta_{U,j} - c_k\beta_{M,j}\big)\\ =&\mu_{\delta_{U_k}} - c_k\big(\mu_{\delta_M} + \theta_{M,k}\mu_{\delta_{U_k}}\big)\\ =&\frac{\sigma^2_{\delta_M}}{\theta^2_{M,k}\sigma^2_{\delta_{U_k}} + \sigma^2_{\delta_M}}\mu_{\delta_{U_k}} - \frac{\theta_{M,k}\sigma^2_{\delta_{U_k}}}{\theta^2_{M,k}\sigma^2_{\delta_{U_k}} + \sigma^2_{\delta_M}}\mu_{\delta_M},\end{aligned}$$

which determines the deviation parameter

$$s_k = \theta_{Y,k}\left(\frac{\sigma^2_{\delta_M}}{\theta^2_{M,k}\sigma^2_{\delta_{U_k}} + \sigma^2_{\delta_M}}\mu_{\delta_{U_k}} - \frac{\theta_{M,k}\sigma^2_{\delta_{U_k}}}{\theta^2_{M,k}\sigma^2_{\delta_{U_k}} + \sigma^2_{\delta_M}}\mu_{\delta_M}\right).$$

The deviation parameter s_k equals zero under two specific conditions. First, s_k is zero when the unmeasured confounder has no effect on the outcome, which occurs when $\theta_{Y,k} = 0$. Second, s_k is also zero when the effects of the IV on both the exposure and the unmeasured confounder have a mean of zero, that is, when $\mu_{\delta_{U_k}} = \mu_{\delta_M} = 0$.

S3.3 Derivation of dispersion parameters

Similarly, the conditional and marginal variances of $\delta_{U,j} - c_k\beta_{M,j}$ are given by

$$\operatorname{var}\big(\delta_{U,j} - c_k\beta_{M,j} \mid \beta_{M,j}\big) = \operatorname{var}\big(\delta_{U,j} - c_k\beta_{M,j}\big) = \frac{\sigma^2_{\delta_M}\sigma^2_{\delta_{U_k}}}{\theta^2_{M,k}\sigma^2_{\delta_{U_k}} + \sigma^2_{\delta_M}}.$$

The dispersion parameter ρ_k in (1) can be determined by minimizing the Kullback-Leibler divergence between $\big\{\widetilde{\psi}_j : j \in \mathcal{J}_k\big\}$ with $\widetilde{\psi}_j \sim \mathcal{N}\big(s_k, (\rho_k - 1)\sigma^2_{Y,j}\big)$ and $\big\{\theta_{Y,k}(\delta_{U,j} - c_k\beta_{M,j}) : j \in \mathcal{J}_k\big\}$, which can be calculated as

$$\frac{1}{2}\sum_{j\in\mathcal{J}_k}\frac{\theta^2_{Y,k}(\theta^2_{M,k}\sigma^2_{\delta_{U_k}} + \sigma^2_{\delta_M})(\rho_k - 1)\sigma^2_{Y,j}}{\sigma^2_{\delta_{U_k}}\sigma^2_{\delta_M}} + \sum_{j\in\mathcal{J}_k}\log\left\{\frac{\theta^2_{Y,k}(\theta^2_{M,k}\sigma^2_{\delta_{U_k}} + \sigma^2_{\delta_M})}{\sigma^2_{\delta_{U_k}}\sigma^2_{\delta_M}}\right\} - \sum_{j\in\mathcal{J}_k}\log\left\{(\rho_k - 1)\sigma^2_{Y,j}\right\}$$

using the closed form in [12]. Finally, ρ_k can be approximated by

$$\rho_k \approx 1 + \left(|\mathcal{J}_k|^{-1}\sum_{j\in\mathcal{J}_k}\sigma^2_{Y,j}\right)^{-1}\frac{\sigma^2_{\delta_M}\sigma^2_{\delta_{U_k}}}{\theta^2_{Y,k}\left(\theta^2_{M,k}\sigma^2_{\delta_{U_k}} + \sigma^2_{\delta_M}\right)}$$

if $\theta_{Y,k} \neq 0$, and equals one if $\theta_{Y,k} = 0$.

S4 Baseline estimators

This section introduces baseline estimators that consider the measurement error of the estimated IV-exposure effects $\{\widehat{\beta}_{M,j}\}$. We first derive the estimators for the case when there is no pleiotropy, and then extend them to the case where balanced pleiotropy exists. Finally, we establish the connection between existing MR methods and these baseline estimators.

S4.1 Baseline estimators without pleiotropy

In this section, we introduce baseline estimators under the assumption of no pleiotropy. Recall that the estimated IV-exposure and IV-outcome effects follow the distribution

$$\begin{pmatrix} \widehat{\beta}_{M,j} \\ \widehat{\beta}_{Y,j} \end{pmatrix} \sim \mathcal{N}\left(\begin{pmatrix} \beta_{M,j} \\ \gamma_0 \beta_{M,j} \end{pmatrix}, \begin{pmatrix} \sigma^2_{M,j} & 0 \\ 0 & \sigma^2_{Y,j} \end{pmatrix} \right), \tag{S4}$$

where $\beta_{M,j}$ and $\gamma_0 \beta_{M,j}$ are the IV-exposure and IV-outcome effects, respectively, and γ_0 is the causal effect of exposure on outcome. Here, we omit the subscript and use γ because there is only one group. We introduce $\mathsf{MLE}_{\mathsf{ratio}}$, $\mathsf{MLE}_{\mathsf{pair}}$, MPLE, MQLE, and MoE estimators one by one.

First, the $\mathsf{MLE}_{\mathsf{ratio}}$ estimator is obtained by analyzing the distribution of the individual causal effects $\widehat{\gamma}_j = \widehat{\beta}_{Y,j} / \widehat{\beta}_{M,j}$ whose variance is estimated by $\widehat{\mathrm{sd}}^2_{\widehat{\gamma}_j} = \sigma^2_{Y,j}/\widehat{\beta}^2_{M,j} + \widehat{\beta}^2_{Y,j}\sigma^2_{M,j}/\widehat{\beta}^4_{M,j}$ using the delta method. Under some regularity conditions, $\widehat{\gamma}_j$ approximately follows $\mathcal{N}\big(\gamma, \widehat{\mathrm{sd}}^2_{\widehat{\gamma}_j}\big)$. Therefore, this problem can be formulated as estimating the unknown mean γ from a set of independent normally distributed observations $\{\widehat{\gamma}_j\}$ with known variances $\big\{\widehat{\mathrm{sd}}^2_{\widehat{\gamma}_j}\big\}$. In this case, the $\mathsf{MLE}_{\mathsf{ratio}}$ estimator maximizes the following log-likelihood of γ:

$$l_{\mathsf{ratio}}(\gamma) = -\frac{1}{2}\sum_{j=1}^{p} \left\{ \log\left(2\pi \widehat{\mathrm{sd}}^2_{\widehat{\gamma}_j}\right) + \frac{(\widehat{\gamma}_j - \gamma)^2}{\widehat{\mathrm{sd}}^2_{\widehat{\gamma}_j}} \right\}.$$

Second, the $\mathsf{MLE}_{\mathsf{pair}}$ estimator is obtained by analyzing the distribution of the pair of IV-exposure and IV-outcome effects $\big\{\big(\widehat{\beta}_{Y,j}, \widehat{\beta}_{M,j}\big)\big\}$. Under the model, $(\widehat{\beta}_{Y,j}, \widehat{\beta}_{M,j})$ are independent across j and each pair follows a bivariate normal distribution (S4). Therefore, this problem can be formulated as estimating the unknown parameter γ and $\{\beta_{M,j}\}$ from these independent observations. In this case, the $\mathsf{MLE}_{\mathsf{pair}}$ estimator maximizes the following log-likelihood function with respect to γ and $\{\beta_{M,j}\}$

$$l_{\mathsf{pair}}(\gamma, \{\beta_{M,j}\}) = -\frac{1}{2}\sum_{j=1}^{p} \left\{ \log\left(2\pi\sigma^2_{Y,j}\sigma^2_{M,j}\right) + \frac{(\widehat{\beta}_{Y,j} - \gamma\beta_{M,j})^2}{\sigma^2_{Y,j}} + \frac{(\widehat{\beta}_{M,j} - \beta_{M,j})^2}{\sigma^2_{M,j}} \right\}. \tag{S5}$$

The $\mathsf{MLE}_{\mathsf{pair}}$ estimator is obtained by iteratively updating γ and $\beta_{M,j}$ to maximize $l_{\mathtt{pair}}(\gamma, \{\beta_{M,j}\})$.

Third, $\{\beta_{M,j}\}$ in (S5) are nuisance parameters that can be eliminated by the profiled likelihood approach [22]. The MPLE estimator is obtained by maximizing the profiled log-likelihood

$$pl(\gamma) = \max_{\{\beta_{M,j}\}} l_{\mathsf{pair}}(\gamma, \{\beta_{M,j}\}) = -\frac{1}{2}\sum_{j=1}^{p} \left\{ \log\left(2\pi\sigma^2_{Y,j}\sigma^2_{M,j}\right) + \frac{(\widehat{\beta}_{Y,j} - \gamma\widehat{\beta}_{M,j})^2}{\sigma^2_{Y,j} + \gamma^2\sigma^2_{M,j}} \right\}.$$

Fourth, we fix γ and denote the residuals as $r_j(\gamma) = \widehat{\beta}_{Y,j} - \gamma\widehat{\beta}_{M,j}$. When $\gamma = \gamma_0$, we have $r_j(\gamma_0) \sim \mathcal{N}(0, \sigma^2_{Y,j} + \gamma_0^2\sigma^2_{M,j})$ according to (S4). The marginal distribution motivates a

quasi-log-likelihood of γ, defined as

$$ql(\gamma) = -\frac{1}{2}\sum_{j=1}^{p}\left\{\log(\sigma_{Y,j}^2 + \gamma^2\sigma_{M,j}^2) + \frac{(\widehat{\beta}_{Y,j} - \gamma\widehat{\beta}_{M,j})^2}{\sigma_{Y,j}^2 + \gamma^2\sigma_{M,j}^2}\right\}.$$

The MQLE estimator is obtained by maximizing $ql(\gamma)$.

Lastly, by setting $K = 1$ and $s_1 = 0$ in the derivation in Supplementary Section S1, we obtain the MoE estimator. As discussed in Section S1, the MoE estimator is equivalent to the dIVW estimator proposed by [21], but it is derived in a different manner: it corrects the bias in the moments of the estimating equations.

S4.2 Baseline estimators with balanced pleiotropy

In this section, we extend the methods in Section S4.1 to scenarios involving balanced pleiotropy modeled by a mean-zero normal distribution. Fixing $K = 1$ and setting deviation parameter s_1 as zero in (1), we have $\psi_j \sim \mathcal{N}(0, (\rho_1 - 1)\sigma_{Y,j}^2)$ for some $\rho_1 \geq 1$. The joint distribution of IV-exposure and IV-outcome effects is equal to

$$\begin{pmatrix} \widehat{\beta}_{M,j} \\ \widehat{\beta}_{Y,j} \end{pmatrix} \sim \mathcal{N}\left(\begin{pmatrix} \beta_{M,j} \\ \gamma_0\beta_{M,j} \end{pmatrix}, \begin{pmatrix} \sigma_{M,j}^2 & 0 \\ 0 & \rho_1\sigma_{Y,j}^2 \end{pmatrix}\right), \tag{S6}$$

where γ_0 is the causal effect and ρ_1 is the dispersion parameter. Similar to (S4), we omit the subscript and use γ and ρ because there is only one group. Compared to (S4), the variance of $\widehat{\beta}_{Y,j}$ in (S6) is multiplied by the factor ρ due to the presence of balanced pleiotropy. Both γ_0 and ρ_1 are unknown and require estimation. We introduce the five modified methods, while keeping the names of the estimators unchanged.

First, we derive the $\mathsf{MLE}_{\mathsf{ratio}}$ estimator by analyzing the distribution of the individual causal effect $\widehat{\gamma}_j$. Under suitable regularity conditions, $\widehat{\gamma}_j$ approximately follows normal distribution $\mathcal{N}(\gamma, \widehat{\mathrm{sd}}_{\widehat{\gamma}_j}^2)$, where $\widehat{\mathrm{sd}}_{\widehat{\gamma}_j}^2 = \rho\sigma_{Y,j}^2/\widehat{\beta}_{M,j}^2 + \widehat{\beta}_{Y,j}^2\sigma_{M,j}^2/\widehat{\beta}_{M,j}^4$ is the variance estimated using the delta method. Compared with the $\mathsf{MLE}_{\mathsf{ratio}}$ estimator in Section S4.1, the variance term here is multiplied by the dispersion parameter ρ. Taking balanced pleiotropy into account, the $\mathsf{MLE}_{\mathsf{ratio}}$ estimators maximize

$$l_{\mathsf{ratio}}(\gamma, \rho) = -\frac{1}{2}\sum_{j=1}^{p}\left[\log\left\{2\pi\left(\frac{\rho\sigma_{Y,j}^2}{\widehat{\beta}_{M,j}^2} + \frac{\widehat{\beta}_{Y,j}^2\sigma_{M,j}^2}{\widehat{\beta}_{M,j}^4}\right)\right\} + \frac{(\widehat{\gamma}_j - \gamma)^2}{\rho\sigma_{Y,j}^2/\widehat{\beta}_{M,j}^2 + \widehat{\beta}_{Y,j}^2\sigma_{M,j}^2/\widehat{\beta}_{M,j}^4}\right].$$

Second, we estimate unknown parameters γ, ρ, and $\{\beta_{M,j}\}$ based on the pair of IV-exposure and IV-outcome effects $\{(\widehat{\beta}_{Y,j}, \widehat{\beta}_{M,j})\}$. According to the model in (S6), the log-likelihood for these parameters is given by

$$l_{\mathsf{pair}}(\gamma, \rho, \{\beta_{M,j}\}) = -\frac{1}{2}\sum_{j=1}^{p}\left\{\log\left(2\pi\rho\sigma_{Y,j}^2\sigma_{M,j}^2\right) + \frac{(\widehat{\beta}_{Y,j} - \gamma\beta_{M,j})^2}{\rho\sigma_{Y,j}^2} + \frac{(\widehat{\beta}_{M,j} - \beta_{M,j})^2}{\sigma_{M,j}^2}\right\}. \tag{S7}$$

The $\mathsf{MLE}_{\mathsf{pair}}$ estimators are obtained by iteratively updating γ, ρ, and $\{\beta_{M,j}\}$ to maximize

$l_{\texttt{pair}}(\gamma, \rho, \{\beta_{M,j}\})$.

Third, after profiling nuisance parameters $\{\beta_{M,j}\}$ in (S7), the profiled log-likelihood can be calculated as follows

$$pl(\gamma, \rho) = \max_{\{\beta_{M,j}\}} l_{\texttt{pair}}(\gamma, \rho, \{\beta_{M,j}\}) = -\frac{1}{2}\sum_{j=1}^{p}\left\{\log\left(2\pi\rho\sigma_{Y,j}^2\sigma_{M,j}^2\right) + \frac{(\widehat{\beta}_{Y,j} - \gamma\widehat{\beta}_{M,j})^2}{\rho\sigma_{Y,j}^2 + \gamma^2\sigma_{M,j}^2}\right\}.$$

The MPLE estimators are obtained by maximizing $pl(\gamma, \rho)$.

Fourth, fixing $\gamma = \gamma_0$, we have $r_j(\gamma_0) \sim \mathcal{N}(0, \rho_1\sigma_{Y,j}^2 + \gamma_0^2\sigma_{M,j}^2)$ according to (S6). A quasi-log-likelihood of γ and ρ is defined as

$$ql(\gamma, \rho) = -\frac{1}{2}\sum_{j=1}^{p}\left\{\log(\rho\sigma_{Y,j}^2 + \gamma^2\sigma_{M,j}^2) + \frac{(\widehat{\beta}_{Y,j} - \gamma\widehat{\beta}_{M,j})^2}{\rho\sigma_{Y,j}^2 + \gamma^2\sigma_{M,j}^2}\right\}.$$

The MQLE estimators are obtained by maximizing $ql(\gamma, \rho)$.

Lastly, the MoE estimator of γ_0, denoted as $\widehat{\gamma}_{\mathsf{MoE}}$ is invariant to the value of ρ_1 because of the form of (S2). To estimate ρ_1, we calculate the expectation of $(\widehat{\beta}_{Y,j} - \gamma_0\widehat{\beta}_{M,j})^2$ as follows,

$$\begin{aligned} p^{-1}\sum_{j=1}^{p}\mathbb{E}\left(\widehat{\beta}_{Y,j} - \gamma_0\widehat{\beta}_{M,j}\right)^2 =& p^{-1}\sum_{j=1}^{p}\mathrm{Var}\left(\widehat{\beta}_{Y,j} - \gamma_0\widehat{\beta}_{M,j}\right) + p^{-1}\sum_{j=1}^{p}\left\{\mathbb{E}\left(\widehat{\beta}_{Y,j} - \gamma_0\widehat{\beta}_{M,j}\right)\right\}^2 \\ =& p^{-1}\sum_{j=1}^{p}\left(\rho\sigma_{Y,j}^2 + \gamma_0^2\sigma_{M,j}^2\right). \end{aligned}$$

Plugging $\widehat{\gamma}_{\mathsf{MoE}}$ into the above equation and replacing the left-hand side with its empirical version, we obtain the MoE estimator of ρ by solving the following equation

$$p^{-1}\sum_{j=1}^{p}\left(\widehat{\beta}_{Y,j} - \widehat{\gamma}_{\mathsf{MoE}}\widehat{\beta}_{M,j}\right)^2 \approx \rho\left(p^{-1}\sum_{j=1}^{p}\sigma_{Y,j}^2\right) + \widehat{\gamma}_{\mathsf{MoE}}^2\left(p^{-1}\sum_{j=1}^{p}\sigma_{M,j}^2\right).$$

S4.3 A summary of MR methods

Supplementary Table S1 presents a summary of two-sample MR methods reviewed in Sanderson et al. [15]. The methods are organized into six categories based on their strategies for modeling IV-exposure and IV–outcome associations. These categories include five commonly used baseline estimators and their extensions, as well as a class of methods that do not explicitly consider measurement error. For each method, we assess robustness to measurement error and various forms of pleiotropy (balanced, directional, uncorrelated, and correlated), and key methodological features are also described.

Type	Methods	A1	A2	A3	A4	A5	Additional Performance or Comments
ignore ME	IVW [3]	×	×	×	×	×	Requires all IVs to be valid.
	Egger [1]	×	✓	✓	×	×	Limited statistical power.
	Median [2]	×	×	×	✓	✓	Consistent if the majority of IVs are valid.
	MBE [7]	×	×	×	✓	✓	Consistent if the largest cluster of IVs is valid.
	MIXIE [10]	×	✓	✓	✓	×	Assumes NOME; leverages information from IVs exhibiting uncorrelated pleiotropy.
	CIV [9], MRLASSO / MRRobust [14], regularization [6]	×	×	×	✓	✓	Requires some valid IVs; utilizes penalized parameters to model and sparsify pleiotropy effects.
$\mathsf{MLE}_{\mathsf{ratio}}$	baseline	×	×	×	×	×	Requires sufficiently strong IV-exposure effects for approximate normality of $\widehat{\gamma}_j = \widehat{\beta}_{Y,j}/\widehat{\beta}_{M,j}$.
	ConMIX [4]	×	×	×	✓	✓	Employs a mixture model for pleiotropy adjustment.
	MRClust [5]	×	×	×	✓	✓	Assumes distinct causal mechanisms for IV clusters; includes a null and junk cluster to avoid spurious clusters.
	GSME [23]	×	×	×	✓	✓	Adjusts for pleiotropy via outlier detection.
$\mathsf{MLE}_{\mathsf{pair}}$	baseline	✓	×	×	×	×	Effectively adjusts for measurement error bias; dispersion parameters are not consistently estimable.
	cML [19]	✓	×	×	✓	✓	Requires a subset of valid IVs.
	Path [8]	✓	×	×	✓	✓	Uses mixture modeling with cluster-specific causal mechanisms.
	MRCIP [17]	✓	×	×	✓	✓	Assigns weights to diminish influence from likely-invalid IVs.
MPLE	baseline	✓	×	×	×	×	Performance is comparable to $\mathsf{MLE}_{\mathsf{pair}}$; can be modified for balanced pleiotropy correction.
	Raps [22]	✓	✓	×	✓	✓	Corrects for balanced pleiotropy; robust to extreme cases via Tukey's loss function.
	GRAPPLE [16]	✓	✓	×	✓	✓	Detects pleiotropy pathways with a multi-modality approach.
MQLE	baseline	×	✓	×	×	×	Analyzes the distribution of residuals $\widehat{\beta}_{Y,j} - \gamma\widehat{\beta}_{M,j}$; corrects the balanced pleiotropy.
	MIX [13]	×	✓	×	✓	✓	Uses a mixture model that distinguishes the valid and invalid IVs.
MoE	baseline / dIVW [21]	✓	✓	×	×	×	Simultaneously adjusts for measurement error and balanced pleiotropy; not applicable to directional pleiotropy.
	Local [18]	✓	✓	×	✓	✓	Screens out IVs affected by pleiotropy; requires the remaining IVs are valid or suffer from balanced pleiotropy.
This paper	dmIVW, $\mathsf{dmIVW}_{\mathsf{MA}}$	✓	✓	✓	✓	✓	Hybrid MoE/MQLE model; combines mixture modeling with conservative dispersion estimation to address measurement error, balanced, and directional pleiotropy.

Supplementary Table S1: **Categorization of MR methods and their properties.** A1 evaluates whether bias from measurement error is mitigated; A2 and A3 examine whether the method is applicable when all IVs demonstrate balanced or directional pleiotropy, respectively; A4 and A5 address applicability in the presence of uncorrelated or correlated pleiotropy among IVs, respectively. Methods that satisfy a particular condition are marked with a check mark (✓), while those that do not are marked with a cross (×) to indicate that the condition is not satisfied.

S5 Model identifiability

S5.1 Model identifiability with known group membership

Suppose that the group membership of each IV is known, that is, the sets $\mathcal{J}_k$ are known. Under the working model (1), the joint density of $(\widehat{\beta}_{M,j}, \widehat{\beta}_{Y,j})$ is

$$f_j(m_j, y_j; \rho_k, \gamma_k, s_k) = \frac{1}{\sqrt{(2\pi)^2 \rho_k \sigma_{M,j}^2 \sigma_{Y,j}^2}} \exp\left\{-\frac{1}{2}\left[\frac{(m_j - \beta_{M,j})^2}{\sigma_{M,j}^2} + \frac{(y_j - \gamma_k \beta_{M,j} - s_k)^2}{\rho_k \sigma_{Y,j}^2}\right]\right\},$$

for $j \in \mathcal{J}_k$. The joint density of all observations is therefore $\prod_{k=1}^K \prod_{j \in \mathcal{J}_k} f_j(m_j, y_j; \rho_k, \gamma_k, s_k)$.

Proposition S1. *Suppose $|\mathcal{J}_k| \geq 2$ and there exist $j_1, j_2 \in \mathcal{J}_k$ such that $\beta_{M,j_1} \neq \beta_{M,j_2}$ for all k, then the parameters $\{(\rho_k, \gamma_k, s_k)\}_{k=1}^K$ are identifiable. Conversely, if $\beta_{M,j} = c$ for some $c \in \mathbb{R}$ and all $j \in \mathcal{J}_k$, then γ_k and s_k are not separately identifiable.*

Proof of Proposition S1. Since the moment generating function (MGF) uniquely determines the distribution, it suffices to prove identifiability via the MGF. The MGF of $\{(\beta_{M,j}, \beta_{Y,j})\}_{j \in \mathcal{J}_k}$ is

$$M_k\left(\{t_{1,j}, t_{2,j}\}; \{(\rho_k, \gamma_k, s_k)\}_{k=1}^K\right) = \exp\left\{\sum_{j \in \mathcal{J}_k} t_{2,j}(\gamma_k \beta_{M,j} + s_k) + \frac{1}{2}\sum_{j \in \mathcal{J}_k} \rho_k \sigma_{Y,j}^2 t_{2,j}^2\right\} \times C,$$

where C is a constant independent of $\{(\rho_k, \gamma_k, s_k)\}_{k=1}^K$. Suppose there are two parameter sets $\{(\rho_k^{(1)}, \gamma_k^{(1)}, s_k^{(1)})\}_{k=1}^K$ and $\{(\rho_k^{(2)}, \gamma_k^{(2)}, s_k^{(2)})\}_{k=1}^K$ produce the same MGF as follows

$$\sum_{j \in \mathcal{J}_k}\left[t_{2,j}(\gamma_k^{(1)} \beta_{M,j} + s_k^{(1)}) + \frac{1}{2}\rho_k^{(1)} \sigma_{Y,j}^2 t_{2,j}^2\right] = \sum_{j \in \mathcal{J}_k}\left[t_{2,j}(\gamma_k^{(2)} \beta_{M,j} + s_k^{(2)}) + \frac{1}{2}\rho_k^{(2)} \sigma_{Y,j}^2 t_{2,j}^2\right].$$

Since this identity holds for all $\{t_{2,j}\}$, the coefficients must coincide. Comparing the coefficients of $t_{2,j}^2$ gives $\rho_k^{(1)} = \rho_k^{(2)}$. Comparing the coefficients of $t_{2,j}$ yields $\gamma_k^{(1)} \beta_{M,j} + s_k^{(1)} = \gamma_k^{(2)} \beta_{M,j} + s_k^{(2)}$, for $j \in \mathcal{J}_k$, which can be rearranged as $(\gamma_k^{(1)} - \gamma_k^{(2)})\beta_{M,j} = s_k^{(2)} - s_k^{(1)}$. If there exist $j_1, j_2 \in \mathcal{J}_k$ such that $\beta_{M,j_1} \neq \beta_{M,j_2}$, then applying the above equality to j_1 and j_2 implies $\gamma_k^{(1)} = \gamma_k^{(2)}$ and $s_k^{(1)} = s_k^{(2)}$. Hence (ρ_k, γ_k, s_k) is identifiable.

Conversely, if $\beta_{M,j} = c$ for some $c \in \mathbb{R}$ and all $j \in \mathcal{J}_k$, then only the combination $\gamma_k \beta_{M,j} + s_k$ is identifiable. Indeed, replacing (γ_k, s_k) by $(\gamma_k + \delta,\ s_k - \delta c)$ produces the same distribution for any $\delta \in \mathbb{R}$, and therefore γ_k and s_k are not separately identifiable. □

S5.2 Model identifiability with latent group membership

When the group membership of each IV is unknown, the distribution of the summary statistics becomes a mixture over the latent groups. This makes it difficult to directly compare the IV–exposure effects $\beta_{M,j}$ within the same group, as done in Supplementary Section S5.1. Therefore, we instead exploit the distributional structure of $\beta_{M,j}$ implied by the structural equation (2). Under the assumptions in Supplementary Section S3, $\beta_{M,j} \sim$

$\mathcal{N}\Big(\mu_{\delta_M} + \theta_{M,k}\mu_{\delta_{U,k}},\ \sigma^2_{\delta_M} + \theta^2_{M,k}\sigma^2_{\delta_{U,k}}\Big)$ for each $j \in \mathcal{J}_k$. Define $\mu_{M,k} = \mu_{\delta_M} + \theta_{M,k}\mu_{\delta_{U,k}}$ and $\tau^2_{M,k} = \sigma^2_{\delta_M} + \theta^2_{M,k}\sigma^2_{\delta_{U,k}}$. Denote the group to which the jth IV belongs as U_j, where $U_j = k$ if $j \in \mathcal{J}_k$. Assume that U_j follows a multinomial distribution, i.e., $U_j \sim \text{Multinomial}(\pi_1, \cdots, \pi_K)$ with $\sum_{k=1}^K \pi_k = 1$. Under this hierarchical specification, the estimated IV-exposure and IV-outcome effects, conditional on $U_j = k$, follow the distribution

$$\begin{pmatrix} \widehat{\beta}_{M,j} \\ \widehat{\beta}_{Y,j} \end{pmatrix} \mid U_j = k \sim \mathcal{N}\left(\mu^*_k, \Sigma^*_{k,j}\right),$$

where

$$\mu^*_k = \begin{pmatrix} \mu_{M,k} \\ \gamma_k \mu_{M,k} + s_k \end{pmatrix}, \qquad \Sigma^*_{k,j} = \begin{pmatrix} \tau^2_{M,k} + \sigma^2_{M,j} & \gamma_k \tau^2_{M,k} \\ \gamma_k \tau^2_{M,k} & \gamma^2_k \tau^2_{M,k} + \rho_k \sigma^2_{Y,j} \end{pmatrix}.$$

Therefore, the marginal distribution of $(\widehat{\beta}_{M,j}, \widehat{\beta}_{Y,j})$ is a finite mixture of bivariate normal distributions. Hence, for any fixed j_0, if the Gaussian component parameters $\{(\mu^*_k, \Sigma^*_{k,j_0})\}_{k=1}^K$ are pairwise distinct, then the unordered collection $\{(\pi_k, \mu^*_k, \Sigma^*_{k,j_0})\}_{k=1}^K$ is identifiable up to label switching [20].

In our setting, this result allows us to further study the identifiability condition of the underlying parameters. In particular, we have the following result.

Proposition S2. *If $\tau^2_{M,k} > 0$ for all $k = 1, \cdots, K$ and $\{(\mu_{M,k}, \tau^2_{M,k}, \gamma_k, s_k, \rho_k)\}_{k=1}^K$ are pairwise distinct, then $\{(\pi_k, \mu_{M,k}, \tau^2_{M,k}, \gamma_k, s_k, \rho_k)\}_{k=1}^K$ is identifiable up to label switching.*

Proof of Proposition S2. Fix the index j_0. From the previous result, the unordered collection $\{(\pi_k, \mu^*_k, \Sigma^*_{k,j_0})\}_{k=1}^K$ is identifiable up to label switching [20]. Since $\tau^2_{M,k} > 0$, the mapping $(\mu_{M,k}, \tau^2_{M,k}, \gamma_k, s_k, \rho_k) \mapsto (\mu^*_k, \Sigma^*_{k,j_0})$ is one-to-one because $\mu_{M,k} = (\mu^*_k)_1$, $\tau^2_{M,k} = (\Sigma^*_{k,j_0})_{11} - \sigma^2_{M,j_0}$, $\gamma_k = (\Sigma^*_{k,j_0})_{12}/\tau^2_{M,k}$, $s_k = (\mu^*_k)_2 - \gamma_k \mu_{M,k}$, $\rho_k = \{(\Sigma^*_{k,j_0})_{22} - \gamma^2_k \tau^2_{M,k}\}/\sigma^2_{Y,j_0}$. Hence $\{(\pi_k, \mu_{M,k}, \tau^2_{M,k}, \gamma_k, s_k, \rho_k)\}_{k=1}^K$ is identifiable up to label switching as well. □

Proposition S1 requires that, within each group, the IV–exposure effects $\beta_{M,j}$ are not identical, i.e., there exist at least two IVs with different $\beta_{M,j}$ values. Proposition S2 imposes a distributional condition that the group-specific variance $\tau^2_{M,k}$ is strictly positive. Together, Propositions S1 and S2 indicate that a key requirement for model identifiability is that the IV–exposure effects $\beta_{M,j}$ exhibit some heterogeneity within each group.

S6 Additional simulations

S6.1 Further discussion of five-group IV simulation

In this section, we provide a more detailed discussion of the results from the five-group IV simulation.

Overall performance. We included the same competing methods—dIVW$_{\text{valid}}$, dmIVW, Raps, and Local—as in the two-group IV setup. However, the bias and MSE of dIVW and Raps dominated the scale of the plots and were therefore omitted from the presentation. We considered our proposed methods with $K_{\max} \in \{2, 3, 5\}$, denoted as dmIVW$^{K_{\max}=2}$,

$\mathsf{dmIVW}^{K_{\max}=3}$, $\mathsf{dmIVW}^{K_{\max}=5}$ and $\mathsf{dmIVW}_{\mathsf{MA}}^{K_{\max}=2}$, $\mathsf{dmIVW}_{\mathsf{MA}}^{K_{\max}=3}$, $\mathsf{dmIVW}_{\mathsf{MA}}^{K_{\max}=5}$, respectively, and investigated the impact of different choices of $K_{\max}$. We evaluated the performance of the methods in terms of average absolute bias, average MSE, and coverage probability (Supplementary Fig. S1). Across all three cases, larger values of $K_{\max}$ generally resulted in smaller bias and MSE, as well as coverage probabilities closer to the nominal level. However, the differences among different $K_{\max}$ were modest in the first two cases. In Case 3, choosing $K_{\max} = 2$ led to noticeably larger bias and MSE compared with $K_{\max} \in \{3, 5\}$, along with under-coverage of the confidence intervals. In addition, $\mathsf{dmIVW}_{\mathsf{MA}}$ performed slightly better than dmIVW in terms of bias, MSE, and coverage probability.

Model selection behavior. To better understand these results, we further examined the model selection behavior of dmIVW and the model averaging weights of $\mathsf{dmIVW}_{\mathsf{MA}}$ under the three cases (Supplementary Figs. S2–S4). In Case 1, regardless of the choice of $K_{\max}$, the most frequently selected submodel was $\mathcal{M}_{4K_{\max}+1}$, the two-group submodel with correlated pleiotropy ($s_1 = 0$, $\rho_1 = 1$). When $K_{\max} \in \{3, 5\}$, the second most frequently selected submodel was $\mathcal{M}_{4K_{\max}+5}$, which had the same specification except that it involved three groups. In Case 2, when $K_{\max} \in \{3, 5\}$, the submodel $\mathcal{M}_{4K_{\max}+5}$ became the most frequently selected model, identifying a structure with correlated pleiotropy. In contrast, when $K_{\max} = 2$, the selected model was $\mathcal{M}_5$, corresponding to the submodel assuming uncorrelated pleiotropy. Therefore, when $K_{\max}$ was chosen too small, correlated pleiotropy could be mistakenly approximated by an uncorrelated pleiotropy structure in this case. In Case 3, when equipped with $K_{\max} = 2, 3$, and 5, the selected models were $\mathcal{M}_{4K_{\max}+1}$, $\mathcal{M}_{4K_{\max}+5}$, and $\mathcal{M}_{4K_{\max}+9}$, respectively, which corresponded to submodels with two-, three-, and four-group correlated pleiotropy, respectively, with the deviation and dispersion parameters fixed at $s_1 = 0$ and $\rho_1 = 1$. In this case, a two-group specification was insufficient to adequately capture the heterogeneous pleiotropic structure and led to the inferior performance observed when $K_{\max} = 2$.

Illustrative Replication. To further illustrate the model selection mechanism, we examined the fitting results from a representative replication (Supplementary Fig. S5). This replication was chosen because the submodels selected by $\mathsf{dmIVW}^{K_{\max}=2}$, $\mathsf{dmIVW}^{K_{\max}=3}$, and $\mathsf{dmIVW}^{K_{\max}=5}$ coincided with the most frequently selected submodels reported in Supplementary Figs. S2–S4. The ground-truth configuration in Case 1 shed light on why the two-group submodel $\mathcal{M}_{4K_{\max}+1}$ could still be frequently selected even when $K_{\max} > 2$. Although the invalid IV groups were associated with different parameters in the data-generating process, their empirical distributions largely overlapped in this scenario. Consequently, grouping these IVs into a single pleiotropic group provided an economical approximation while maintaining a good model fit. This phenomenon also explained why $\mathsf{dmIVW}^{K_{\max}=5}$ selected submodels with three and four pleiotropic groups in Cases 2 and 3, respectively. In Case 2, $\mathsf{dmIVW}^{K_{\max}=2}$ incorrectly selected an uncorrelated pleiotropy structure. However, since the invalid IVs were relatively evenly distributed, this misspecification had only a limited impact on the estimation accuracy. This suggested that in some scenarios, specifying a $K_{\max}$ smaller than the true number of pleiotropic groups could still yield a reasonable approximation.

On the other hand, in Case 3, although $\mathsf{dmIVW}^{K_{\max}=2}$ correctly identified the valid IV group when $\mathcal{M}_{4K_{\max}+1}$ was selected, the resulting absolute bias and MSE (Supplementary Fig. S1) were unstable. This instability appeared to be driven by the frequent selection of $\mathcal{M}_5$, which occurred in about 31% of the replications. Among the 100 replications, the

averaged absolute bias and averaged MSE were 0.024 and 8.7×10^{-4} when $\mathcal{M}_5$ was selected, compared with 0.015 and 3.6×10^{-4} when $\mathcal{M}_{4K_{\max}+1}$ was selected. This suggested that when the pleiotropic structure was more heterogeneous, specifying a $K_{\max}$ that was too small could lead to unstable model selection and worse estimation accuracy.

Based on these results, we believe that $K_{\max} = 3$ is sufficient to capture the main pleiotropic structure in this scenario. The additional group allows the model to accommodate pleiotropic effects both larger and smaller than the causal effect, which effectively mitigates most of the bias induced by heterogeneous pleiotropy. Therefore, considering both estimation accuracy and computational efficiency, we recommend using $K_{\max} = 3$ in practice.

Group selection accuracy. Finally, Supplementary Table S2 summarized the estimation accuracy of our group selection procedure. When a submodel with correlated pleiotropy was selected, the group identified by our causal group selection procedure was almost always the one closest to the true causal effect. The only notable exception occurred for $\mathsf{dmIVW}^{K_{\max}=5}$ in Case 3. This arose because, in the data-generating process, one group exhibited uncorrelated pleiotropy whose mean effect coincided with the true causal effect. Overall, our procedure consistently identified the group of valid IVs. Moreover, the absolute bias and RMSE of the selected group were substantially smaller than those of the remaining groups, even when compared with the group that had the smallest bias among the remaining components. This finding also helped explain the results in our two-group simulations, where our method accurately estimated the causal effect because the proposed group selection procedure correctly identified the valid IV group. These results demonstrated that the proposed method could reliably distinguish the causal group from other pleiotropic groups.

Supplementary Table S2: **Performance when the selected submodel assumes correlated pleiotropy.** "Num." denotes the number of simulation replicates in which the selected submodel assumes correlated pleiotropy. "Sel. Grp." denotes the group identified as the causal group by our group selection procedure. Among the remaining groups, "Rest Grp." denotes the group whose estimated effect is closest to the true causal effect. "Prop." is the proportion of replicates in which the selected group is the component whose estimated effect is closest to the true causal effect among all components in the model.

Case	$K_{\max}$	Num.	Prop.(%)	Abs. Bias (Sel. Grp.)	Abs. Bias. (Rest Grp.)	RMSE (Sel. Grp.)	RMSE (Rest Grp.)
Case 1	2	100	100	0.016	1.812	0.019	1.813
	3	100	100	0.015	1.696	0.018	1.714
	5	100	100	0.014	1.681	0.018	1.700
Case 2	2	6	100	0.018	0.264	0.020	0.267
	3	100	100	0.014	1.892	0.017	1.894
	5	100	100	0.014	1.869	0.017	1.873
Case 3	2	69	100	0.015	0.342	0.020	0.351
	3	100	100	0.014	1.617	0.017	1.637
	5	100	94	0.013	0.107	0.016	0.137

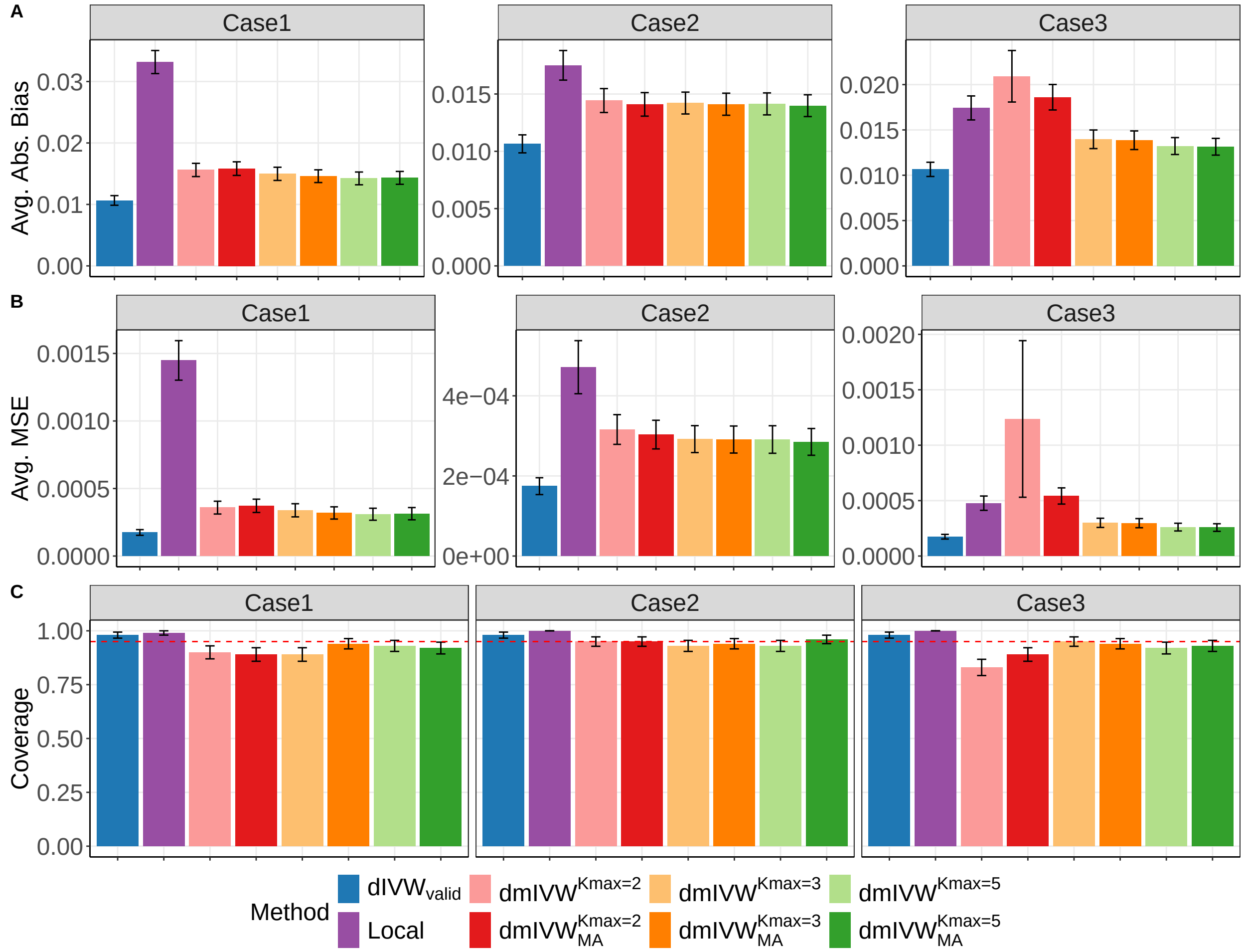

Supplementary Figure S1: **Comparisons of methods under five-group IV setups.** The simulation was repeated 100 times, and the confidence level was set as 95%. The absolute bias and mean square error (MSE), and coverage were averaged over all replications.

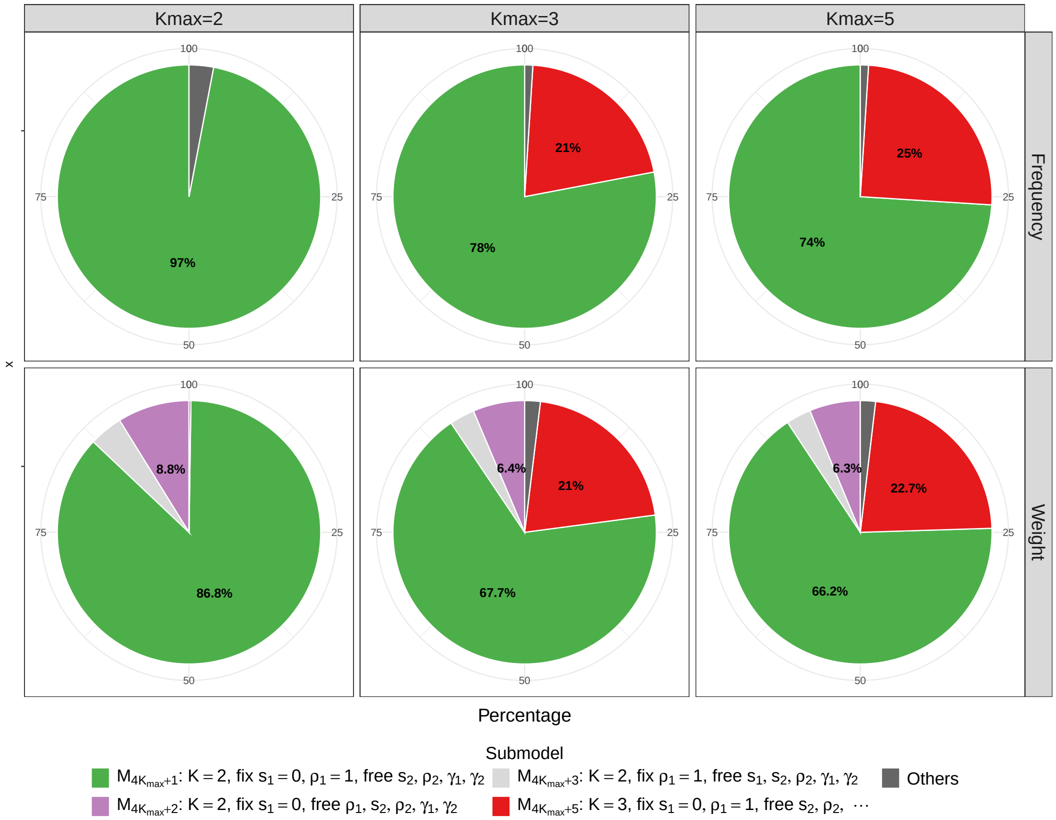

Supplementary Figure S2: **The model selection of dmIVW and model averaging of dmIVW$_{\text{MA}}$ under Case 1.** The upper panel displays the frequency of submodels selected by dmIVW with $K_{\max} \in \{2, 3, 5\}$, while the lower panel presents the model-averaging weights of dmIVW$_{\text{MA}}$ with $K_{\max} \in \{2, 3, 5\}$ averaged over replications.

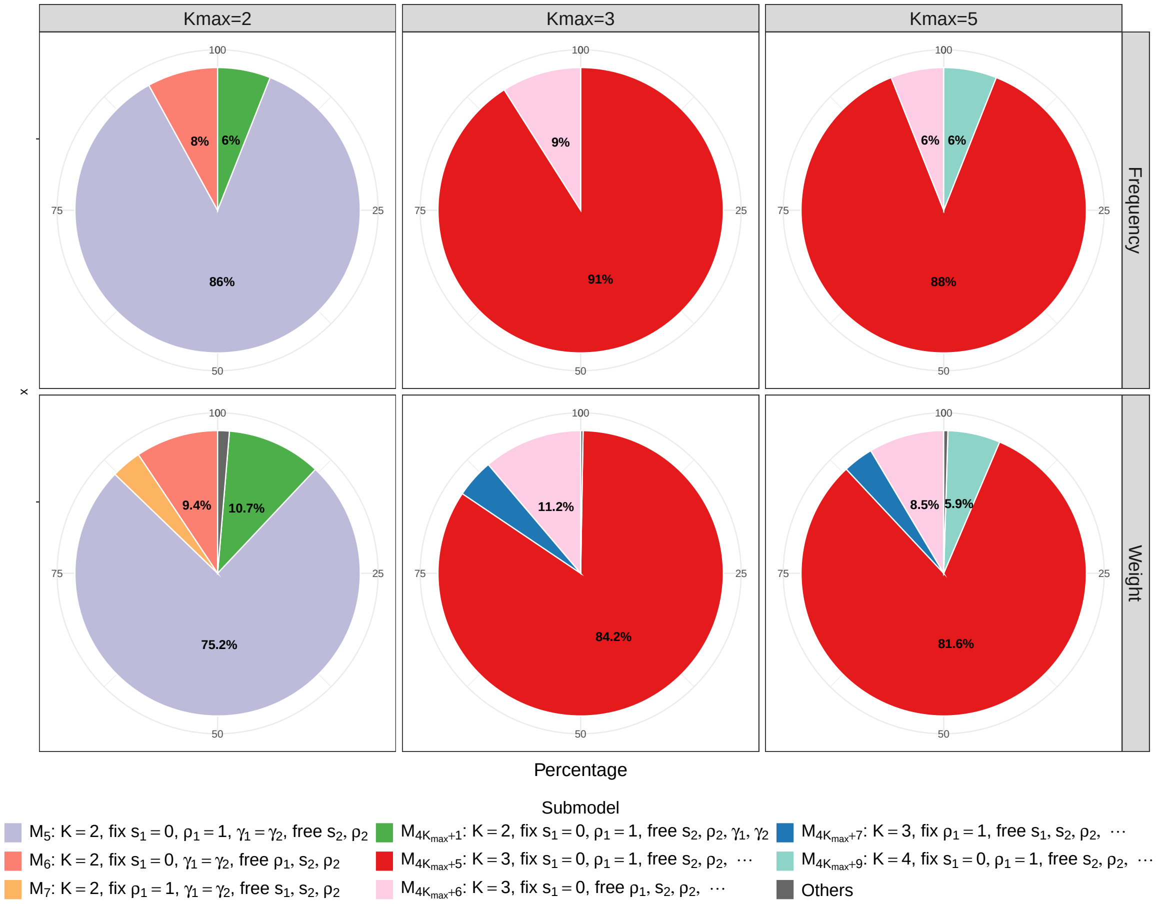

Supplementary Figure S3: **The model selection of dmlVW and model averaging of dmlVW$_{\text{MA}}$ under Case 2.** The upper panel displays the composition of submodels selected by dmlVW with $K_{\max} \in \{2, 3, 5\}$, while the lower panel presents the model-averaging weights of dmlVW$_{\text{MA}}$ with $K_{\max} \in \{2, 3, 5\}$, averaged over replicates.

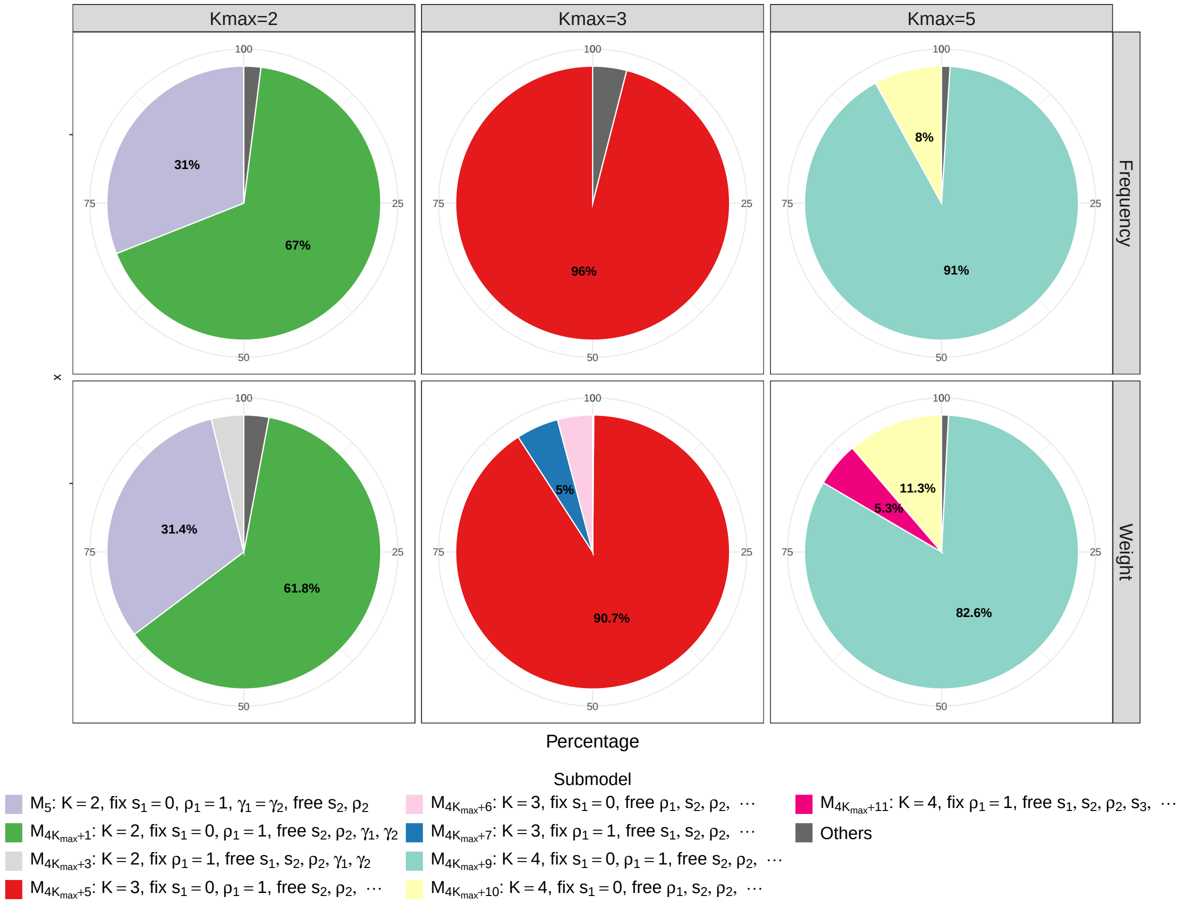

Supplementary Figure S4: **The model selection of dmIVW and model averaging of $\mathsf{dmIVW}_{\mathsf{MA}}$ under Case 3.** The upper panel displays the composition of submodels selected by dmIVW with $K_{\max} \in \{2, 3, 5\}$, while the lower panel presents the model-averaging weights of $\mathsf{dmIVW}_{\mathsf{MA}}$ with $K_{\max} \in \{2, 3, 5\}$, averaged over replicates.

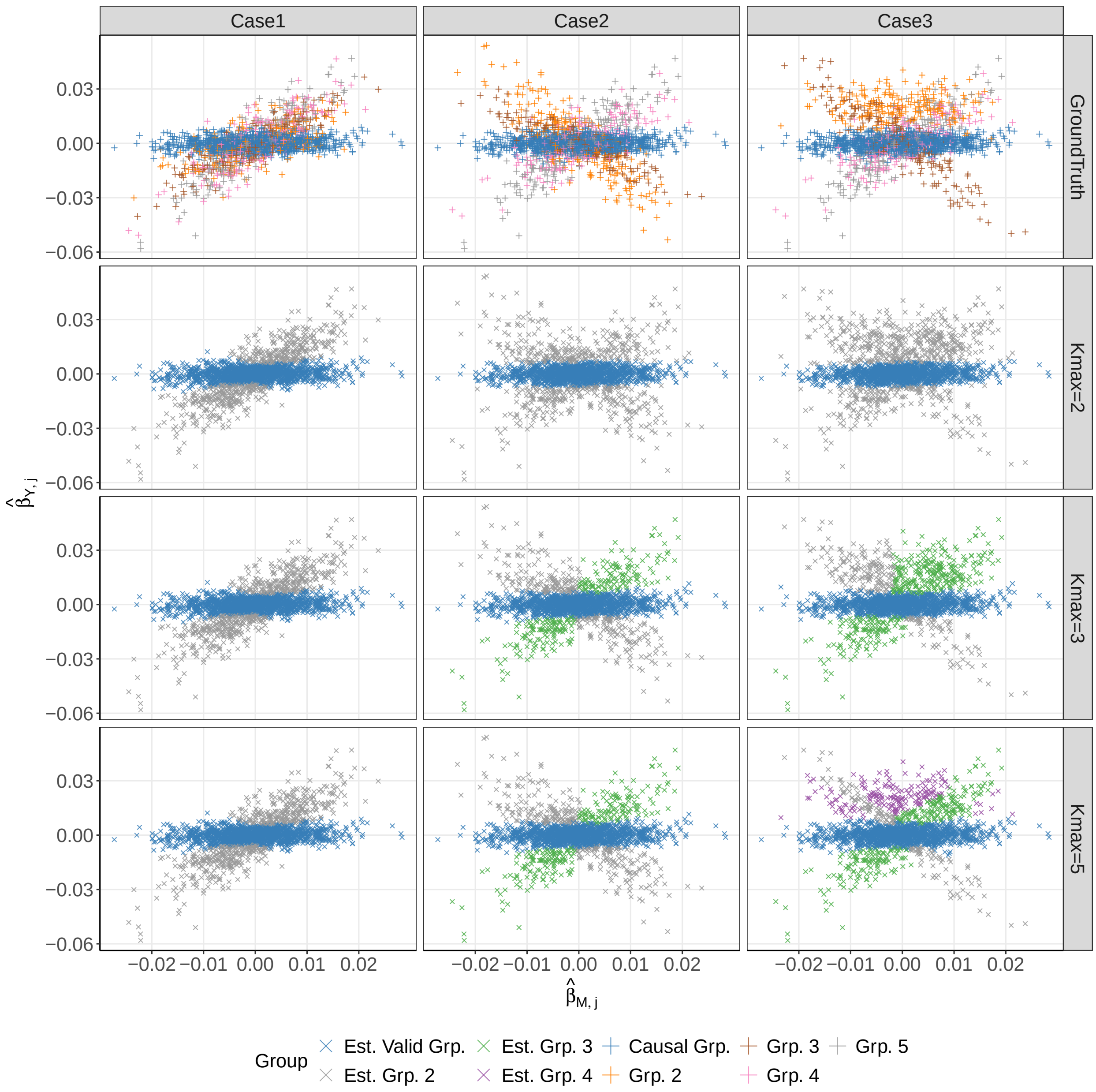

Supplementary Figure S5: **Illustration of the ground-truth and estimated IV groups for a representative replication.** The "GroundTruth" panel shows the true group membership of IVs in the data-generating process, where different colors correspond to different pleiotropic groups and blue "+" marks denote the valid IV group. The panels with $K_{\max} = 2, 3$, and 5 display the estimated group assignments obtained from our method, where different colors denote different groups. For the jth IV, the estimated group was determined by $\arg\max_k w_{jk}^{(t)}$ where $w_{jk}^{(t)}$ is defined in **Method** Section. The identified causal group is indicated by blue "×" marks. In this replication, the selected submodels differed across the three cases. In Case 1, for all $K_{\max} \in \{2, 3, 5\}$ the selected submodel was $\mathcal{M}_{4K_{\max}+1}$. In Case 2, the selected submodel was $\mathcal{M}_5$ when $K_{\max} = 2$, and $\mathcal{M}_{4K_{\max}+5}$ when $K_{\max} \in \{3, 5\}$. In Case 3, the selected submodels were $\mathcal{M}_{4K_{\max}+1}$, $\mathcal{M}_{4K_{\max}+5}$, and $\mathcal{M}_{4K_{\max}+9}$ for $K_{\max} = 2, 3$, and 5, respectively.

S6.2 Semi-synthetic simulation with residual linkage disequilibrium based on the BMI–CAD analysis

Since LD pruning is often imperfect in practice, we conducted an additional simulation to assess the robustness of our methods to residual linkage disequilibrium (LD) using the BMI–CAD dataset from our second real-data application. Specifically, we extracted SNP-level summary statistics $\{(\tilde{\beta}_{M,j}, \tilde{\beta}_{Y,j}, \tilde{\sigma}^2_{M,j}, \tilde{\sigma}^2_{Y,j})\}_{j=1}^p$ and the corresponding LD matrix for $p = 316$ SNPs. For each simulation replicate, the estimated SNP–exposure effects $\tilde{\beta}_{M,j}$ were treated as the true exposure effects, and the SNP–outcome effects were generated assuming a causal effect of 0.4. Two data-generating settings were considered. In the *No LD* setting, the simulated SNP–exposure and SNP–outcome summary statistics were generated independently. In the *LD* setting, modest residual LD was introduced by generating the summary statistics from multivariate normal distributions with covariance structures induced by the empirical LD matrix. The simulation results are summarized in Supplementary Table S3. Across methods, the performance under the LD setting was comparable to that under the No LD setting, suggesting that the residual correlation after LD clumping was modest and had little impact on the performance of the proposed methods.

Supplementary Table S3: **Simulation results under independent-SNP and LD-aware data generation ($\gamma_0 = 0.4$).** "No LD" assumes that SNP effects are generated independently, whereas "LD" generates summary statistics with covariance structures induced by empirical LD matrices. Results were based on 100 simulation replicates, summarizing bias (multiplied by 100 for ease of comparison), root mean square error (RMSE), and empirical coverage at the 95% confidence level.

Criterion	IVW		Egger		dIVW		Raps		dmIVW		dmIVW_{MA}	
	No LD	LD	No LD	LD	No LD	LD	No LD	LD	No LD	LD	No LD	LD
Bias(×100)	-4.176	-4.878	-2.688	-4.155	0.740	-0.068	0.507	-0.320	0.736	-0.068	0.736	-0.063
RMSE	0.060	0.064	0.066	0.066	0.049	0.048	0.049	0.050	0.049	0.048	0.049	0.048
Coverage(%)	83	81	95	93	94	96	95	96	94	96	94	96

S6.3 Semi-synthetic simulation based on the HDL-C–CAD analysis

In our second real data analysis, dmIVW selected the submodel $\mathcal{M}_2$ ($K = 1$, $s_1 = 0$, and free ρ_1). However, as demonstrated in the simulation results of Supplementary Section S6.1, it is possible that the underlying data-generating mechanism involves two groups. This motivates the simulation study in this section. Specifically, we extracted SNP-level summary statistics $\{(\tilde{\beta}_{X,j}, \tilde{\beta}_{Y,j}, \tilde{\sigma}^2_{X,j}, \tilde{\sigma}^2_{Y,j})\}_{j=1}^p$ from the HDL-C–CAD dataset for the top $p = 31$ SNPs with the smallest selection p-values. For each simulation replicate, the estimated SNP–exposure effects $\tilde{\beta}_{X,j}$ were treated as the true exposure effects. The outcome effects were generated under a two-group model with parameters $\pi_1 = 0.6$, $\gamma_1 \approx -0.668$, $s_1 = 0$, $\rho_1 = 1$, and $\pi_2 = 0.4$, $\gamma_2 \approx 0.115$, $s_2 \approx 0.005$, $\rho_2 \approx 1.92$. These parameters were adapted from those estimated using the submodel $\mathcal{M}_{4K_{\max}+4}$, the most flexible submodel when $K = 2$. Under this setup,

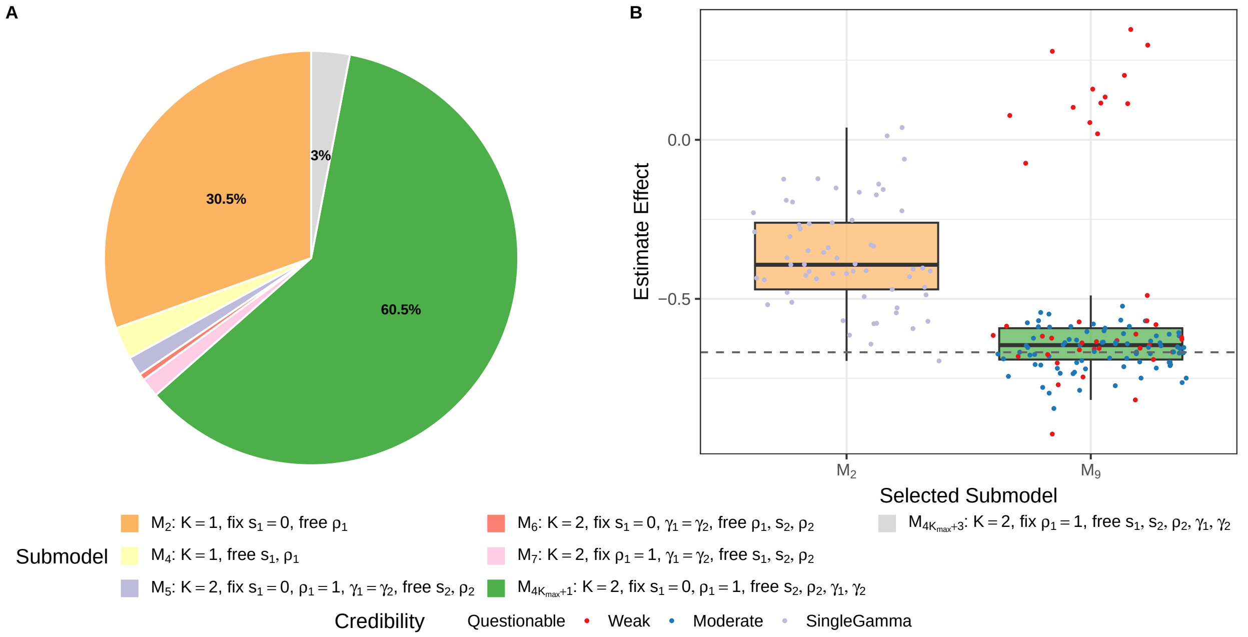

Supplementary Figure S6: **Simulation results based on the HDL-C–CAD dataset with $n_{\text{scale}} = 1$.** A: Frequency of the selected submodels across replicates. B: Estimated causal effects grouped according to the selected model. The vertical dashed line indicates the specified causal effect used in the simulation, and different colors represent different credibility levels.

the first group was the causal group. We considered two settings with $n_{\text{scale}} \in \{1, 2\}$, where n_{scale} controls the effective sample size through rescaling the standard errors as $\tilde{\sigma}_{X,j}/\sqrt{n_{\text{scale}}}$ and $\tilde{\sigma}_{Y,j}/\sqrt{n_{\text{scale}}}$.

As shown in Supplementary Fig. S6A, when $n_{\text{scale}} = 1$, the correct submodel $\mathcal{M}_{4K_{\max}+4}$ was identified in 60.5% of the replicates, whereas in 30.5% of the replicates the simpler model $\mathcal{M}_2$ (a single-group model with dispersion) was selected instead. When the effective sample size was increased to $n_{\text{scale}} = 2$, the proportion of correctly selecting $\mathcal{M}_{4K_{\max}+4}$ increased to 87% as shown in Supplementary Fig. S7A. Supplementary Fig. S6B and Fig. S7B display the estimated causal effects across replicates grouped according to whether the selected submodel was $\mathcal{M}_2$ or $\mathcal{M}_{4K_{\max}+4}$. When $\mathcal{M}_2$ was incorrectly selected, the resulting estimates exhibited noticeable bias. Even when $\mathcal{M}_{4K_{\max}+4}$ was selected, a small proportion of estimates still deviated substantially from the true effect, which may occur when the dominant group was incorrectly identified. We further examined the credibility of these replicates and found that those with extreme estimates tended to have weak or questionable credibility.

To summarize, even when the true data-generating mechanism involves two groups, a simpler one-group model may still be selected with noticeable probability, which can lead to biased causal effect estimates. Encouragingly, this issue can be alleviated by increasing the effective sample size.

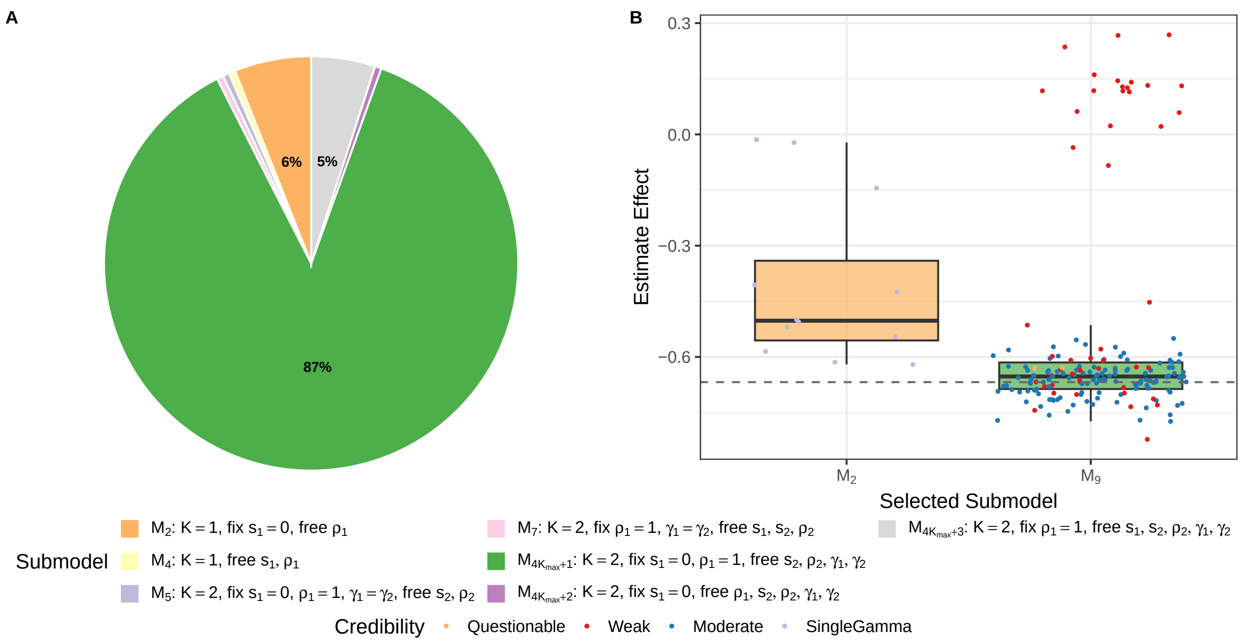

Supplementary Figure S7: **Simulation results based on the HDL-C–CAD dataset with $n_{\mathbf{scale}} = 2$.** A: Frequency of the selected submodels across replicates. B: Estimated causal effects grouped according to the selected model. The vertical dashed line indicates the specified causal effect used in the simulation, and different colors represent different credibility levels.

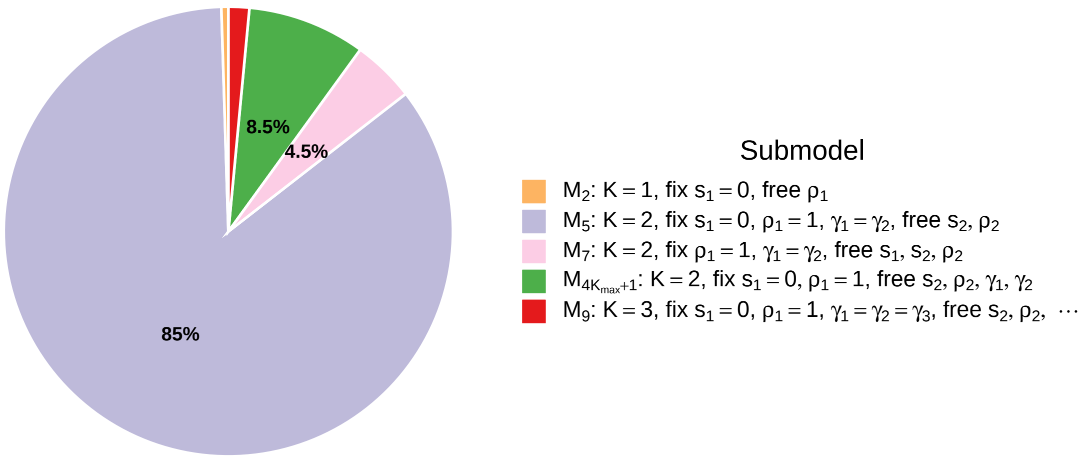

Supplementary Figure S8: **Frequency of the selected submodels across replicates based on the year of schooling-type 2 diabetes dataset with $n_{\text{scale}} = 1$.**

S6.4 Semi-synthetic simulation based on the year of schooling-type 2 diabetes analysis

In our third real data analysis, dmIVW selected the submodel $\mathcal{M}_5$ ($K = 2$, fix $s_1 = 0$, $\rho_1 = 1$, $\gamma_1 = \gamma_2$, free s_2, ρ_2) to infer the effect of years of schooling on type 2 diabetes. Following the simulation procedure described in Supplementary Section S6.3, we conducted a simulation study based on the summary statistics from the corresponding dataset. Specifically, we extracted the top $p = 83$ SNPs with the smallest selection p-values. The outcome effects were generated under $\mathcal{M}_5$, a two-group model with uncorrelated pleiotropy. The parameters were set according to the estimates obtained from fitting $\mathcal{M}_5$ to the real dataset: $\gamma_1 = \gamma_2 \approx -0.116$, $\pi_1 = 0.9$, $s_1 = 0$, $\rho_1 = 1$ for the first group, and $\pi_2 = 0.1$, $s_2 \approx -0.0169$, $\rho_2 \approx 9.35$ for the second group. We set $n_{\text{scale}} = 1$. All other settings followed those in Supplementary Section S6.3. As shown in Supplementary Fig. S8, the correct submodel $\mathcal{M}_5$ was identified with a frequency of approximately 85%. The result provides empirical support for the submodel choice in our real data analysis.

S6.5 Computational cost

We evaluated the computational cost of the proposed procedure based on Setup 1 of our two-group simulation, where the true underlying submodel was $\mathcal{M}_{4K_{\text{max}}+1}$. The true causal effect was set to $\gamma_0 = 0.05$, and the number of candidate IVs was taken as $p \in \{200, 400, 800\}$, while all other settings remained the same as in the main simulation setup.

To account for different model complexity levels, we evaluated several representative submodels. For $K = 2$, we considered three submodels: $\mathcal{M}_5$ corresponding to uncorrelated pleiotropy, $\mathcal{M}_{4K_{\text{max}}+1}$ and $\mathcal{M}_{4K_{\text{max}}+4}$ correspond to the least and most flexible submodels with correlated pleiotropy, respectively. Submodels with $K = 1$ were excluded because they

admit closed-form estimation and introduce negligible computational cost. For $K = 3$, we selected $\mathcal{M}_9$, $\mathcal{M}_{4K_{\max}+5}$, and $\mathcal{M}_{4K_{\max}+8}$ for the same reason. We recorded the runtime for two main steps: (i) fitting each submodel using the EM algorithm with 20 random initializations and (ii) performing the bootstrap procedure used for inference.

All computational times were measured on a personal computer equipped with a 13th Gen Intel(R) Core(TM) i7-13700KF CPU (3.40 GHz), 64 GB RAM, running Windows 10 and R 4.4.1. The runtime distributions across simulation replicates are shown in Supplementary Fig. S9, and the corresponding runtime quantiles are summarized in Supplementary Table S4. Overall, the runtime increased with the number of IVs for most submodels. Among the considered models, the correctly specified model $\mathcal{M}_{4K_{\max}+1}$ exhibited the shortest runtime. In contrast, the submodels $\mathcal{M}_5$ and $\mathcal{M}_9$, both corresponding to uncorrelated pleiotropy structures, required substantially longer computation time. This was likely due to model misspecification, which led to slower convergence of the EM algorithm. For other misspecified models such as $\mathcal{M}_{4K_{\max}+4}$, $\mathcal{M}_{4K_{\max}+5}$, and $\mathcal{M}_{4K_{\max}+8}$, the runtime was comparatively shorter, possibly because the true submodel $\mathcal{M}_{4K_{\max}+1}$ lies within these submodel classes.

In terms of the overall computational cost, both dmIVW and dmIVW$_{\mathsf{MA}}$ require fitting all candidate submodels before selecting the optimal model. After model selection, bootstrap inference is performed only for the selected models: dmIVW selects a single model, whereas dmIVW$_{\mathsf{MA}}$ selects the top $M = 5$ models by default. To illustrate the practical computational cost, we used the median runtimes reported in Supplementary Table S4 for $p = 800$. To approximate the cost across different submodels, we used $\mathcal{M}_5$ and $\mathcal{M}_{4K_{\max}+4}$ to represent submodels with $K = 2$, and $\mathcal{M}_9$ and $\mathcal{M}_{4K_{\max}+8}$ to represent submodels with $K = 3$, each four submodels. The model fitting stage took about 44 seconds. Under a conservative worst-case scenario in which the submodel with the slowest bootstrap runtime was selected, the bootstrap stage for one model would require approximately $200 \times 5.6 \approx 1120$ seconds. In practice, however, the selected submodels should fit the data better. For example, in this setting they are typically $\mathcal{M}_{4K_{\max}+4}$, $\mathcal{M}_{4K_{\max}+5}$, and $\mathcal{M}_{4K_{\max}+8}$, and the bootstrap inference for these submodels can be completed within about three minutes.

S7 Descriptions of Supplementary Figures and Tables

In this section, we provide brief descriptions of supplementary figures and tables that serve as additional supporting material for the main paper but are not discussed in the preceding sections.

The supplementary figures are listed as follows:

- Supplementary Figure S10: Sensitivity analysis of dmIVW and Path with varying p-value selection thresholds under the two-sample design (the first real data application).
- Supplementary Figure S11: Results of various methods for detecting causal relationships between 8 exposures and 30 diseases using 10^{-3} as the p-value selection threshold (the third real data application).
- Supplementary Figure S12: Results of various methods for detecting causal relationships between 8 exposures and 30 diseases using 10^{-4} as the p-value selection threshold (the third real data application).

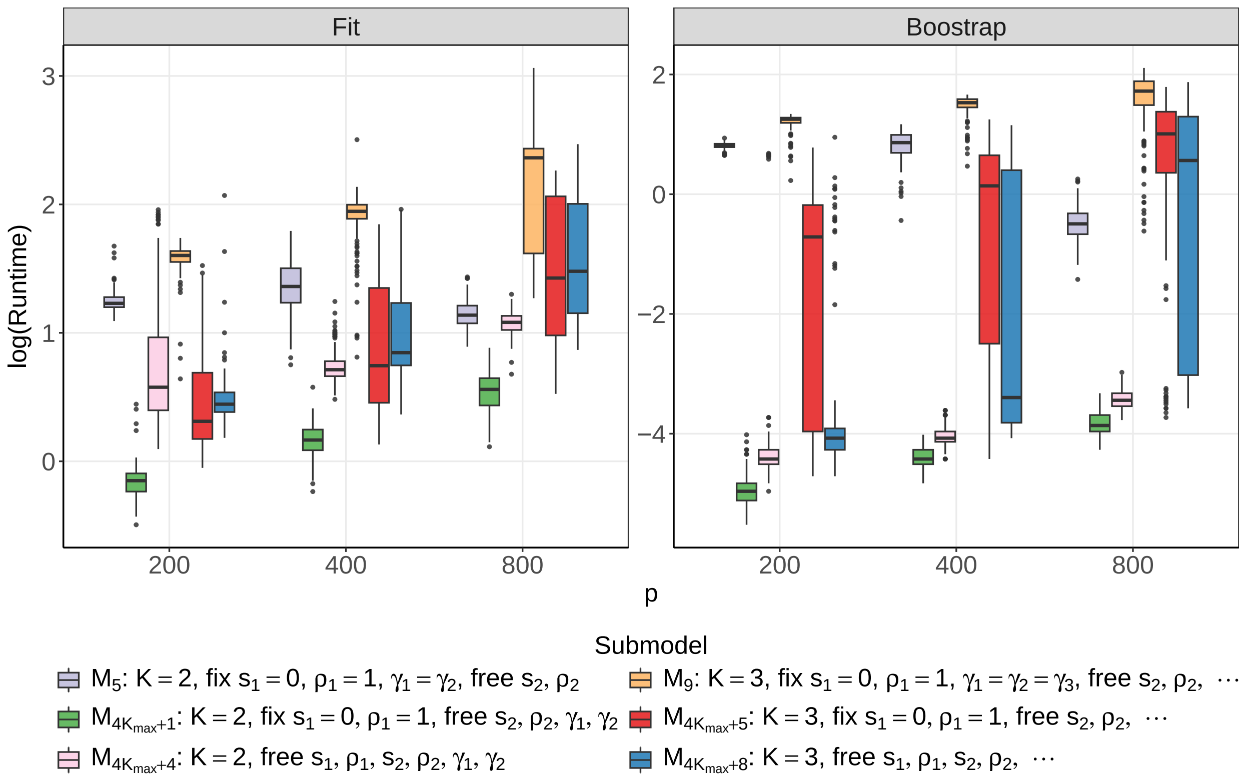

Supplementary Figure S9: **The boxplot of runtime under Setup 1 of our two-group simulation with $p \in \{200, 400, 800\}$.** Our fitting stage uses 20 initial points, and the bootstrap runtime is reported for a single bootstrap run. The simulation was repeated 200 times.

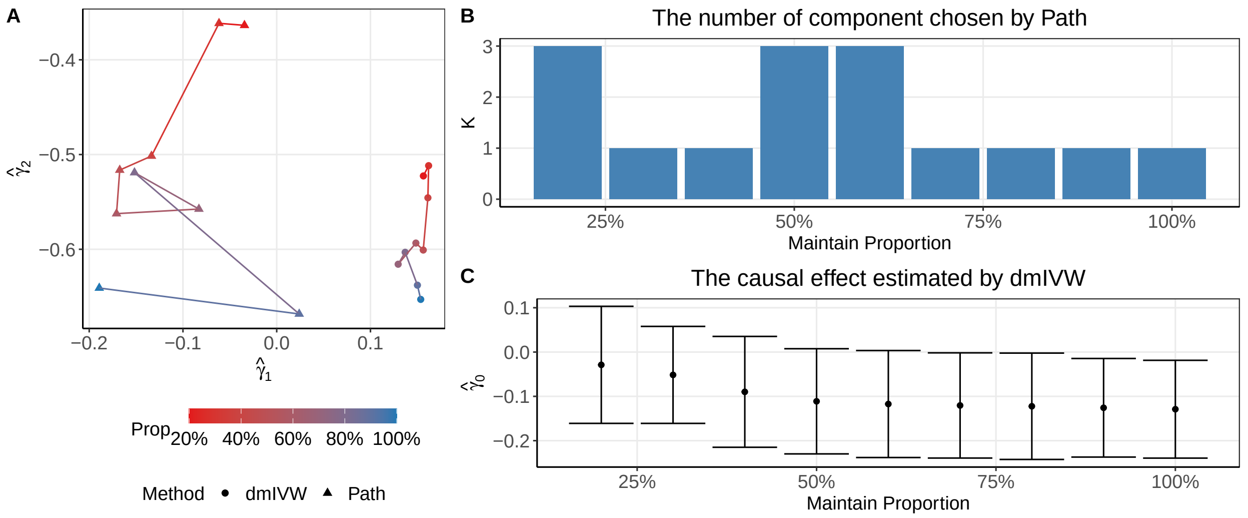

Supplementary Figure S10: **Sensitivity analysis of dmIVW and Path with varying p-value selection thresholds under two-sample design (the first real data application).** The p-value threshold is varied to retain between 20% and 100% of the SNPs. Source data are provided in Supplementary Tables S10 and S11. A: The two groups effects estimated by dmIVW and Path with $K = 2$ at different thresholds. B: The number of groups determined by Path at different thresholds. C: The effects of HDL-C on CAD estimated by dmIVW across varying thresholds.

Supplementary Table S4: **The 25%, 50%, and 75% quantile of runtime under Setup 1 of our two-group simulation with $p \in \{200, 400, 800\}$.** Our fitting stage uses 20 initial points, and the bootstrap runtime is reported for a single bootstrap run. The simulation was repeated 200 times.

Submodel	Quantile	Fit time			Bootstrap time		
		$p = 200$	$p = 400$	$p = 800$	$p = 200$	$p = 400$	$p = 800$
$\mathcal{M}_5$	Q25	3.320	3.437	2.927	2.195	1.996	0.513
	Q50	3.420	3.900	3.120	2.254	2.367	0.610
	Q75	3.590	4.495	3.360	2.321	2.693	0.726
$\mathcal{M}_{4K_{\max}+1}$	Q25	0.790	1.090	1.545	0.006	0.011	0.019
	Q50	0.860	1.180	1.750	0.007	0.012	0.021
	Q75	0.910	1.280	1.910	0.008	0.014	0.025
$\mathcal{M}_{4K_{\max}+4}$	Q25	1.488	1.940	2.780	0.011	0.016	0.029
	Q50	1.780	2.040	2.950	0.012	0.017	0.032
	Q75	2.625	2.180	3.102	0.014	0.019	0.036
$\mathcal{M}_9$	Q25	4.720	6.608	5.045	3.292	4.264	4.429
	Q50	4.965	7.000	10.620	3.500	4.610	5.597
	Q75	5.140	7.370	11.410	3.592	4.892	6.598
$\mathcal{M}_{4K_{\max}+5}$	Q25	1.190	1.577	2.665	0.019	0.083	1.430
	Q50	1.365	2.105	4.165	0.490	1.149	2.739
	Q75	1.992	3.855	7.868	0.835	1.915	3.966
$\mathcal{M}_{4K_{\max}+8}$	Q25	1.467	2.110	3.167	0.014	0.022	0.049
	Q50	1.560	2.330	4.390	0.017	0.033	1.756
	Q75	1.710	3.430	7.420	0.020	1.493	3.654

The supplementary tables are listed as follows:

- Supplementary Table S5: A summary of the main notation used in this work.
- Supplementary Table S6: The absolute bias and MSE, averaged over different values of γ_0 and all replications.
- Supplementary Table S7: Comparison of uncertainty estimates with different bootstrap sizes under Setup 1 of our two-group IV simulation.
- Supplementary Table S8: Comparisons of methods under individual data simulation with $\gamma_0 = 0.1$.
- Supplementary Table S9: Comparisons of methods under individual data simulation with $\gamma_0 = 0$ and one invalid IV.

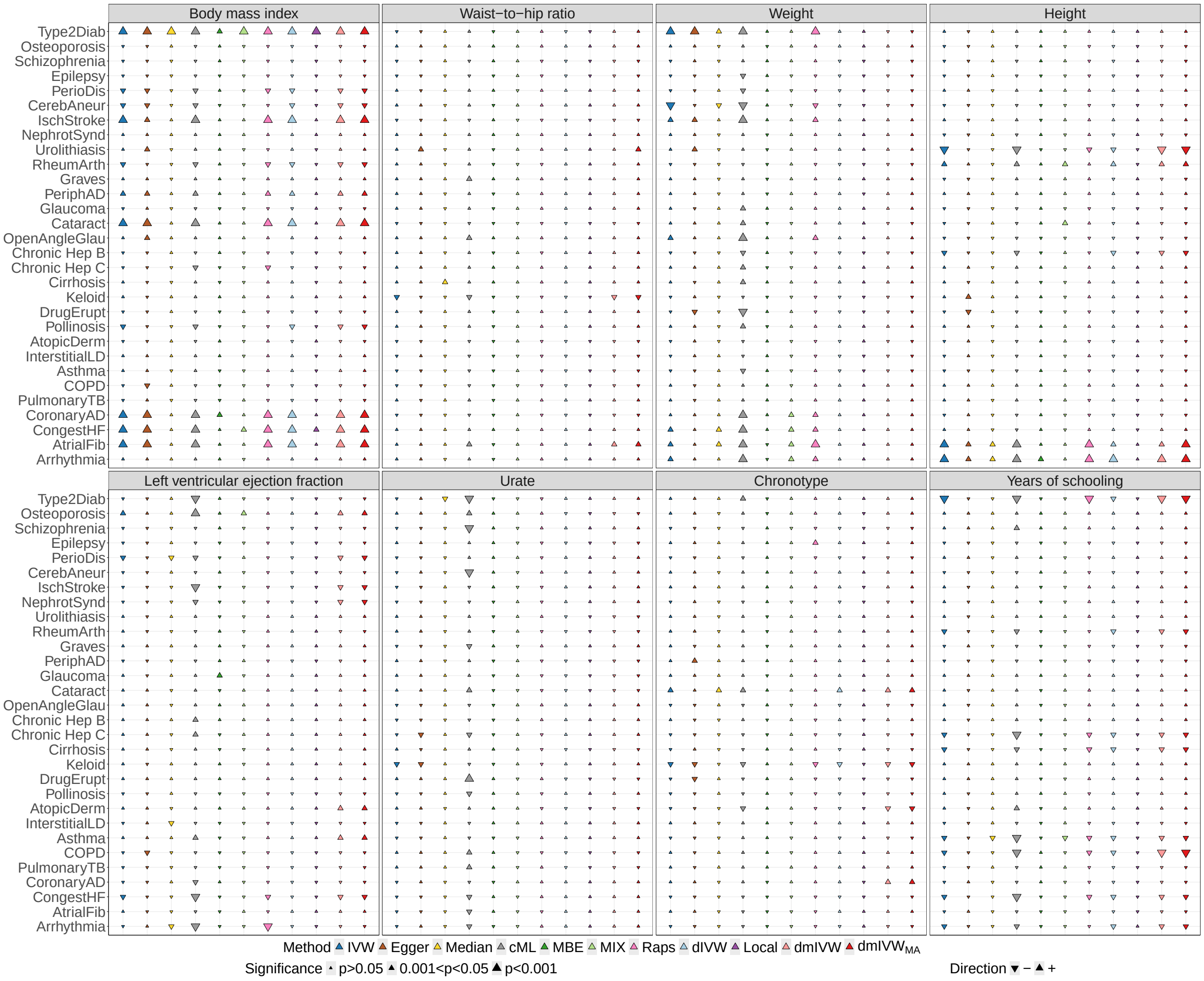

Supplementary Figure S11: **Results of various methods for detecting causal relationships between 8 exposures and 30 diseases using 10^{-3} as the p-value selection threshold (the third real data application)**. The causal effect is the logOR of disease per SD-unit increase in risk factor. Source data of results are provided in Supplementary Tables S17.

- Supplementary Table S10: Results of dmIVW and Path using $K = 2$ with varying p-value selection thresholds under two and three-sample design (the first real data application).
- Supplementary Table S11: Results of dmIVW with varying p-value selection thresholds under two and three-sample designs (the first real data application).
- Supplementary Table S12: The estimated causal effects $\hat{\gamma}_0$ and the corresponding confidence interval at different selection thresholds obtained by IVW, Egger, Median, MIX, MBE, ConMIX and cML (the second real data application).
- Supplementary Table S13: The estimated causal effects $\hat{\gamma}_0$ and the corresponding con-

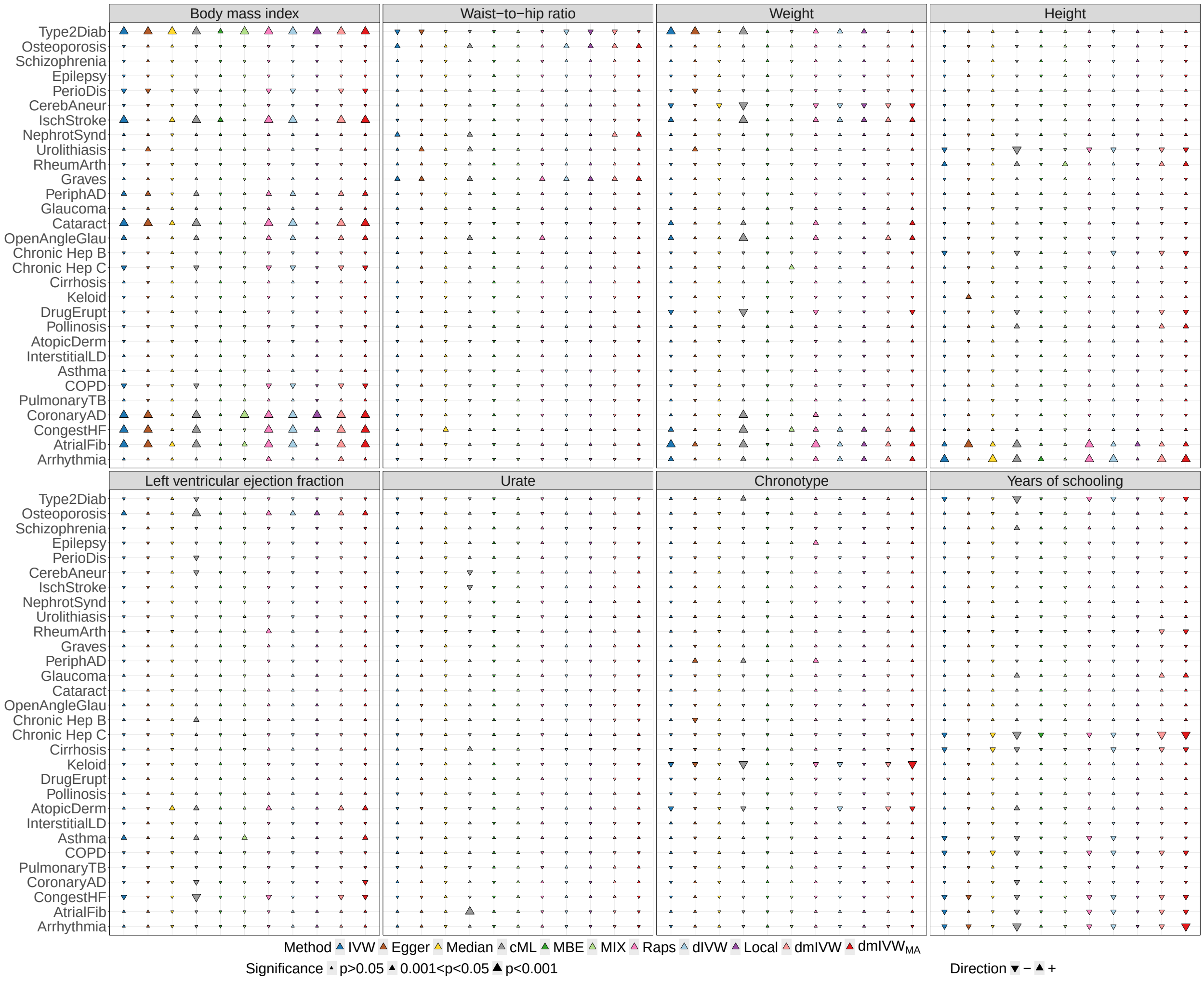

Supplementary Figure S12: **Results of various methods for detecting causal relationships between 8 exposures and 30 diseases using 10^{-4} as the p-value selection threshold (the third real data application)**. The causal effect is the logOR of disease per SD-unit increase in risk factor. Source data of the results are provided in Supplementary Table S18.

fidence interval at different selection thresholds obtained by MIXIE, Raps, dIVW, Local, dmIVW, and dmIVW$_{MA}$ (the second real data application).

- Supplementary Table S14[1]: The details about the dataset used in our first real data application.
- Supplementary Table S15[1]: The details about the dataset used in our second real data application.
- Supplementary Table S16[1]: The details about the dataset used in our third real data application.

[1] Also available at https://doi.org/10.6084/m9.figshare.30281053.v1

- Supplementary Table S17[1]: Detailed results of various methods for detecting causal relationships between 8 exposures and 30 diseases using 10^{-3} as the p-value selection threshold (the third real data application).
- Supplementary Table S18[1]: Detailed results of various methods for detecting causal relationships between 8 exposures and 30 diseases using 10^{-4} as the p-value selection threshold (the third real data application).
- Supplementary Table S19[1]: Detailed results of various methods for detecting causal relationships between 8 exposures and 30 diseases using 10^{-5} as the p-value selection threshold (the third real data application).

Supplementary Table S5: **Summary of the main notation used in the paper.** Unless otherwise specified, the estimate of a parameter is denoted by adding a hat (e.g., $\hat{\gamma}_k$, $\hat{s}_k$, $\hat{\rho}_k$).

Symbol	Description
**Data and observed quantities**	
M_i	Exposure for individual i
Y_i	Outcome for individual i
p	Number of instrumental variables (IVs)
G_{ij}	Genetic variant j for individual i
$\beta_{M,j}$	True association between the jth IV and the exposure
$\beta_{Y,j}$	True association between the jth IV and the outcome
$\hat{\beta}_{M,j}$	Estimated association between the jth IV and the exposure
$\hat{\beta}_{Y,j}$	Estimated association between the jth IV and the outcome
$\sigma^2_{M,j}$	Estimated variance of $\hat{\beta}_{M,j}$
$\sigma^2_{Y,j}$	Estimated variance of $\hat{\beta}_{Y,j}$
Individual-level structural model	
K	Number of IV groups in the mixture model
$\mathcal{J}_k$	Set of IVs belonging to the kth group
U_i^k	kth shared heritable confounder for individual i
$\delta_{M,j}$	Direct effect of IV j on exposure
$\delta_{U,j}$	Direct effect of IV j on the group-specific confounder U_i^k
$\theta_{M,k}$	Effect of confounder U^k on exposure
$\theta_{Y,k}$	Effect of confounder U^k on outcome
$\epsilon_{M,i}$	Random error in the exposure equation
$\epsilon_{Y,i}$	Random error in the outcome equation
$\epsilon_{U,i}$	Random error in the confounder equation
c_k	Projection coefficient used in pleiotropy decomposition
Working model $\mathcal{M}$	
U_j	Latent group membership indicator for jth IV
γ (or γ_0)	True causal effect of the exposure on the outcome
γ_k	Effect parameter associated with the kth IV group
ψ_j	Residual pleiotropic effect for jth IV
s_k	Deviation parameter capturing directional pleiotropy in kth group
ρ_k	Dispersion parameter capturing balanced pleiotropy in kth group
π_k	Proportion of IVs belonging to kth group
w_{jk}	Probability that jth IV belongs to kth group

Supplementary Table S6: The absolute bias and MSE, averaged over different values of γ_0 and all replications.

(a) Averaged Absolute Bias

Method	$\text{dIVW}_{\text{valid}}$	dIVW	Local	Raps	dmIVW	dmIVW_{MA}
Setup 1	0.00077	0.50079	0.00579	0.16434	0.00006	**0.00005**
Setup 2	0.00014	1.24901	0.02193	1.15905	0.00125	**0.00009**
Setup 3a	0.00143	0.00289	0.04053	0.00298	**0.00021**	0.00030
Setup 3b	0.00014	0.00038	0.00140	0.00026	**0.00009**	0.00017
Setup 4a	0.00145	0.00095	**0.00082**	0.00131	0.00116	0.00105
Setup 4b	**0.00014**	0.00040	0.00389	0.00018	0.00081	0.00082

(b) Averaged MSE

Method	$\text{dIVW}_{\text{valid}}$	dIVW	Local	Raps	dmIVW	dmIVW_{MA}
Setup 1	**0.00015**	0.25188	0.00037	0.17816	0.00017	0.00017
Setup 2	**0.00019**	1.56249	0.00189	1.34786	0.00317	0.00025
Setup 3a	0.00063	0.00338	0.10971	0.00439	**0.00012**	**0.00012**
Setup 3b	0.00019	0.00248	0.00046	0.00286	0.00011	**0.00010**
Setup 4a	0.00063	0.00050	0.00112	**0.00048**	0.00050	0.00049
Setup 4b	**0.00019**	0.00035	0.00108	0.00030	0.00028	0.00028

Supplementary Table S7: **Comparison of uncertainty estimates with different bootstrap sizes under Setup 1 of our two-group IV simulation.** The table reported the average estimated standard errors and the overlap of the corresponding 95% confidence intervals (CIs) compared with the reference results obtained using $B = 800$ bootstrap samples. The overlap was measured by the Jaccard index between two confidence intervals, defined as the ratio of the length of their intersection to the length of their union. For each quantity, the table reported the mean across simulation replicates, with Monte Carlo standard errors shown in parentheses.

Bootstrap	Standard Error		Overlap of CI	
Size	dmIVW	dmIVW_{MA}	dmIVW	dmIVW_{MA}
50	0.01245 (0.00009)	0.01241 (0.00009)	0.9273 (0.0039)	0.9270 (0.0038)
100	0.01256 (0.00006)	0.01230 (0.00007)	0.9470 (0.0028)	0.9407 (0.0029)
200	0.01261 (0.00005)	0.01245 (0.00005)	0.9608 (0.0019)	0.9552 (0.0023)
400	0.01248 (0.00004)	0.01258 (0.00004)	0.9654 (0.0018)	0.9673 (0.0018)
800	0.01247 (0.00003)	0.01252 (0.00003)	1.0000 (0.0000)	1.0000 (0.0000)

Supplementary Table S8: **Comparisons of methods under individual data simulation with $\gamma_0 = 0.1$.** Results were based on 1000 replications, summarizing bias (multiplied by 100 for ease of comparison), root mean square error (RMSE), coverage, and power. We considered 30 valid IVs. The confidence level was set as 95%.

Criterion	Method													
	IVW	dIVW	Egger	Raps	Median	MBE	APSS	cML	Local	MIX	MIXIE	ConMIX	dmIVW	dmIVW_{MA}
Bias($\times$100)	-0.029	0.013	-0.107	-0.004	-0.043	-0.038	0.009	**0.001**	0.013	-0.193	-0.005	0.405	-0.044	-0.019
RMSE	**0.012**	0.012	0.027	0.012	0.016	0.027	0.012	0.012	0.012	0.060	0.013	0.017	0.019	0.018
Coverage(%)	95.60	95.70	95.70	95.70	97.70	99.30	99.30	94.70	95.70	89.80	94.80	85.80	90.40	97.40
Power(%)	100.00	100.00	94.50	100.00	100.00	97.00	100.00	100.00	100.00	62.80	100.00	100.00	97.50	97.80

Supplementary Table S9: **Comparisons of methods under individual data simulation with $\gamma_0 = 0$ and one invalid IV.** The data were generated in the same way as the uncorrelated pleiotropy setting in the individual-data simulations, with $p_{\text{invalid}} = 1$. The magnitude of the pleiotropic effect was varied through τ_Y to examine the impact of increasing violation strength. The results are organized in the same way as in Table 1 of the main paper.

τ_Y	Criterion	Method													
		IVW 6/0/3	dIVW 6/0/3	Egger 3/6/0	Raps 6/3/0	Median 6/3/0	MBE 8/1/0	APSS 6/0/3	cML 8/2/0	Local 6/0/3	MIX 3/5/1	MIXIE 3/6/0	ConMIX 6/3/0	dmIVW 3/6/0	dmIVW_{MA} 6/3/0
$\sqrt{0.1}/p$	Bias($\times$100)	0.499	0.501	0.079	0.200	0.196	**-0.033**	0.503	0.248	0.501	-0.247	0.370	0.042	0.231	0.241
	RMSE	0.013	0.013	0.030	**0.012**	0.016	0.024	0.013	0.012	0.013	0.080	0.013	0.015	0.021	0.019
	Coverage(%)	95.10	95.00	93.60	95.70	97.30	98.20	95.40	93.70	95.00	93.20	92.30	89.10	88.50	96.20
$\sqrt{0.2}/p$	Bias($\times$100)	0.716	0.719	0.061	0.088	0.196	-0.104	0.724	0.109	0.719	-0.329	0.358	**-0.022**	0.203	0.245
	RMSE	0.014	0.014	0.033	**0.012**	0.016	0.023	0.014	0.012	0.014	0.049	0.015	0.015	0.023	0.021
	Coverage(%)	95.30	95.00	93.30	94.90	97.40	98.40	95.20	93.80	95.00	96.10	91.30	90.10	88.40	96.10
$\sqrt{0.3}/p$	Bias($\times$100)	0.882	0.886	0.051	-0.284	0.199	-0.110	0.897	**0.007**	0.886	-0.513	0.224	-0.033	0.120	0.184
	RMSE	0.015	0.015	0.036	0.091	0.016	0.024	0.015	**0.012**	0.015	0.028	0.015	0.014	0.022	0.021
	Coverage(%)	95.08	95.08	93.17	94.98	97.39	97.79	95.38	94.58	95.08	96.99	91.47	89.86	90.96	96.59

Supplementary Table S10: Results of dmIVW and Path using $K = 2$ with varying p-value selection thresholds under two and three-sample design (the first real data application).

Method		dmIVW				Path			
Type of Design		2 sample		3 sample		2 sample		3 sample	
maintain proportion	p_{maintain}	$\hat{\gamma}_1$	$\hat{\gamma}_2$	$\hat{\gamma}_1$	$\hat{\gamma}_2$	$\hat{\gamma}_1$	$\hat{\gamma}_2$	$\hat{\gamma}_1$	$\hat{\gamma}_2$
20%	31	0.156	-0.523	0.115	-0.668	-0.034	-0.364	0.144	-0.566
30%	46	0.162	-0.512	0.160	-0.585	-0.062	-0.362	-0.109	-0.483
40%	61	0.161	-0.546	0.154	-0.696	-0.134	-0.502	-0.133	-0.524
50%	76	0.156	-0.601	0.160	-0.691	-0.167	-0.516	-0.014	-0.502
60%	91	0.148	-0.593	0.154	-0.683	-0.171	-0.562	-0.086	-0.527
70%	106	0.130	-0.616	0.139	-0.687	-0.083	-0.558	-0.132	-0.552
80%	121	0.137	-0.603	0.150	-0.641	-0.152	-0.519	-0.109	-0.685
90%	136	0.150	-0.638	0.155	-0.665	0.024	-0.668	-0.163	-0.670
100%	151	0.154	-0.653	0.154	-0.653	-0.189	-0.641	-0.177	-0.667

Supplementary Table S12: The estimated causal effects $\hat{\gamma}_0$ and the corresponding confidence interval at different selection thresholds obtained by IVW, Egger, Median, MIX, MBE, ConMIX and cML (the second real data application).

Outcome	$\alpha^{\text{sel}}_{\text{value}}$	Method						
		IVW	Egger	Median	MIX	MBE	ConMIX	cML
AIS	–	0.12(0.04,0.20)	0.13(0.02,0.24)	0.17(-0.01,0.34)	0.06(-1.18,1.30)	-4.97(-16.38,6.44)	0.12(0.04,0.20)	–
	10^{-1}	0.11(0.02,0.19)	0.12(0.00,0.23)	0.11(-0.07,0.30)	0.09(-1.63,1.81)	-4.87(-13.76,4.03)	0.10(0.02,0.18)	–
	10^{-3}	0.11(0.01,0.20)	0.11(-0.03,0.24)	0.07(-0.14,0.28)	0.13(-1.09,1.35)	-0.12(-1.11,0.87)	0.12(0.03,0.21)	–
	10^{-5}	0.12(-0.01,0.24)	0.20(0.02,0.38)	0.00(-0.26,0.27)	0.06(-0.66,0.78)	0.15(-0.21,0.50)	0.14(0.02,0.26)	–
	10^{-7}	0.15(-0.00,0.30)	0.19(-0.04,0.43)	0.11(-0.17,0.39)	0.24(-0.78,1.26)	0.20(-0.15,0.55)	0.21(0.06,0.35)	–
	10^{-9}	0.13(-0.04,0.31)	0.25(-0.03,0.54)	0.08(-0.21,0.37)	0.18(-0.19,0.55)	0.26(-0.09,0.61)	0.20(0.05,0.34)	–
CAD	–	0.32(0.20,0.43)	0.39(0.23,0.54)	0.23(0.00,0.46)	0.43(0.13,0.73)	0.74(-3.89,5.37)	0.35(0.23,0.44)	–
	10^{-1}	0.29(0.17,0.40)	0.38(0.21,0.54)	0.26(0.01,0.50)	0.43(0.18,0.68)	0.20(-2.85,3.25)	0.33(0.23,0.44)	–
	10^{-3}	0.29(0.14,0.44)	0.41(0.19,0.63)	0.34(0.07,0.61)	0.39(0.12,0.66)	0.64(-0.18,1.47)	0.34(0.21,0.47)	–
	10^{-5}	0.30(0.10,0.49)	0.46(0.17,0.74)	0.20(-0.12,0.51)	0.36(0.14,0.58)	0.64(0.08,1.19)	0.35(0.19,0.50)	–
	10^{-7}	0.29(0.06,0.52)	0.51(0.16,0.86)	0.22(-0.12,0.57)	0.37(0.14,0.60)	0.33(0.06,0.61)	0.34(0.17,0.52)	–
	10^{-9}	0.32(0.03,0.61)	0.60(0.10,1.09)	0.28(-0.04,0.60)	0.40(0.17,0.63)	0.50(0.18,0.82)	0.43(0.25,0.60)	–
SBP	–	0.32(0.10,0.53)	0.45(0.11,0.79)	0.15(-0.12,0.42)	0.44(0.16,0.72)	0.77(0.35,1.18)	0.40(0.26,0.60)	0.54(0.41,0.68)
	10^{-1}	0.32(0.10,0.53)	0.45(0.11,0.79)	0.15(-0.12,0.42)	0.44(0.16,0.72)	0.77(0.35,1.18)	0.40(0.26,0.60)	0.54(0.41,0.68)
	10^{-3}	0.32(0.10,0.53)	0.45(0.11,0.79)	0.15(-0.12,0.42)	0.44(0.16,0.72)	0.77(0.35,1.18)	0.40(0.26,0.60)	0.54(0.41,0.68)
	10^{-5}	0.40(0.20,0.60)	0.41(0.06,0.76)	0.39(0.11,0.66)	0.38(-0.47,1.23)	0.70(0.26,1.14)	0.42(0.23,0.72)	0.45(0.30,0.59)
	10^{-7}	0.35(0.08,0.63)	0.60(0.06,1.14)	0.31(0.01,0.62)	-0.06(-0.80,0.68)	0.37(-0.07,0.80)	0.45(0.26,0.73)	0.40(0.22,0.58)
	10^{-9}	0.32(-0.02,0.65)	0.93(0.18,1.68)	0.33(0.04,0.62)	-0.08(-0.96,0.80)	0.44(-0.00,0.87)	0.44(0.11,0.75)	0.32(0.11,0.53)
BMI	–	0.93(0.90,0.95)	0.91(0.86,0.95)	0.91(0.86,0.96)	0.96(-3.94,5.87)	0.74(0.47,1.01)	0.91(0.89,0.93)	–
	10^{-1}	0.93(0.90,0.95)	0.91(0.86,0.95)	0.91(0.86,0.96)	0.96(-3.94,5.87)	0.74(0.47,1.01)	0.91(0.89,0.93)	–
	10^{-3}	0.95(0.92,0.98)	0.91(0.86,0.96)	0.96(0.91,1.01)	1.00(0.80,1.20)	0.92(0.81,1.02)	0.95(0.92,0.97)	–
	10^{-5}	0.99(0.95,1.02)	0.88(0.81,0.95)	1.01(0.95,1.07)	1.00(0.42,1.58)	0.96(0.84,1.07)	1.02(0.97,1.05)	–
	10^{-7}	0.99(0.94,1.03)	0.90(0.81,1.00)	1.00(0.93,1.07)	0.95(0.79,1.11)	0.93(0.81,1.05)	1.01(0.96,1.07)	–
	10^{-9}	0.98(0.93,1.03)	0.93(0.82,1.03)	1.03(0.95,1.11)	0.97(0.80,1.14)	0.99(0.90,1.08)	1.01(0.97,1.06)	–

Supplementary Table S11: Results of dmIVW with varying p-value selection thresholds under two and three-sample designs (the first real data application).

maintain proportion	p_{maintain}	two-sample design				three-sample design			
		$\widehat{\gamma}_0$	$\widehat{\rho}_0$	CI_{lower}	CI_{upper}	$\widehat{\gamma}_0$	$\widehat{\rho}_0$	CI_{lower}	CI_{upper}
20%	31	-0.029	3.042	-0.161	0.103	-0.060	3.606	-0.214	0.094
30%	46	-0.052	2.691	-0.161	0.058	-0.065	2.957	-0.209	0.079
40%	61	-0.090	2.880	-0.215	0.035	-0.079	2.805	-0.207	0.049
50%	76	-0.111	2.654	-0.230	0.007	-0.107	2.665	-0.228	0.014
60%	91	-0.117	2.702	-0.238	0.003	-0.116	2.479	-0.235	0.003
70%	106	-0.121	2.585	-0.239	-0.002	-0.126	2.518	-0.247	-0.004
80%	121	-0.122	2.604	-0.242	-0.002	-0.134	2.602	-0.244	-0.024
90%	136	-0.126	2.413	-0.237	-0.015	-0.136	2.454	-0.257	-0.015
100%	151	-0.129	2.375	-0.239	-0.019	-0.129	2.375	-0.241	-0.017

Supplementary Table S13: The estimated causal effects $\hat{\gamma}_0$ and the corresponding confidence interval at different selection thresholds obtained by MIXIE, Raps, dIVW, Local, dmIVW, and $\mathsf{dmIVW_{MA}}$ (the second real data application).

Outcome	$\alpha^{\text{sel}}_{\text{value}}$	Method					
		MIXIE	Raps	dIVW	Local	dmIVW	$\mathsf{dmIVW_{MA}}$
AIS	–	0.12(0.04,0.20)	0.16(0.06,0.27)	0.15(0.05,0.25)	0.18(-0.15,0.52)	0.15(0.05,0.24)	0.15(0.05,0.24)
	10^{-1}	0.11(0.02,0.19)	0.14(0.03,0.24)	0.12(0.02,0.22)	0.18(-0.09,0.45)	0.12(0.03,0.22)	0.12(0.03,0.22)
	10^{-3}	0.11(0.01,0.20)	0.13(0.02,0.24)	0.12(0.01,0.22)	0.13(-0.22,0.48)	0.12(0.01,0.22)	0.12(0.01,0.22)
	10^{-5}	0.12(-0.01,0.24)	0.13(-0.00,0.27)	0.12(-0.01,0.26)	0.19(-0.06,0.44)	0.11(-0.01,0.24)	0.12(-0.01,0.24)
	10^{-7}	0.15(-0.00,0.30)	0.17(0.02,0.33)	0.15(-0.00,0.31)	0.15(-0.00,0.31)	0.12(-0.03,0.26)	0.13(-0.02,0.28)
	10^{-9}	0.13(-0.04,0.31)	0.16(-0.03,0.34)	0.14(-0.05,0.32)	0.14(-0.05,0.32)	0.14(-0.01,0.28)	0.11(-0.05,0.28)
CAD	–	0.32(0.20,0.43)	0.39(0.26,0.52)	0.36(0.23,0.50)	0.33(-0.13,0.79)	0.36(0.23,0.50)	0.36(0.24,0.49)
	10^{-1}	0.29(0.17,0.40)	0.35(0.22,0.49)	0.32(0.18,0.45)	0.32(-0.13,0.78)	0.32(0.19,0.45)	0.32(0.19,0.44)
	10^{-3}	0.29(0.14,0.44)	0.34(0.17,0.50)	0.30(0.15,0.46)	0.36(-0.01,0.72)	0.30(0.14,0.47)	0.30(0.14,0.46)
	10^{-5}	0.30(0.10,0.49)	0.35(0.16,0.54)	0.30(0.10,0.50)	0.30(0.10,0.50)	0.30(0.10,0.51)	0.30(0.10,0.51)
	10^{-7}	0.29(0.06,0.52)	0.35(0.12,0.57)	0.29(0.06,0.53)	0.29(0.06,0.53)	0.29(0.05,0.53)	0.29(0.04,0.54)
	10^{-9}	0.32(0.03,0.61)	0.39(0.12,0.66)	0.33(0.03,0.62)	0.33(0.03,0.62)	0.33(0.04,0.61)	0.33(0.04,0.62)
SBP	–	0.39(0.28,0.51)	0.56(0.42,0.70)	0.36(0.11,0.62)	0.36(0.11,0.62)	0.41(0.19,0.62)	0.42(0.20,0.63)
	10^{-1}	0.39(0.28,0.51)	0.56(0.42,0.70)	0.36(0.11,0.62)	0.36(0.11,0.62)	0.41(0.18,0.63)	0.42(0.20,0.63)
	10^{-3}	0.39(0.28,0.51)	0.56(0.42,0.70)	0.36(0.11,0.62)	0.36(0.11,0.62)	0.41(0.20,0.61)	0.42(0.19,0.64)
	10^{-5}	0.40(0.25,0.55)	0.44(0.23,0.66)	0.43(0.21,0.65)	0.43(0.21,0.65)	0.43(0.23,0.63)	0.43(0.21,0.65)
	10^{-7}	0.52(0.28,0.75)	0.39(0.11,0.68)	0.37(0.08,0.65)	0.37(0.08,0.65)	0.37(0.08,0.65)	0.34(0.00,0.67)
	10^{-9}	0.65(0.46,0.85)	0.36(0.02,0.70)	0.32(-0.02,0.67)	0.32(-0.02,0.67)	0.32(-0.04,0.68)	0.32(-0.02,0.67)
BMI	–	0.93(0.90,0.95)	1.00(0.97,1.04)	1.01(0.98,1.04)	0.98(0.87,1.09)	1.01(0.98,1.04)	1.01(0.98,1.04)
	10^{-1}	0.93(0.90,0.95)	1.00(0.97,1.04)	1.01(0.98,1.04)	0.98(0.88,1.09)	1.01(0.98,1.03)	1.01(0.97,1.04)
	10^{-3}	0.95(0.92,0.98)	1.01(0.97,1.04)	1.01(0.97,1.04)	0.99(0.90,1.09)	1.01(0.98,1.03)	1.01(0.97,1.04)
	10^{-5}	0.99(0.95,1.02)	1.01(0.97,1.05)	1.02(0.98,1.05)	1.01(0.90,1.12)	1.01(0.98,1.05)	1.01(0.98,1.05)
	10^{-7}	0.99(0.94,1.03)	1.00(0.95,1.05)	1.01(0.96,1.05)	1.00(0.95,1.05)	1.00(0.95,1.04)	1.00(0.95,1.04)
	10^{-9}	0.98(0.93,1.03)	1.00(0.95,1.05)	1.00(0.95,1.05)	1.00(0.95,1.05)	0.98(0.93,1.03)	0.98(0.93,1.03)

References

[1] Bowden, J., Davey Smith, G., and Burgess, S. (2015), "Mendelian randomization with invalid instruments: Effect estimation and bias detection through Egger regression," *International Journal of Epidemiology*, 44, 512–525.

[2] Bowden, J., Davey Smith, G., Haycock, P. C., and Burgess, S. (2016), "Consistent estimation in Mendelian randomization with some invalid instruments using a weighted median estimator," *Genetic Epidemiology*, 40, 304–314.

[3] Burgess, S., Butterworth, A., and Thompson, S. G. (2013), "Mendelian randomization analysis with multiple genetic variants using summarized data," *Genetic Epidemiology*, 37, 658–665.

[4] Burgess, S., Foley, C. N., Allara, E., Staley, J. R., and Howson, J. M. (2020), "A robust and efficient method for Mendelian randomization with hundreds of genetic variants," *Nature Communications*, 11, 376.

[5] Foley, C. N., Mason, A. M., Kirk, P. D. W., and Burgess, S. (2020), "MR-Clust: Clustering of genetic variants in Mendelian randomization with similar causal estimates," *Bioinformatics*, 37, 531–541.

[6] Grant, A. J. and Burgess, S. (2022), "An efficient and robust approach to Mendelian randomization with measured pleiotropic effects in a high-dimensional setting," *Biostatistics*, 23, 609–625.

[7] Hartwig, F. P., Davey Smith, G., and Bowden, J. (2017), "Robust inference in summary data Mendelian randomization via the zero modal pleiotropy assumption," *International Journal of Epidemiology*, 46, 1985–1998.

[8] Iong, D., Zhao, Q., and Chen, Y. (2024), "A latent mixture model for heterogeneous causal mechanisms in Mendelian randomization," *The Annals of Applied Statistics*, 18, 966–990, 25.

[9] Jiang, L. et al. (2019), "Constrained instruments and their application to Mendelian randomization with pleiotropy," *Genetic Epidemiology*, 43, 373–401.

[10] Lin, Z., Deng, Y., and Pan, W. (2021), "Combining the strengths of inverse-variance weighting and Egger regression in Mendelian randomization using a mixture of regressions model," *PLOS Genetics*, 17, 1–25.

[11] McLachlan, G. J. and Krishnan, T. (2008), "Lower bound maximization," in *The EM Algorithm and Extensions*, pp. 269–287.

[12] Pardo, L. (2006), "Divergence measures: Definition and properties," in *Statistical Inference Based on Divergence Measures*, Chapman and Hall/CRC, pp. 1–54.

[13] Qi, G. and Chatterjee, N. (2019), "Mendelian randomization analysis using mixture models for robust and efficient estimation of causal effects," *Nature Communications*, 10, 1941.

[14] Rees, J. M. B., Wood, A. M., Dudbridge, F., and Burgess, S. (2019), "Robust methods in Mendelian randomization via penalization of heterogeneous causal estimates," *PLOS ONE*, 14, e0222362.

[15] Sanderson, E. et al. (2022), "Mendelian randomization," *Nature Reviews Methods Primers*, 2, 6.

[16] Wang, J. et al. (2021), "Causal inference for heritable phenotypic risk factors using heterogeneous genetic instruments," *PLoS Genetics*, 17, e1009575.

[17] Xu, S., Fung, W. K., and Liu, Z. (2021), "MRCIP: A robust Mendelian randomization method accounting for correlated and idiosyncratic pleiotropy," *Briefings in Bioinformatics*, 22, bbab019.

[18] Xu, Z. and Li, S. (2026), "Leveraging local distributions in Mendelian randomization: Uncer-

tain opinions are invalid," *Statistica Sinica*, 36.

[19] Xue, H., Shen, X., and Pan, W. (2021), "Constrained maximum likelihood-based Mendelian randomization robust to both correlated and uncorrelated pleiotropic effects," *The American Journal of Human Genetics*, 108, 1251–1269.

[20] Yakowitz, S. J. and Spragins, J. D. (1968), "On the identifiability of finite mixtures," *The Annals of Mathematical statistics*, 39, 209–214.

[21] Ye, T., Shao, J., and Kang, H. (2021), "Debiased inverse-variance weighted estimator in two-sample summary-data Mendelian randomization," *The Annals of Statistics*, 49, 2079–2100, 22.

[22] Zhao, Q., Wang, J., Hemani, G., Bowden, J., and Small, D. S. (2020), "Statistical inference in two-sample summary-data Mendelian randomization using robust adjusted profile score," *The Annals of Statistics*, 48, 1742–1769, 28.

[23] Zhu, Z. et al. (2018), "Causal associations between risk factors and common diseases inferred from GWAS summary data," *Nature Communications*, 9, 1–12.
